# Fragment-based discovery of a new family of non-peptidic small-molecule cyclophilin inhibitors with potent antiviral activities

**DOI:** 10.1038/ncomms12777

**Published:** 2016-09-22

**Authors:** Abdelhakim Ahmed-Belkacem, Lionel Colliandre, Nazim Ahnou, Quentin Nevers, Muriel Gelin, Yannick Bessin, Rozenn Brillet, Olivier Cala, Dominique Douguet, William Bourguet, Isabelle Krimm, Jean-Michel Pawlotsky, Jean- François Guichou

**Affiliations:** 1 INSERM U955 ‘Pathophysiology and Therapy of Chronic Viral Hepatitis and Related Cancers', Hôpital Henri Mondor, Université Paris-Est, 51 avenue du Maréchal de Lattre de Tassigny, 94010 Créteil, France; 2 CNRS UMR5048, Centre de Biochimie Structurale, Université de Montpellier, 29 rue de Navacelles, 34090 Montpellier, France; 3 INSERM U1054, Centre de Biochimie Structurale, Université de Montpellier, 29 rue de Navacelles, 34090 Montpellier, France; 4 Institut des Sciences Analytiques, CNRS UMR5280, Université Lyon 1, École Nationale Supérieure de Lyon, 5 rue de la Doua, 69100 Villeurbanne, France; 5 National Reference Center for Viral Hepatitis B, C and Delta, Department of Virology, Hôpital Henri Mondor, Université Paris-Est, 51 avenue du Maréchal de Lattre de Tassigny, 94010 Créteil, France

## Abstract

Cyclophilins are peptidyl-prolyl *cis/trans* isomerases (PPIase) that catalyse the interconversion of the peptide bond at proline residues. Several cyclophilins play a pivotal role in the life cycle of a number of viruses. The existing cyclophilin inhibitors, all derived from cyclosporine A or sanglifehrin A, have disadvantages, including their size, potential for side effects unrelated to cyclophilin inhibition and drug–drug interactions, unclear antiviral spectrum and manufacturing issues. Here we use a fragment-based drug discovery approach using nucleic magnetic resonance, X-ray crystallography and structure-based compound optimization to generate a new family of non-peptidic, small-molecule cyclophilin inhibitors with potent *in vitro* PPIase inhibitory activity and antiviral activity against hepatitis C virus, human immunodeficiency virus and coronaviruses. This family of compounds has the potential for broad-spectrum, high-barrier-to-resistance treatment of viral infections.

## Abstract (web summary)

Cyclophilins play a key role in the life cycle of many viruses and represent important drug targets for broad-spectrum antiviral therapies. Here, the authors use fragment-based drug discovery to develop non-peptidic inhibitors of human cyclophilins with high activity against replication of a number of viral families.

## Graphical abstract

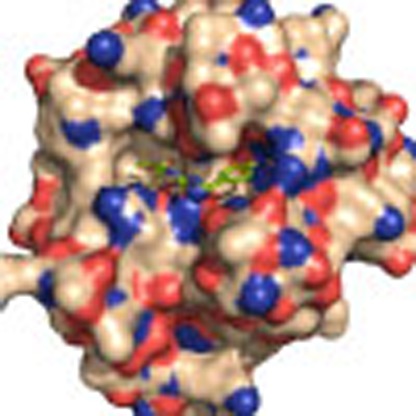

Over the past decades, an increasing number of viruses causing unexpected illnesses and epidemics among humans, wildlife and livestock has emerged. These outbreaks have seriously stretched local and national resources in the economically developed world, whereas the capacity to control emerging diseases remains limited in poorer regions where many of them have their origin. A number of virus-specific antiviral agents have been developed and commercialized since the early 1980s. These agents, including drugs that specifically inhibit members of the *Herpesviridae* family, influenza viruses, human immunodeficiency virus (HIV), hepatitis B virus (HBV) and, more recently, hepatitis C virus (HCV), had a major medical impact^1^. However, the development costs of specific antiviral agents are extremely high and there are many other medically important viral infections that require efficacious therapies. Thus, there is an urgent need for new families of broad-spectrum antiviral agents, that is, antiviral agents that are active against a number of different viral families^2^. Such compounds should target mechanisms common to different families of viruses, such as cellular components and/or functions involved in their life cycles. The cellular proteins cyclophilins have been shown to play a key role in the life cycle of a number of different viral families. In addition, cyclophilin inhibitors were reported to inhibit the replication of different viruses, both *in vitro* and *in vivo*^3^,^4^. Thus, cyclophilins represent an attractive target for broad-spectrum antiviral inhibition.

Cyclophilins are peptidyl-prolyl *cis/trans* isomerases (PPIase) that catalyse the interconversion of the two energetically preferred conformers (*cis* and *trans*) of the planar peptide bond preceding an internal proline residue. Seventeen human cyclophilins have been identified thus far. Among them, cyclophilin A (CypA) is present in the cytosol and involved in protein folding, trafficking, immunomodulation and cell signalling^5^. The extracellular fraction of CypB is involved in cell–cell communications and inflammatory signalling. CypD is localized in the matrix of the mitochondria and acts as a key regulator of the opening of the mitochondrial permeability transition pore (mPTP), which plays a major role in calcium efflux from mitochondria to the cytosol and can lead to mitochondrial swelling and cell death^6^,^7^,^8^,^9^. The function of most of the remaining cyclophilins is unknown^10^,^11^.

Cyclophilins share a common domain of ∼109 amino acids, the cyclophilin-like domain, surrounded by domains unique to each member of the family and associated with their subcellular compartmentalization and functional specialization^11^. Cyclophilins adopt an eight-stranded anti-parallel P-barrel structure (Fig. 1a)^10^. Their PPIase catalytic groove is formed by a mixture of hydrophobic, aromatic and polar residues, including Arg55, Phe60, Met61, Gln63, Asn102, Phe113, Trp121, Leu122 and His126 (Fig. 1a,b). The existence of a deep pocket contiguous to the canonical catalytic site, called the ‘gatekeeper' pocket, has been revealed (Fig. 1c,d)^10^. The gatekeeper pocket might contribute to substrate-binding specificity, its access being determined by gatekeeper residues at its surface.

Several cyclophilins, including principally but not exclusively CypA, have been shown to play a pivotal role in the life cycle of a number of viruses, including HIV, HCV, dengue virus, Japanese encephalitis virus, yellow fever virus, coronaviruses, HBV, cytomegalovirus, influenza A virus, enteroviruses and so on^3^,^12^,^13^,^14^. Cyclosporine A (CsA) and non-immunosuppressive macrocyclic analogues of CsA and of sanglifehrin A (SfA) potently inhibit cyclophilin PPIase activity by binding its catalytic site (Fig. 1d). They have shown *in vitro* effectiveness against HIV, HCV and HBV replication^3^. A CsA analogue, alisporivir, showed potent anti-HCV activity *in vivo*^15^,^16^, but its clinical development was halted during the Phase III programme due to severe adverse events unrelated to cyclophilin inhibition.

The existing cyclophilin inhibitors, all derived from CsA or SfA, have disadvantages, including their size resulting in poor cell permeability, the risk of drug–drug interactions, their unclear antiviral spectrum (only the effect on HCV has been reproducibly demonstrated), manufacturing issues they raise (synthesis of alisporivir is a complicated hemi-synthetic pathway starting with CsA that includes 12 steps, some of which use highly reactive and dangerous compounds) and their potential for side effects unrelated to cyclophilin inhibition. In addition to inhibiting cyclophilin PPIase activity, CsA was, for instance, shown to inhibit the mitogen-activated protein kinase pathway and affect transforming growth factor-β1 levels, whereas CsA and its non-immunosuppressive derivatives were reported to inhibit ABC transporters (for example, PgP, MRP and BCRP) and stimulate antigen presentation by enhancing major histocompatibility complex-I surface expression^17^,^18^,^19^,^20^,^21^,^22^. Alisporivir was associated with acute pancreatitis cases, including one fatal case, when combined with pegylated interferon-α in a Phase III trial, in patients infected with HCV, through mechanisms that are unknown. Thus, new families of cyclophilin inhibitors are urgently needed.

Fragment-based drug discovery (FBDD) is based on the identification of very small molecules (fragments) that are subsequently expanded or linked together to generate drug leads with therapeutic activity^23^,^24^. Here we use an FBDD approach using nucleic magnetic resonance (NMR) and X-ray crystallography to generate a new family of non-peptidic, small-molecule cyclophilin inhibitors, unrelated to CsA or SfA, with potent *in vitro* PPIase inhibitory activity and antiviral activity against several families of viruses responsible for frequent human infections.

## Results

### Fragment screening

In total, 34,409 fragments were computationally docked into the canonical active site and the gatekeeper pocket of CypD by means of the FlexX programme. Forty-four fragments were selected based on their mode of interaction. Their ability to interact with CypD was further studied by means of NMR spectroscopy. Ten fragment hits with low-affinity dissociation constants (millimolar range) were identified (Supplementary Fig. 1). Their scaffolds and proline-mimicking motifs were used to select *in cerebro* a set of 52 derivative fragments for subsequent X-ray crystallographic experiments. Apo CypD crystals were soaked with each of the 52 fragments. X-ray structures of CypD complexed with 14 fragments were obtained.

Supplementary Fig. 2 shows the chemical structures of the 14 binding fragments. Four fragments (**9**, **11**, **12** and **13**) bound the catalytic site of CypD, whereas five fragments (**6**, **15**, **16**, **17** and **18**) bound the gatekeeper pocket. Fragment **14** bound between the two sites. Finally, four fragments (**5**, **19**, **20** and **21**) were nonspecific multibinders. The density map of each fragment is shown in Supplementary Fig. 3 and at (https://figshare.com/articles/Stereo_views_of_cocrystal_structures_of_cyclophilin_inhibitors_with_cyclophilin_D/3490493).

The ability of each fragment to inhibit cyclophilin activity *in vitro* was assessed in cell-free enzyme assays for CypA, CypB and CypD. The half-maximal inhibitory concentrations (IC_50_) of the 14 fragments were >5 mM in all instances.

### Fragment selection for linking

Among the 14 fragment hits, the final selection of compounds **6** and **13** for subsequent compound optimization was based on a number of criteria, including their ligand efficiency, their ability to access key regions, their synthetic tractability and the possibility to link them to generate compounds binding both the catalytic site and the gatekeeper pocket.

**Figure 2:**
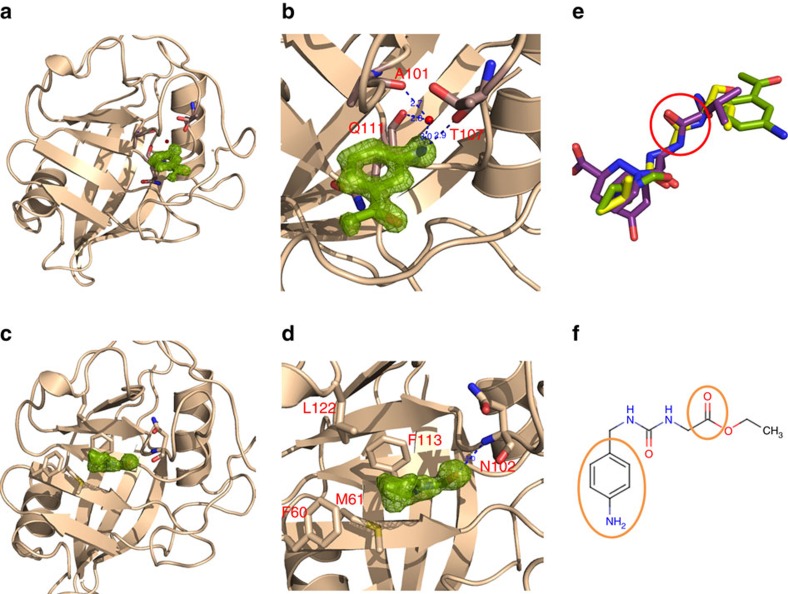
Cocrystal structures of fragment hits with CypD and linking strategy. (**a**,**b**) The crystal structure shows fragment **6** bound to the gatekeeper pocket, predominantly through a hydrogen bond with Thr107. The green mesh and surface represent the electron density map of fragment **6** (2*F*_o_*–F*_c_ omit map contoured at 1.0*σ*). The gatekeeper pocket is zoomed in **b**. (**c**,**d**) The crystal structure shows fragment **13** bound to the catalytic site, predominantly through a hydrogen bond with Asn102. The green mesh and surface represent the electron density map of fragment **13** (2*F*_o_–*F*_c_ omit map contoured at 1.0*σ*). The catalytic site of CypD is zoomed in **d**. (**e**) Superimposition of PDB 1CWA (CypA-CsA) and 1YND (CypA-SfA) with cocrystals CypD-fragment **6** and CypD-fragment **13**. CsA is represented with yellow sticks. SfA is represented by purple sticks. Fragments **6** and **13** are represented by green sticks. The red circle shows the urea moiety used to link fragments **6** and **13**. (**f**) Chemical structure of compound **22**, generated by linking fragments **6** and **13** with a urea moiety. The orange circle shows key atoms from fragments **6** and **13**.

The X-ray crystallographic structure of CypD complexed with fragment **6**, solved at a resolution of 1.10 Å, showed that this fragment deeply buried into the gatekeeper pocket, a hydrophilic region (Fig. 2a), and its amino group displaced a water molecule present in this pocket in the apo form of CypD. Within the gatekeeper pocket, fragment **6** made one direct hydrogen bond with Thr107 and two hydrogen bonds with Ala101 and Gln111 through a water molecule (Fig. 2b).

The crystallographic structure of CypD in complex with fragment **13** was solved at a resolution of 1.35 Å (Fig. 2c). In addition to its hydrophobic contacts with Phe60, Met61, Phe113 and Leu122 within the catalytic site of CypD, fragment **13** directly interacted with Asn102 *via* a hydrogen bond (Fig. 2d).

### Linking strategy

Superimposition of fragments **6** and **13** with the known structures of CsA and SfA, two cyclophilin inhibitors, suggested that a urea moiety could be used as a linker between the two fragments, because of one hydrogen bond with Gln63 and two with Asn102 (Fig. 2e). Compound **22**, consisting of fragments **6** and **13** connected by a urea moiety, was thus generated (Fig. 2f). This compound inhibited CypD activity, with an IC_50_ of 6.2±3.7 μM (Table 1).

**Figure 3:**
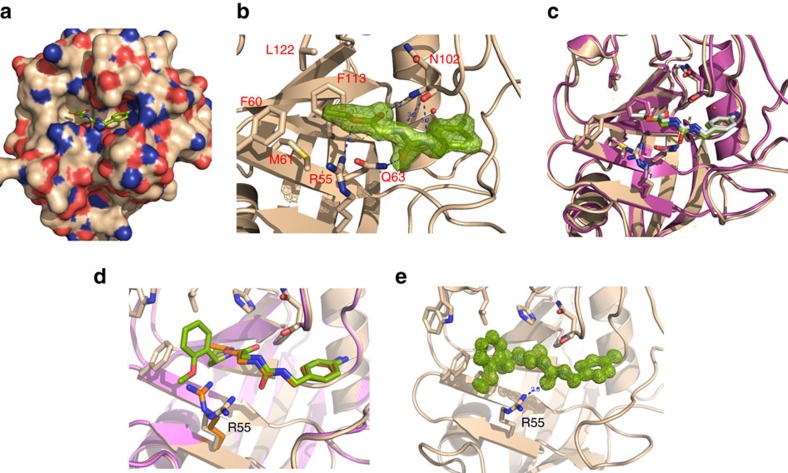
Co-crystal structure of compounds 22 and 24 with CypA and CypD. (**a**) Surface representation of CypD in complex with compound **22**, showing occupation of both the catalytic site (left) and the gatekeeper pocket (right) of CypD. (**b**) Zoom into the catalytic site of CypD showing the urea moiety linker of compound **22** making four hydrogen bonds with Arg55, Gln63 and Asn102. The green mesh and surface represents the electron density map of compound **22** (2*F*_o_–*F*_c_ omit map contoured at 1.0*σ*). (**c**) Superimposition of the CypD-compound **22** (pink for CypD, green sticks for compound **22**) and the CypA-compound **22** (purple for CypA, white sticks for compound **22**) co-crystals, showing identical binding modes. (**d**) Superimposition of the CypD-compound **24** cocrystal (pink for CypD, green sticks for compound **24**) with the CypD-compound **22** co-crystal (purple for CypD, orange sticks for compound **22**). The side chain of Arg55 is shown in stick format (pink for compound **24** and orange for compound **22**). Compound **24** shares the same mode of CypD binding as compound **22** and its methoxy group pushes Arg55 to create a hydrogen bond with the urea moiety of the compound. (**e**) Cartoon representations of CypD in complex with compound 24, showing occupation of the gatekeeper pocket and the catalytic site of CypD. The side chain of Arg55 is represented in stick format to show the interaction with the urea moiety. The green mesh and surface represent the electron density maps (2*F*_o_–*F*_c_ omit map contoured at 1.0*σ*).

The structure of CypD complexed with compound **22**, obtained at a resolution of 1.93 Å, revealed binding to both the gatekeeper pocket and the catalytic site (Fig. 3a and b). Compound **22** maintained the key interactions of fragments **6** and **13** within their respective pockets, while making one, one and two additional hydrogen bonds with Arg55, Gln63 and Asn102, respectively (Fig. 3b). The binding mode of compound **22** to CypD was confirmed by means of NMR experiments. The CypD ^15^N-heteronuclear single quantum coherence (HSQC) spectrum revealed significant chemical shift perturbations on ligand binding only for residues located at or near the catalytic site and gatekeeper pocket, respectively (Supplementary Fig. 4). Cross-peaks of Gly151, Asn144 and Asn145 disappeared on ligand addition, likely to be due to intermediate chemical exchange, demonstrating that these residues are part of the binding site and the corresponding amide protons are located at proximity of the ligand.

Compound **22** also potently inhibited CypA and CypB (IC_50s_: 13.1±5.9 and 6.1±3.8 μM, respectively). These findings were confirmed by isothermal titration calorimetry experiments showing reduced conformational flexibility on binding of these proteins (Supplementary Fig. 5). Finally, superimposition of the crystallographic structures of CypA and CypD complexed with compound **22** showed identical binding modes (Fig. 3c and https://figshare.com/articles/Stereo_views_of_cocrystal_structures_of_cyclophilin_inhibitors_with_cyclophilin_D/3490493).

### Structure-based lead optimization of the compounds

Structure-guided optimization was used to improve cyclophilin affinity and stability of the compounds. As ester functions are often associated with low biological stability, the first step was to replace the ester function of compound **22**, which makes a key hydrogen bond with Arg55, without affecting the cyclophilin inhibitory potency. All designed compounds lacking the ester function lost their interaction with Arg55, resulting in a drastic decrease of cyclophilin inhibition (IC_50_ >500 μM). This was the case of compound **23** (Table 1), although this compound retained all of the other key interactions of compound **22** (Supplementary Fig. 6 and https://figshare.com/articles/Stereo_views_of_cocrystal_structures_of_cyclophilin_inhibitors_with_cyclophilin_D/3490493). Based on the observation that in the CypA–SfA complex (PDB code 1YND) the side chain of Arg55 is pushed by an oxygen atom of SfA, a phenyl–pyrrolidine moiety was added to generate compound **24** (Table 1). As shown in Fig. 3d, compound **24** shared the same binding mode as compound **22**. In addition, the methoxy of the phenyl–pyrrolidine moiety displaced the side chain of Arg55, making a hydrogen bond with the carbonyl moiety of the urea motif (Fig. 3e and https://figshare.com/articles/Stereo_views_of_cocrystal_structures_of_cyclophilin_inhibitors_with_cyclophilin_D/3490493). Compound **24** proved to be a potent inhibitor of CypA, CypB and CypD PPIase activities, with IC_50s_ of 2.8±0.6, 1.2±0.1 and 11.4±3.0 μM, respectively.

A series of phenyl–pyrrolidine derivatives were then synthesized, including compounds **25**–**31** (Table 1). The crystal structures of CypD in complex with compounds **26**, **27**, **28** and **29** showed the same binding mode (Supplementary Fig. 7 and https://figshare.com/articles/Stereo_views_of_cocrystal_structures_of_cyclophilin_inhibitors_with_cyclophilin_D/3490493), in keeping with NMR experiments shown in Supplementary Fig. 4B. Interestingly, the thiomethyl groups of compounds **27** and **29** were involved in a transient contact with Arg55 Nɛ. Table 1 shows the inhibitory effects of these compounds on CypA, CypB and CypD PPIase activities in enzyme assays.

As compounds with an aniline motif have been reported to potentially bear toxic properties^25^,^26^, a very large number of chemical modifications aimed at replacing compound **22**'s aniline motif, while retaining its PPIase inhibitory activity, were made. These included replacement of the amino group by the following: halogens (F, Cl and Br), hydroxyl, methoxy, ester and acetamide; replacement by pyridines, aminopyridines and heterocycles that sometimes contained an amine function, and fused or non-fused 6:6 or 6:5 bicycles; and substitution with methoxy or alkyl groups. The list and structure of the compounds tested for PPIase inhibitory activity is shown in Supplementary Table 1. Among them, only compound **32**, in which the aniline motif was replaced by an amino-2,3-dihydro-1H-inden-1-yl, retained the PPIase inhibitory potency of its parent compound, with IC_50_s of 7.4±6.8, 8.7±1.5 and 12.8±2.5 μM for CypA, CypB and CypD, respectively (Table 1). In addition, replacement of compound **31**'s aniline motif by a 3-amino-pyridine led to the generation of compound **33**, which, although less active than its parent compound, retained significant PPIase inhibitory activities, with IC_50_s of 4.2±1.6, 2.2±1.2 and 7.7±0.8 μM for CypA, CypB and CypD, respectively (Table 1).

On the other hand, macrocyclization was attempted to stabilize the bioactive conformation of the compounds. However, macrocyclic compound **S69** (Supplementary Table 1) was inactive against cyclophilin PPIase activity. In contrast, the addition of a phenyl ring between the urea and carbonyl of compound **31** stabilized the bioactive conformation by a π–π interaction between the two phenyl moieties, leading to an at least threefold gain of anti-PPIase activity. Ultimately, compound **31** was the most potent cyclophilin inhibitor generated, with IC_50_s of 0.1±0.07, 0.08±0.04 and 0.2±0.08 μM for CypA, CypB and CypD, respectively (Table 1).

### Broad-spectrum antiviral activity

Huh7 cells harbouring an HCV genotype 1b replicon were treated with increasing concentrations of compounds **26**, **27**, **29**, **30**, **31**, **32** and **33**. As shown in Table 2, all of them inhibited HCV replicon replication in a dose-dependent manner, with EC_50s_ ranging from 0.4 to 8.4 μM. Representative inhibitory curves are shown for compound **31** in Supplementary Fig. 8. The EC_50s_ of CsA and compound **31**, the most potent inhibitor, were in the same range (0.3±0.1 μM versus 0.4±0.3 μM, respectively) and ∼40-fold higher than the EC_50_ of the CsA derivative alisporivir (0.01±0.0007 μM; Table 2). The compounds were not cytotoxic at their effective concentrations.

The antiviral activities of compounds **26**, **27**, **29** and **30** were also assessed against HIV-IIIb replication in MT4 cells. All tested compounds inhibited HIV replication better than CsA, with EC_50s_ ranging from 3.6 to 15 μM. The compounds were not cytotoxic at their effective concentrations (Table 2).

Finally, the antiviral activities of compounds **26**, **27**, **29, 30**, **31**, **32** and **33** were assessed against human coronavirus 229E (HCoV-229E) in MRC-5 cells. The seven tested compounds inhibited HCoV-229E replication with EC_50s_ ranging from 7.2 to 71.5 μM, without associated cytotoxicity (Table 2).

### Effect of NS5A protein substitutions on anti-HCV activity

The D320E and R318H substitutions in domain II of the NS5A protein have been reported to be associated with slightly reduced HCV susceptibility to the antiviral action of CsA and its nonimmunosuppressive derivatives^27^. The corresponding nucleotide substitutions were introduced in a genotype 1b subgenomic replicon by means of site-directed mutagenesis and the antiviral activity of compounds **27**, **29**, **30** and **31** was measured in the mutated replicons in comparison with a wild-type replicon. The results shown in Table 3 indicate that substitutions D320E and R318H very slightly reduced susceptibility to these compounds (1.0- to 2.8-fold change in EC_50_), in the same order as the change in CsA and alisporivir susceptibility they induce.

### Lack of calcineurin inhibition properties

In addition to its anti-cyclophilin activity, CsA displays potent immunosuppressive properties through the formation of a stable ternary complex in a 1:1:1 stoichiometry with calcineurin, CypA and CsA, resulting in the inhibition of calcineurin phosphatase activity^28^. Although the new family of inhibitors is unrelated to CsA, preincubation studies were performed with the binary complex formed of CypA and compound **31**, to determine whether this complex inhibits calcineurin phosphatase activity. As shown in Supplementary Fig. 9, a dose-dependent decrease of calcineurin activity was observed in the presence of increasing concentrations of the CypA–CsA complex, used as a positive control of inhibition. In contrast, no inhibition was observed in the presence of the binary complex of CypA and compound **31**. In addition, CsA potently inhibited interleukin (IL)-2 production in stimulated immortalized T lymphocytes (Jurkat cells) with an EC_50_ of 0.005 μM, whereas compound **31** had no effect on IL-2 production (EC_50_ >20 μM).

### *In vitro* metabolism

Compounds **29** and **30** were assessed for their *in vitro* metabolism before compound **31** was synthesized. The results are summarized in Supplementary Table 2. The measured octanol/water partitioning (LogD) values for compounds **29** and **30** were 2.5 and 2.6, respectively. Both compounds were very soluble at pH 7.4 and at pH 1.0. They were not degraded after incubation in PBS for 24 h at 37 °C and slightly degraded after incubation in HCl 0.1 N at pH 1.0 for 24 h at 37 °C (98% of compound **29** and 92% of compound **30** remaining at 24 h). Permeability was high for both compounds, whereas the efflux was negligible for compound **30** and low for compound **29** in monolayers of Caco-2 cells, suggesting a high absorption potential for the family.

Binding to human plasma proteins was 85.0 and 85.1% for compounds **29** and **30**, respectively. The high recovery indicated satisfactory plasma stability. Therefore, both compounds were predicted to display low shifts in biological potency in the presence of human plasma compared with cell culture medium. The metabolic stabilities were studied using human hepatic microsomal fractions in the presence of NADPH, to support oxidative metabolism. Compounds **29** and **30** were metabolized in microsomal fractions with *in vitro* half-lives of 21.6 and 35.5 min, respectively, yielding moderate predicted clearances.

Neither compound **29** nor compound **30** was metabolized by CYP1A2 or CYP2B6. Both compounds were metabolized at the highest rate by CYP2C19. Compound **30**, but not compound **29**, was metabolized by CYP3A4. The relative contributions of the individual enzymes to the metabolism of the compounds could not be determined from these experiments, but the data suggested that compounds **29** and **30** were predominantly metabolized by CYP2C19 enzymes.

Neither compound **29** nor compound **30** significantly inhibited CYP1A2, CYP2B6, CYP2C9, CYP2C19 or CYP2D6. Both compounds were moderate inhibitors of CYP2C8 and potent inhibitors of CYP3A, suggesting possible drug–drug interactions through the inhibition of CYP3A enzymes.

## Discussion

In this study, we used FBDD to create a new family of non-peptidic, small-molecule cyclophilin inhibitors, unrelated to CsA or SfA, with potent PPIase inhibitory activity and with potent antiviral effectiveness against HCV, HIV and coronaviruses *in vitro*.

FBDD has emerged as an effective approach for drug discovery nearly 20 years ago^29^. FBDD indeed generated a number of new drug classes, including some that reached the medical market^30^. FBDD is based on screening of low-molecular-weight molecules with minimal chemical complexity that bind subpockets within a target site. These fragments typically bind with low affinity, in the μM to mM range. They represent suitable starting points for structurally guided ‘hit-to-lead optimization'. The fragment-based approach offers better coverage of the chemical diversity space than high-throughput screening, while offering the advantage of structure-based chemical optimization^31^.

The cyclophilin PPIase catalytic groove is formed by a mixture of hydrophobic, aromatic and polar residues that are highly conserved across different cyclophilins. Notably, the side-chain guanidine group of the essential Arg55 is hydrogen bonded to the prolyl nitrogen of the substrate. It promotes isomerization by weakening the double-bond properties of the peptide bond^32^. Targeting this residue is thus crucial to achieve potent inhibition of PPIase activity. CsA, its non-immunosuppressive cyclic analogues and SfA are large macrocycle compounds (molecular weight >1,000 g mol^−1^). They bind the cyclophilin catalytic site and potently inhibit PPIase activity^33^. However, the shortcomings of these families of compounds in clinical practice emphasize the need for the development of small-molecule cyclophilin inhibitors. As the cyclophilin catalytic site is not a buried pocket, the design of small ligands with a molecular weight <500 g mol^−1^ is nevertheless challenging^33^. A deeper pocket, the ‘gatekeeper pocket‘, has been identified in close vicinity to the PPIase catalytic site^10^. This pocket could contribute to substrate binding specificity through gatekeeper residues located at its surface that restrict substrate accessibility. Thus, the gatekeeper pocket appears as an interesting target close to the catalytic site, suitable for a fragment-based approach targeting both sites combined with a linking strategy.

The most common approach for chemical optimization is the ‘fragment growing strategy', which permits a multistep optimization of ligand efficiency and size within the binding site^30^. Alternatively, the ‘linking strategy‘ is constrained by the size of the original fragments and that of the linker, therefore resulting in a rapid buildup of atoms in a single step. Furthermore, conformational strain and flexibility mean that an energy price often needs to be paid to achieve optimal linking of the fragments^34^. In spite of these challenges, and because we were targeting two different subpockets, we decided to use this approach to link two fragments binding the catalytic and gatekeeper pockets, respectively, to substantially improve the stability and binding affinity of the resulting molecule. By means of structure-based drug design, we selected a linker consisting of a urea moiety that mimicked key interactions of both CsA and SfA with cyclophilins without altering the binding mode of the two fragments alone. Our results thus show that linking fragments that bind adjacent sites is a powerful approach for complex targets when an optimal linker can be found.

Compound **31** displayed potent *in vitro* activity against CypA, CypB and CypD PPIase activities. Unlike CsA, the new family of cyclophilin inhibitors had no effect of calcineurin phosphatase activity, which is responsible for CsA immunosuppressive properties. In addition, early pharmacological studies and *in vitro* cellular toxicity experiments suggest that the compound family is druggable. As the presence of an aniline motif may be associated with toxicity in animal models or man^25^,^26^, several rounds of chemical optimization were performed to remove the aniline motif. This led to the generation of compounds **32** and **33** that do not have an aniline motif but retain both anti-PPIase and anti-viral activity. The risk related to the aniline motif must be balanced with the severity of the target disease (cost/benefit ratio) and the required duration of administration. For instance, what would be considered a major safety issue when treating a benign disease requiring long-term administration would be unimportant in case of short-term treatment of a severe, eventually life-threatening acute infection. Thus, lead compounds both with and without the aniline motif will be moved forward throughout preclinical development.

The compounds described here represent the first non-peptidic, small-molecule cyclophilin inhibitor family with broad-spectrum antiviral properties described thus far. Based on a previous work from our group^35^, Chinese authors reported a series of thiourea-based inhibitors targeting both the HIV-1 capsid and human CypA, which inhibited assembly and uncoating of the viral capsid^36^. However, the low CypA-binding affinity of the compounds strongly contradicted the claimed PPIase inhibitory activity and the reported effect on HIV could be exclusively related to the capsid ligand properties of the compounds in this work.

Another series of small-molecule CypA inhibitors was reported in 2009 as a result of *de novo* drug design^37^. We synthesized their two ‘best' compounds and purchased one of them (TMN-355) from Tocris Bioscience, to test their ability to inhibit PPIase activity, and HCV and coronavirus replication *in vitro*. As shown in Supplementary Table 3, none of these compounds exhibited any biological activity in the models. Furthermore, we could not obtain any crystallographic structure of the compounds complexed with cyclophilins. In another article in which a closely related compound was tested against enterovirus 71 (ref. 4), *in vitro* resistance selection experiments identified one amino acid substitution selected after several passages with increasing concentrations of the compound. The substitution conferred a modest fivefold reduction in EV71 susceptibility, but no resistance to CsA, further challenging the hypothesis of a common mechanism of inhibition for the two molecules. Altogether, these results cast doubt as to the reality of the anti-cyclophilin properties of this class of molecules. Other families of compounds have been reported to inhibit cyclophilin activity *in vitro*, but they need to be externally validated and their potential as broad-spectrum antiviral compounds has not been evaluated^38^,^39^,^40^. These results emphasize the difficulty of developing efficacious small-molecule cyclophilin inhibitors unrelated to CsA or SfA and underline the originality and importance of our work.

Cyclophilins represent interesting targets for broad-spectrum antiviral drugs. Indeed, several cyclophilins, principally but not only CypA, have been convincingly shown to play a pivotal role in the life cycle of a number of viral families^3^. Our results showing inhibition of HCV, HIV and HCoV-229E coronavirus by compound **31** and related compounds, together with preliminary data suggesting hepatitis B virus replication inhibition, support the future development of our new family of cyclophilin inhibitors as broad-spectrum antiviral compounds with a high barrier to resistance. The high barrier to resistance, demonstrated with CsA derivatives in HCV infection^16^, results of the fact that cyclophilin inhibitors do not directly target a viral function but instead target a host protein involved at a key step of the viral life cycle. Thus, the likelihood to select viruses that are resistant to the action of the drug is low and, if such viruses were selected, they would be unlikely to yield high levels of resistance, as already shown^27^. This was confirmed here by the demonstration that amino acid substitutions in the HCV NS5A protein known to modestly reduce CsA- and CsA-derived inhibitor susceptibility of HCV also very modestly affected HCV susceptibility to the new compounds.

Other experiments showed that compound **31** inhibits mPTP opening as a result of CypD inhibition (Panel M, Ahmed-Belkacem A, *et al*., manuscript in preparation). This suggests that the new family of cyclophilin inhibitors also has the potential for medical utility in a number of pathological conditions in which mPTP opening has been shown to be involved, such as protection against cardiac ischaemia-reperfusion injury, protection against hepatic ischaemia-reperfusion injury in the context of liver transplantation or neurocellular protection in the context of Alzheimer's disease.

In conclusion, we used FBDD combined with a linking strategy and structure-based compound optimization, to generate a new family of non-peptidic, small-molecule cyclophilin inhibitors, unrelated to CsA or SfA, with potent PPIase inhibitory activity, antiviral effectiveness against HCV, HIV and coronaviruses *in vitro*, and druggable properties. This family of compounds has the potential to be useful in the high-barrier-to-resistance treatment of viral infections that use cyclophilins in their life cycle, as well as in various medical applications of CypD/mPTP opening-related cellular protection.

## Methods

### Expression and purification of CypD K133I

Mutant K133I of human CypD was expressed in *Escherichia coli* strain BL21(DE3). Bacteria were grown in Luria Broth medium at 37 °C up to an optical density (OD) of 0.6 at 600 nm and induced for 2 h with isopropyl-*β*-D-thiogalactopyranoside. Cells were lysed by sonication in buffer A, composed of 50 mM Tris at pH 7.5, 2 mM EDTA and 2 mM *β*-mercaptoethanol. Then, the cell lysate was clarified by centrifugation at 40,000 *g* for 30 min and the supernatant was loaded on Q-Sepharose and S-Sepharose columns in series equilibrated with buffer A. The S-Sepharose column was washed with equilibrium buffer and bound proteins were eluted with a linear gradient from 0 to 1 M NaCl. The combined peak fractions were loaded on an S75 column equilibrated with 20 mM Tris at pH 7.5, 200 mM NaCl, 2 mM EDTA and 1 mM dithiothreitol (DTT). This two-step purification protocol was sufficient to yield a pure protein.

^15^N labelling for the NMR experiments was obtained by growing the bacteria in M9 medium with ^15^N-labelled ammonium chloride as sole nitrogen source. The labelled CypD was then purified as described above.

### Expression and purification of CypA and CypB

CypA and CypB proteins carrying a hexahistidine tag (His-Tag) at their carboxy terminus were expressed in *E. coli* and purified. Briefly, cultures of C41(DE3) cells were grown at 37 °C for 1 h until the culture reached an OD of 0.6 at 600 nm and then induced with 1 mM isopropyl-*β*-D-thiogalactopyranoside for 4 h at 37 °C (CypA) or overnight at 22 °C (CypB). Cell pellets were resuspended in a lysis buffer 20 mM NaH_2_PO_4_ pH 7.8, 300 mM NaCl, 7 mM β-mercaptoethanol, 1 mg ml^−1^ lysozyme, 0.1 U μl^−1^ desoxyribonuclease and complete protease inhibitor tablets (Roche Diagnostics Corporation, Indianapolis, Indiana). The sonicated cell lysates were clarified by centrifugation at 10,000 *g* for 45 min at 4 °C, chromatographed on a Ni-NTA column and washed with a buffer containing 20 mM NaH_2_PO_4_ pH 7.8, 300 mM NaCl, 50 mM imidazole, 7 mM β-mercaptoethanol and 10% glycerol. The bound protein was eluted in 1 ml fractions with a buffer composed of 20 mM NaH_2_PO_4_ pH 7.8, 300 mM NaCl, 250 mM imidazole, 7 mM β-mercaptoethanol and 10% glycerol, monitored by the Bradford colorimetric assay. The purity of each cyclophilin was determined by Coomassie-stained SDS–PAGE analysis. Fractions enriched in cyclophilin (>95% purity) were pooled and dialysed against a buffer containing 20 mM NaH_2_PO_4_ pH 7.8, 300 mM NaCl, 1 mM DTT, 1 mM EDTA and 10% glycerol.

### PPIase enzyme assay

Cyclophilin PPIase activity was measured at 20 °C by using the standard chymotrypsin-coupled assay. The assay buffer (25 mM Hepes and 100 mM NaCl pH 7.8) and the cyclophilin (1,900 nM stock solution) were pre-cooled to 4 °C. Then, 5 μl of 50 mg ml^−1^ chymotrypsin in 1 mM HCl was added. The reaction was initiated by adding 20 μl of 3.2 mM peptide substrate (Suc-Ala-Ala-Cis-Pro-Phe-pNA) in LiCl/TFE solution with rapid inversion. After a delay from the onset of mixing, the absorbance of p-nitroaniline was followed at 390 nm until the reaction was complete (1 min). The final concentration of LiCl in the assay was 20 mM and TFE was present at a concentration of 4% (v/v). Absorbance readings were collected every 1 s by a spectrophotometer. For inhibition assessment, 5 μl of the tested compound in dimethyl sulfoxide (DMSO) was added to the cyclophilin solution in the assay buffer. CsA was used as a positive control in all measurements. The percentage inhibition of cyclophilin PPIase activity was calculated from the slopes and the values obtained represent the mean±s.d. of at least two independent measurements.

### Fragment library design and docking

Fragments purchased from Acros Organics (Geel, Belgium), Sigma-Aldrich (Saint Louis, Missouri), Maybridge (Tintagel, UK) and Chembridge (San Diego, California) were filtered applying a one-dimensional filter (molecular weight <300 g mol^−1^) based on the ‘rule of three'^41^. This virtual library was docked using programme interface LEA3D based on docking programme FlexX^42^,^43^. The X-ray coordinates of CypD (PDB-ID 2BIT) were used to dock the fragments. The site for docking was defined to cover the entire active site including the catalytic site and the gatekeeper pocket. No water molecule was included in the binding site. After docking, the top 10% poses of the docking were manually inspected.

### Compound synthesis

Chemical reagents were obtained from Aldrich Chemical (Saint Louis, Missouri), Acros Organics, abcr GmBh (Karlsruhe, Germany), ACB Blocks (Toronto, Canada) and Chembridge, and were used without further purification. Compound synthesis is described in the Supplementary Methods and their associated comments.

### NMR experiments

NMR samples used for screening contained 100 μM ^15^N-labelled protein, in a buffer consisting of 50 mM KH_2_PO_4_ at pH 7.3. ^15^N-HSQC experiments were recorded at 298 K in the presence of 5% D_2_O on a Bruker Avance 500 MHz spectrometer equipped with a cryoprobe. Typical acquisition time was 75 min per experiment. Mixtures of ten fragments (cocktails) at an individual concentration of 10 mM were screened. For cocktails soluble in water no DMSO-*d*_*6*_ was used, whereas 20% DMSO-*d*_*6*_ was used to solubilize cocktails insoluble in water. The attribution of the ^15^N-HSQC spectrum for CypD K133I deposited in the Biological Magnetic Resonance Bank (BMRB entry 7310) was used to re-attribute the ^15^N-HSQC spectrum of the protein in the presence of 20% DMSO-*d*_*6*_. Fragment binding was detected by comparing the ^15^N-HSQC spectra in the presence and in the absence of the fragment mixtures. As the fragment cocktails caused significant perturbations in the ^15^N-HSQC spectrum for some residues of the active site, one ^15^N-HSQC spectrum of ^15^N-CypD K133I was recorded in the presence of each individual fragment. All NMR data were processed using Bruker software XwinNMR Version 3.0, and the analyses and comparisons were made with in-house software Cindy for fragment screening.

NMR experiments with compounds **22** and **27** were recorded at 25 °C on an Inova Agilent 600 MHz spectrometer equipped with a triple-resonance 1H, 13C and 15 N probe. ^1^H-^15^N-HSQC spectra were recorded with 50 μM protein, 200 μM compound **27** and 500 μM compound **22**. Resonance assignment on ligand binding was checked using three-dimensional nuclear Överhauser enhancement spectroscopy–^15^N-HSQC spectra with 150 ms mixing time.

^1^H and ^13^C NMR spectra of compounds **22** and **25-31** are shown in Supplementary Figs 10–25.

### Crystallization and structure determination of ligand-CypD

Crystals of apo CypD K133I suitable for ligand soaking were obtained by a procedure described by Schlatter *et al*.^44^. Briefly, CypD K133I was buffer exchanged into 50 mM KH_2_PO_4_ at pH 7.3, 100 mM NaCl, 1 mM DTT and 2 mM EDTA, and concentrated to 30 mg ml^−1^. Apo CypD K133I crystals were grown using the hanging drop vapour diffusion method at 20 °C. Drops were formed by mixing 1 μl of protein with an equal volume of mother liquor containing 25–30% (w/v) PEG 4000 and equilibrated over 500 μl of the same solution. Crystals formed in about one night. The X-ray screening process requires a large amount of reproducible crystals available for soaking. Thus, the crystallization procedure was optimized with the seeding method using the ‘Seed Bead' protocol (Hampton Research, Aliso Viejo, California).

For fragment soaking, saturated solutions of immersion oil of 1.250 centistokes (Sigma-Aldrich) or 0.1 M DMSO stock solutions were made. Crystals were added to the saturated immersion oil for 10 min to 1 day or 0.1 μl DMSO stock solution was added to the growing drop for 10 min to 1 h before flash freezing and data collection. Crystals soaked with a DMSO stock solution were cryoprotected for data collection by brief immersion in oil.

X-ray diffraction data were collected in-house with an X-Ray generator (RU-200, Rigaku, Tokyo, Japan) and the Image Plate Detector mar300 (MarResearch, Norderstedt, Germany) or at the European Synchrotron Radiation Facility in Grenoble, France, on beamlines BM30, ID14-1, ID14-2, ID14-3, ID14-4 and ID29. Data were integrated and processed using MOSFLM and SCALA of the CCP4 suite. The crystals belong to the space group P4_1_2_1_2 (*a*=*b*=57 Å and *c*=87 Å) with one monomer in the asymmetric unit. The structures were solved by molecular replacement using PDB entry 2BIT as the search model. Bound ligands were manually identified and fitted into *F*_o_*–F*_c_ electron density using Coot. The structures were refined by rounds of rebuilding in Coot and refinement using Refmac of the CCP4 suite. Data collection and refinement statistics for crystal structures are presented in Supplementary Tables 4 to 11.

### Crystallization and structure determination of ligand-CypA

Crystals of apo CypA suitable for ligand soaking were obtained by means of hanging drop vapour diffusion by mixing 1 μl of CypA 20 mg ml^−1^ in 50 mM Tris pH 7.8, 100 mM NaCl and 1 μl of Hepes 100 mM pH 7.5, 20% PEG 10k (w/v), 5% ethanol at 20 °C, equilibrated over 500 μl of the same solution. Crystals formed in about one night. A 0.1 μl DMSO stock solution of compound **22** was added to the growing drop for 10 min before flash freezing and data collection. X-ray diffraction data were collected in-house with the X-Ray generator RU-200 and the Image Plate Detector mar300. Data were integrated and processed using MOSFLM and SCALA of the CCP4 suite. The crystals belong to the space group P2_1_2_1_2_1_ (*a*=40.7 Å, *b*=52.3 Å and *c*=86.9 Å) with one monomer in the asymmetric unit. The structures were solved by molecular replacement using PDB entry 1CWA as the search model. Bound ligand was manually identified and fitted into *F*_o_*–F*_c_ electron density using Coot. The structures were refined by rounds of rebuilding in Coot and refinement using Refmac of the CCP4 suite. Data collection and refinement statistics for crystal structure are presented in Supplementary Table 8.

### Isothermal titration calorimetry

The proteins were dialysed against buffer A containing 10 mM Hepes pH 7.4 and 150 mM NaCl. Twenty to 50 μM of protein was loaded into the MicroCal VP-ITC (Malvern, Orsay, France) isothermal titration calorimeter cell (∼2 ml, cell volume ∼1.4 ml). The titration syringe (250 μl volume) was filled with 1 mM ligand solution in buffer A. Titrations were carried out using 40 injections of 4 μl each, injected at 5 min intervals. Stirring speed was 400 r.p.m. Titrations were carried out at a constant temperature of 25 °C. Data were fit to a single site binding model using Origin 5.0 software.

### Assessment of anti-HCV activity

An HCV genotype 1b bicistronic replicon was transfected in Huh7 cells^45^ grown in DMEM medium Glutamax II (Invitrogen, Carlsbad, California) supplemented with 10% fetal bovine serum, 50 IU ml^−1^ penicillin, 100 μg ml^−1^ streptomycin, 0.1 μg ml^−1^ fungizone and 600 μg ml geneticin (G418). HCV replicon-harbouring cells were seeded at a low density of 5,000 cells per well in 96-well plates. The cells were treated with increasing concentrations of the tested compounds in DMEM containing 10% fetal bovine serum and 1% DMSO without G418 and cultured for 3 days. Total RNA was extracted using the RNeasy 96 kit (Qiagen, Hilden, Germany). HCV RNA levels were measured by means of a quantitative real-time PCR assay using the Taqman technology with HCV-specific primers (sense 5′-CGCCCAAACCAGAATACGA-3′ and antisense 5′-AGATAGTACACCCTTTTGCCAGATG-3′) and probe (5′-6-FAM-CAATGTGTCAGTCGCG-TAMRA-3′) on an ABI 7003 device (Applied Biosystems, Foster City, California). HCV RNA levels were measured by means of a Nanodrop 1000 spectrophotometer (Nanodrop Technologies, Wilmington, Delaware). The results were normalized to the *GAPDH* gene. Each data point represents the average of at least three replicates in cell culture. HCV RNA level reductions after treatment were assessed by comparing the level of HCV RNA in compound-treated cells with that of control cells treated with 1% DMSO.

### Assessment of anti-HIV activity

Compounds diluted in DMSO (0.4 μl) was added to 40 μl of cell growth medium (RPMI 1640, 10% fetal bovine serum, 1% penicillin/streptomycin, 1% L-glutamine and 1% Hepes) in each well of 384-well assay plates (10 concentrations) in quadruplicate. One-millilitre aliquots of 2 × 10^6^ MT-4 cells were pre-infected for 1 and 3 h, respectively, at 37 °C with 25 μl of either cell growth medium or a fresh 1:250 dilution of an HIV-IIIb concentrated ABI stock (0.004 multiplicity of infection). Infected and uninfected cells were diluted in cell growth medium and 35 μl of a suspension of 2,000 cells was added to each well of the assay plates, respectively. The assay plates were then incubated in a 37 °C incubator. After 5 days of incubation, 25 μl of twofold concentrated CellTiter-Glo Reagent (Promega Biosciences, Madison, Wisconsin) was added to each well of the assay plates. Cell lysis was carried out by incubating at room temperature for 2–3 min and then chemiluminescence was read using an Envision multimode plate reader (PerkinElmer, Waltham, Massachusetts). Data were converted into percentages of the untreated control and non-linear regression was performed to calculate EC_50_ values. For compound cytotoxicity assessment, the protocol was identical except that uninfected cells were used.

### Assessment of anti-human coronavirus 229E activity

MRC-5 cells (RD-Biotech, Besançon, France) were cultured in DMEM containing 10% FCS, 50 U ml^−1^ penicillin, 50 μg ml^−1^ streptomycin and 0.1 μg ml^−1^ amphotericin B (Fungizone, Life Technologies, Carlsbad, California). Twelve hours before infection, MRC-5 cells were seeded at the density of 2 × 10^4^ cells per well in a 96-well plate. Cells were infected with human coronavirus 229E at the multiplicity of infection of 1 in DMEM containing 2% FCS, 50 U ml^−1^ penicillin, 50 μg ml^−1^ streptomycin and 0.1 μg ml^−1^ amphotericin B for 72 h in the presence of increasing concentrations of inhibitors. The viral cytopathic effect was quantified with ‘Cytotox-Glo Cytotoxicity Assay' (Promega, Madison, Wisconsin). Virus-induced cytotoxicity was calculated as relative light unit signal in infected treated cells minus relative light unit signal in non-infected treated cells.

### Calcineurin phosphatase assay

The inhibition of calcineurin phosphatase activity was measured by measuring the dephosphorylation of *p*-nitrophenyl phosphate (R&D Systems, Minneapolis, USA), according to the manufacturer's instructions. Briefly, Csa or compound **31** were incubated in a 1:1 molar ratio with recombinant CypA at room temperature for 30 min, in the presence of 1.32 nM of calcineurin and 50 nM of calmodulin. After *p*-nitrophenyl phosphate addition, the reaction was run for 35 min at 37 °C. The reaction was terminated by addition of BIOMOL green reagent and OD was measured at 630 nm. The results are the means of three independent experiments performed in duplicate. They are presented as the percentage of calcineurin activity relative to that in the inhibitor-free control.

### Inhibition of IL-2 production in Jurkat cells

Jurkat cells (2 × 10^5^ cells per well) were pretreated for 1 h at 37 °C with either compound **31** at concentrations ranging from 0.1 to 20 μM or CsA at concentrations ranging from 0.01 to 2 μM. Duplicate evaluations were included for each test concentration. At the end of the pretreatment period, cells were induced with a mixture of phorbol myristate acetate and ionomycin at 50 ng ml^−1^ and 1.34 μM, respectively. After a 6 h incubation, sample supernatants were collected for measurement of IL-2 production using a commercial kit (Quantikine ELISA, R&D Systems).

### LogD determination

The octanol/water partitioning (LogD) values of compounds were determined from their retention times during reverse-phase HPLC at pH 7.4 with diode array ultraviolet/visible detection. The LogD values were obtained by interpolation within a standard curve (range LogD: 0.3–5.7) generated with seven standards, with LogD values previously determined using the shake-flask method.

### Solubility and chemical stability

Kinetic solubility was determined by diluting a DMSO stock of the compound into the test media solutions (PBS pH 7.4 and 0.1 N HCl pH 1.0) to a final concentration of 100 μM with a total DMSO concentration of 1% (v/v) at 37 °C. The solutions were incubated at room temperature with shaking for 24 h and were then centrifuged, and the recovered supernatants assayed by HPLC with diode array ultraviolet/visible detection. Solubility values were calculated by comparing the amount (by chromatographic peak area) of compound detected in the defined test solution with an unextracted standard. Relative chemical stabilities of compounds after 24 h incubation in PBS at 37 °C were also determined. The solubility and chemical stability of a quality-control compound (amprenavir) were determined in parallel.

### Caco-2 cell permeability

Permeability and efflux potential were determined using confluent (≥21 day) monolayers of Caco-2 cells grown on transwell filters. Compounds were added to donor wells at a target concentration of 10 μM and rates of appearance in receiver wells (containing 1% BSA to maintain sink conditions) were determined by liquid chromatography coupled to tandem mass spectrometry (LC-MS/MS). Quality controls included transepithelial electrical resistance, lucifer yellow exclusion, low (atenolol) and high (propranolol) permeability controls and the ability to demonstrate polarized efflux of digoxin.

### Protein binding

The extent of binding to pooled human plasma and to cell culture medium containing 10% fetal bovine serum was determined by equilibrium dialysis against isotonic phosphate buffer. Equilibrium dialysis was conducted at 37 °C with initial concentrations of compounds of 2 μM in the non-buffer matrix. A 3 h dialysis time was used for equilibration. Following dialysis, plasma samples were drained into pre-weighed polypropylene tubes containing buffer and buffer samples were drained into pre-weighed tubes containing blank plasma. Post-dialysis plasma and buffer weights were measured and recorded for concentration and recovery calculations. After precipitation and centrifugation, LC-MS/MS assays were used for the analysis of each of the protein/phosphate buffer-mixed matrices. Relative binding to human plasma and cell culture medium was determined in a similar manner, except that the two matrices were dialysed against each other directly and the compound was spiked into both matrices. After equilibration, the plasma sample was diluted with blank cell culture medium and the cell culture medium diluted with blank plasma.

### Stability with hepatic microsomal fractions

The compounds (3 μM) were incubated in duplicate for up to 1 h in the presence of human hepatic microsomal fractions (0.5 mg protein ml^−1^ final) at 37 °C. The addition of cofactor solution (NADPH generating system) initiated the reaction and aliquots were removed at 0, 5, 15, 30, 45 and 60 min after the start. The concentrations of the compounds in each sample were determined using specific LC-MS/MS assays. The half-life for the disappearance of each was determined by fitting the concentration-time data with a monophasic exponential model. A control compound (verapamil) was run in parallel to test the enzymatic integrity of the microsomal fractions.

### Metabolism by recombinant human cytochrome P450

Five micromoles of compound was incubated with individual bacterially expressed recombinant human cytochromes P450 co-expressed with human NADPH cytochrome P450 reductase. The reduction in substrate concentration was monitored over 45 min using specific LC-MS/MS assays and the *in vitro* half-life calculated in the same manner as that described for hepatic microsomal stability. Enzyme-selective positive control substrates were tested in parallel.

### Cytochrome P450 enzyme inhibition assays

Up to 25 μM of compounds **29** and **30** was incubated with human hepatic microsomal fractions and NADPH in the presence of individual probe substrates. All assays were designed so that conditions were linear with respect to time and protein concentration. Substrates were present at concentrations equal to or lower than their respective Km values. Enzyme selective products were determined by fluorometry (ethoxyresorufin *O*-deethylase) or by specific LC-MS/MS assays. Relative enzyme activities were determined by comparison with those assayed with DMSO vehicle instead of inhibitor. IC_50_ values were calculated by nonlinear curve fitting using a sigmoidal model. Positive control inhibitors for each enzyme were tested in parallel.

### Data availability

Data supporting the findings of this study are available within the article and its Supplementary Information files and from the corresponding authors upon reasonable request. PDB: the complex structures for compounds **5**, **6**, **9**, **11** to **24**, **26** to **29** with CypD K133I mutant enzyme are deposited under accession codes 3RCI, 3R59, 3RCF, 3RCG, 3R4G, 3R54, 3RD9, 3R49, 3R56, 3RCL, 3RDB, 3RCK, 3RDA, 3R57, 3RDC, 4J58, 4J5E, 4J5D, 4J5B, 4J59 and 4J5C, respectively. The complex structure for compound **22** with CypA is deposited under accession code 3RDD. Stereo views of cocrystal structures of the compounds with cyclophilin D are available at https://figshare.com/articles/Stereo_views_of_cocrystal_structures_of_cyclophilin_inhibitors_with_cyclophilin_D/3490493.

## Additional information

**How to cite this article**: Ahmed-Belkacem, A. *et al*. Fragment-based discovery of a new family of non-peptidic small-molecule cyclophilin inhibitors with potent antiviral activities. *Nat. Commun.* 7:12777 doi: 10.1038/ncomms12777 (2016).

## Supplementary Material

Supplementary Information Supplementary figures 1-25, Supplementary tables 1-11 and Supplementary Methods.

## Figures and Tables

**Figure 1:**
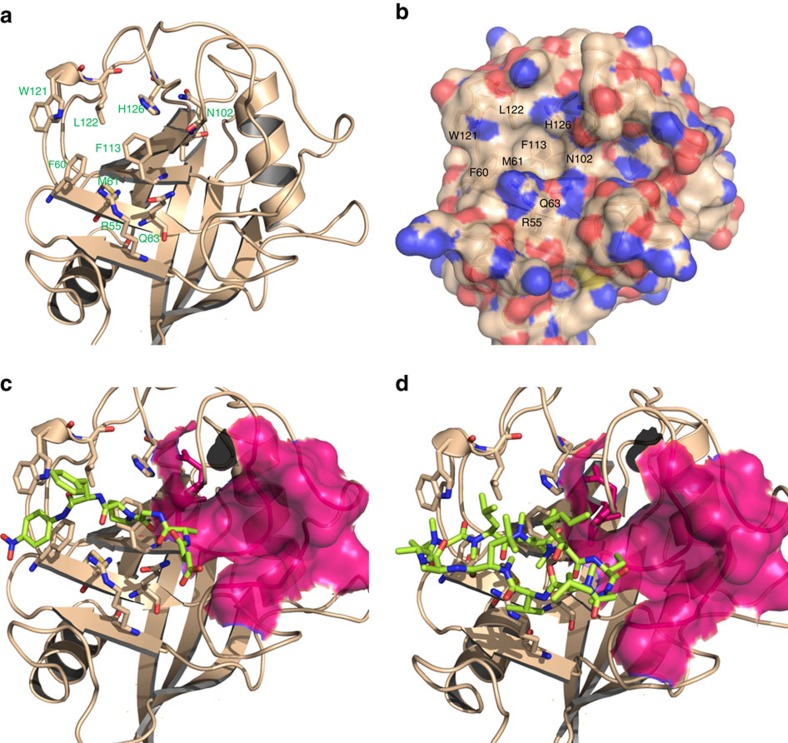
Crystal structure of CypA alone or in complex. (**a**) Cartoon representation of CypA, with the catalytic residues in stick format. (**b**) Surface representation of CypA showing the catalytic pocket (left) and the gatekeeper pocket (right). (**c**) Cartoon representation of PDB 1ZKF showing the catalytic residues and the succinyl-AGPF-pNA substrate (green) in stick format, and the gatekeeper pocket in surface representation (pink). (**d**) Cartoon representation of PDB 1CWA showing the catalytic residues and the cyclophilin inhibitor CsA (green) in stick format, and the gatekeeper pocket in surface representation (pink).

**Table 1:** *In vitro* inhibitory activities and LE of the two selected fragments and of the compounds resulting from their linkage and subsequent chemical optimization in CypA, CypB and CypD PPIase assays.

**Compound**	**CypA IC**_**50**_ **(μM)**	**CypB IC**_**50**_ **(μM)**	**CypD IC**_**50**_ **(μM)**	**LE (kcal per heavy atom)**
CsA	0.01±0.003	0.01±0.005	0.02±0.003	ND
Alisporivir	0.07±0.003	0.04±0.012	0.03±0.005	ND
13	>5,000	>5,000	>5,000	<0.45
6	>5,000	>5,000	>5,000	<0.31
22	13.1±5.9	6.1±3.8	6.2±3.7	0.36
23	>500	>500	>500	<0.29
24	2.8±0.6	1.2±0.1	11.4±3.0	0.27
25	3.4±0.7	3.7±1.4	6.2±2.3	0.28
26	0.6±0.2	0.8±0.1	1.1±0.2	0.31
27	0.4±0.1	0.6±0.1	0.6±0.1	0.31
28	1.5±0.5	1.8±0.9	1.4±0.2	0.26
29	0.8±0.1	0.5±0.2	0.7±0.2	0.26
30	3.3±1.4	1.9±1.5	3.0±0.7	0.23
31	0.1±0.07	0.08±0.04	0.2±0.08	0.28
32	7.4±6.8	8.7±1.5	12.8±2.5	0.34
33	4.2±1.6	2.2±1.2	7.7±0.8	0.22

CsA, cyclosporine A; CypA, cyclophilin A; CypB, cyclophilin B; CypD, cyclophilin D; LE, ligand efficiency; ND, not defined; PPIase, peptidyl-prolyl *cis/trans* isomerase.

CsA and the CsA analogue alisporivir are used as references.

**Table 2:** *In vitro* antiviral activities of the cyclophilin inhibitors against HCV, HIV and HCoV-229E, and cellular toxicities in their respective cellular models.

**Compound**	**Huh7 cells**		**MT4 cells**		**MRC5 cells**	
	**HCV genotype 1b replicon EC**_**50**_ **(μM)**	**HCV genotype 1b replicon CC**_**50**_ **(μM)**	**HIV-IIIb in MT4 cells EC**_**50**_ **(μM)**	**MT4 cells CC**_**50**_ **(μM)**	**HCoV-229E EC**_**50**_ **(μM)**	**MRC5 cells CC**_**50**_ **(μM)**
Csa	0.3±0.1	19.2±4.5	>CC_50_	7.5±1.7	>CC_50_	9.3±1.7
Alisporivir	0.01±0.0007	32.3±22.0	NT	NT	2.6±0.6	9.7±2.2
26	6.0±0.7	>100	3.6±0.8	>53	66.3±24.0	>100
27	2.7±2.5	>100	6.8±2.3	>53	34.6±18.3	>100
29	1.7±1.2	>100	13.0±2.7	40±5.4	27.6±8.6	>100
30	1.4±1.2	>100	15.0±1.2	>53	7.2±1.8	>100
31	0.4±0.3	>100	NT	NT	44.7±2.2	>100
32	8.0±1.3	>100	NT	NT	71.5±4.3	>100
33	8.4±1.0	>100	NT	NT	55.3±12.2	>100

CsA, cyclosporine A; HCV, hepatitis C virus; HCoV-229E, human coronavirus 229E; HIV, human immunodeficiency virus; NT, not tested.

CsA and the CsA analogue alisporivir are used as references.

**Table 3:** Effect of the D320E and R318H HCV NS5A protein substitutions on the antiviral effect of the cyclophilin inhibitors.

**Compound**	**EC**_**50**_ **(μM) HCV (WT) subgenomic replicon**	**HCV (D320E) subgenomic replicon**		**HCV (R318H) subgenomic replicon**	
		**EC**_**50**_ **(μM)**	**EC**_**50**_ **fold-increase as compared with WT**	**EC**_**50**_ **(μM)**	**EC**_**50**_ **fold-increase as compared with WT**
CsA	0.1±0.02	0.5±0.06	3.7	0.4±0.03	2.9
Alisporivir	0.04±0.0005	0.1±0.003	2.5	0.08±0.02	2.0
27	5.6±0.7	10.3±2.7	1.8	4.8±0.5	0.9
29	2.9±0.04	3.9±0.4	1.3	3.5±0.6	1.2
30	2.1±0.09	5.8±1.1	2.8	3.2±1.2	1.5
31	0.3±0.16	0.3±0.1	1.0	0.4±0.01	1.4

CsA, cyclosporine A; HCV, hepatitis C virus; WT, wild type.

CsA and its analogue alisporivir were used as references.

## References

[b1] De Clercq E. Antivirals: past, present and future. Biochem. Pharmacol. 85, 727–744 (2013). 23270991 10.1016/j.bcp.2012.12.011

[b2] Zhu J. D., Meng W., Wang X. J. & Wang H. C. Broad-spectrum antiviral agents. Front. Microbiol. 6, 517 (2015). 26052325 10.3389/fmicb.2015.00517 PMC4440912

[b3] Hopkins S. & Gallay P. A. The role of immunophilins in viral infection. Biochim. Biophys. Acta 1850, 2103–2110 (2014). 25445708 10.1016/j.bbagen.2014.11.011 PMC4491039

[b4] Qing J. . Cyclophilin A associates with enterovirus-71 virus capsid and plays an essential role in viral infection as an uncoating regulator. PLoS Pathog. 10, e1004422 (2014). 25275585 10.1371/journal.ppat.1004422 PMC4183573

[b5] Nigro P., Pompilio G. & Capogrossi M. C. Cyclophilin A: a key player for human disease. Cell Death Dis. 4, e888 (2013). 24176846 10.1038/cddis.2013.410 PMC3920964

[b6] Baines C. P. . Loss of cyclophilin D reveals a critical role for mitochondrial permeability transition in cell death. Nature 434, 658–662 (2005). 15800627 10.1038/nature03434

[b7] Elrod J. W. & Molkentin J. D. Physiologic functions of cyclophilin D and the mitochondrial permeability transition pore. Circ. J. 77, 1111–1122 (2013). 23538482 10.1253/circj.cj-13-0321 PMC6397958

[b8] Javadov S. & Kuznetsov A. Mitochondrial permeability transition and cell death: the role of cyclophilin D. Front. Physiol. 4, 76 (2013). 23596421 10.3389/fphys.2013.00076 PMC3622878

[b9] Rasola A. & Bernardi P. Mitochondrial permeability transition in Ca(2+)-dependent apoptosis and necrosis. Cell Calcium 50, 222–233 (2011). 21601280 10.1016/j.ceca.2011.04.007

[b10] Davis T.L. . Structural and biochemical characterization of the human cyclophilin family of peptidyl-prolyl isomerases. PLoS Biol. 8, e1000439 (2010). 20676357 10.1371/journal.pbio.1000439 PMC2911226

[b11] Wang P. & Heitman J. The cyclophilins. Genome Biol. 6, 226 (2005). 15998457 10.1186/gb-2005-6-7-226 PMC1175980

[b12] Carbajo-Lozoya J. . Human coronavirus NL63 replication is cyclophilin A-dependent and inhibited by non-immunosuppressive cyclosporine A-derivatives including alisporivir. Virus Res. 184, 44–53 (2014). 24566223 10.1016/j.virusres.2014.02.010 PMC7114444

[b13] Madan V., Paul D., Lohmann V. & Bartenschlager R. Inhibition of HCV replication by cyclophilin antagonists is linked to replication fitness and occurs by inhibition of membranous web formation. Gastroenterology 146, 1361–1372 (2014). 24486951 10.1053/j.gastro.2014.01.055

[b14] Pfefferle S. . The SARS-coronavirus-host interactome: identification of cyclophilins as target for pan-coronavirus inhibitors. PLoS Pathog. 7, e1002331 (2011). 22046132 10.1371/journal.ppat.1002331 PMC3203193

[b15] Flisiak R. . The cyclophilin inhibitor Debio-025 combined with PEG-IFN alpha-2a significantly reduces viral load in treatment-naïve hepatitis C patients. Hepatology 49, 1460–1468 (2009). 19353740 10.1002/hep.22835

[b16] Pawlotsky J. M. . Alisporivir plus ribavirin, interferon-free or in combination with peg-interferon, for HCV genotype 2 or 3 infection. Hepatology 62, 1013–1023 (2015). 26118427 10.1002/hep.27960

[b17] Akool el S. . Molecular mechanisms of TGF beta receptor-triggered signaling cascades rapidly induced by the calcineurin inhibitors cyclosporin A and FK506. J. Immunol. 181, 2831–2845 (2008). 18684975 10.4049/jimmunol.181.4.2831

[b18] Esser-Nobis K. . The cyclophilin-inhibitor alisporivir stimulates antigen presentation thereby promoting antigen-specific CD8-T-cell activation. J. Hepatol. 64, 1305–1314 (2016). 26921685 10.1016/j.jhep.2016.02.027 PMC7172366

[b19] Matsuda S. . Two distinct action mechanisms of immunophilin-ligand complexes for the blockade of T-cell activation. EMBO Rep. 1, 428–434 (2000). 11258483 10.1093/embo-reports/kvd090 PMC1083763

[b20] Morjani H. & Madoulet C. Immunosuppressors as multidrug resistance reversal agents. Methods Mol. Biol. 596, 433–446 (2010). 19949935 10.1007/978-1-60761-416-6_19

[b21] Bing P., Maode L., Li F. & Sheng H. Comparison of expression of TGF-beta 1, its receptors TGF-beta 1R-I and TGF-beta 1R-II in rat kidneys during chronic nephropathy induced by cyclosporine and tacrolimus. Transplant Proc. 38, 2180–2182 (2006). 16980036 10.1016/j.transproceed.2006.06.102

[b22] Chi J. . Cyclosporin A induces apoptosis in H9c2 cardiomyoblast cells through calcium-sensing receptor-mediated activation of the ERK MAPK and p38 MAPK pathways. Mol. Cell Biochem. 367, 227–236 (2012). 22678567 10.1007/s11010-012-1336-5

[b23] Erlanson D. A. Introduction to fragment-based drug discovery. Top. Curr. Chem. 317, 1–32 (2012). 21695633 10.1007/128_2011_180

[b24] Hajduk P. J. & Greer J. A decade of fragment-based drug design: strategic advances and lessons learned. Nat. Rev. Drug Discov. 6, 211–219 (2007). 17290284 10.1038/nrd2220

[b25] Wolkenstein P. . A slow acetylator genotype is a risk factor for sulphonamide-induced toxic epidermal necrolysis and Stevens-Johnson syndrome. Pharmacogenetics 5, 255–258 (1995). 8528274 10.1097/00008571-199508000-00011

[b26] Woosley R. L. . Effect of acetylator phenotype on the rate at which procainamide induces antinuclear antibodies and the lupus syndrome. N. Engl. J. Med. 298, 1157–1159 (1978). 306574 10.1056/NEJM197805252982101

[b27] Coelmont L. . Debio-025, a cyclophilin binding molecule, is highly efficient in clearing hepatitis C virus (HCV) replicon-containing cells when used alone or in combination with specifically targeted antiviral therapy for HCV (STAT-C) inhibitors. Antimicrob. Agents Chemother. 53, 967–976 (2009). 19104013 10.1128/AAC.00939-08 PMC2650540

[b28] Fruman D. A., Klee C. B., Bierer B. E. & Burakoff S. J. Calcineurin phosphatase activity in T lymphocytes is inhibited by FK-506 and cyclosporin A. Proc. Natl Acad. Sci. USA 89, 3686–3690 (1992). 1373887 10.1073/pnas.89.9.3686 PMC525555

[b29] Murray C. W. & Rees D. C. The rise of fragment-based drug discovery. Nat. Chem. 1, 187–192 (2009). 21378847 10.1038/nchem.217

[b30] Baker M. Fragment-based lead discovery grows up. Nat. Rev. Drug Discov. 12, 5–7 (2013). 23274457 10.1038/nrd3926

[b31] Eitner K. & Koch U. From fragment screening to potent binders: strategies for fragment-to-lead evolution. Mini Rev. Med. Chem. 9, 956–961 (2009). 19601891 10.2174/138955709788681645

[b32] Howard B. R., Vajdos F. F., Li S., Sundquist W. I. & Hill C. P. Structural insights into the catalytic mechanism of cyclophilin A. Nat. Struct. Biol. 10, 475–481 (2003). 12730686 10.1038/nsb927

[b33] Sweeney Z. K., Fu J. & Wiedmann B. From chemical tools to clinical medicines: nonimmunosuppressive cyclophilin inhibitors derived from the cyclosporin and sanglifehrin scaffolds. J. Med. Chem. 57, 7145–7159 (2014). 24831536 10.1021/jm500223x

[b34] Chung S., Parker J. B., Bianchet M., Amzel L. M. & Stivers J. T. Impact of linker strain and flexibility in the design of a fragment-based inhibitor. Nat. Chem. Biol. 5, 407–413 (2009). 19396178 10.1038/nchembio.163 PMC3178264

[b35] Guichou J. F. . Structure-based design, synthesis, and biological evaluation of novel inhibitors of human cyclophilin. J. Med. Chem. 49, 900–910 (2006). 16451056 10.1021/jm050716a

[b36] Li J. . Discovery of dual inhibitors targeting both HIV-1 capsid and human cyclophilin A to inhibit the assembly and uncoating of the viral capsid. Bioorg. Med. Chem. 17, 3177–3188 (2009). 19328002 10.1016/j.bmc.2009.02.051

[b37] Ni S. . Discovering potent small molecule inhibitors of cyclophilin A using *de novo* drug design approach. J. Med. Chem. 52, 5295–5298 (2009). 19691347 10.1021/jm9008295

[b38] Daum S. . Isoform-specific inhibition of cyclophilins. Biochemistry 48, 6268–6277 (2009). 19480458 10.1021/bi9007287 PMC2753677

[b39] Dunsmore C. J. . Design and synthesis of conformationally constrained cyclophilin inhibitors showing a cyclosporin-A phenotype in *C. elegans*. Chembiochem 12, 802–810 (2011). 21337480 10.1002/cbic.201000413

[b40] Yang S. . Structure-based discovery of novel cyclophilin A inhibitors for the treatment of hepatitis C virus infections. J. Med. Chem. 58, 9546–9561 (2015). 26613291 10.1021/acs.jmedchem.5b01064

[b41] Jhoti H., Williams G., Rees D. C. & Murray C. W. The ‘rule of three' for fragment-based drug discovery: where are we now? Nat. Rev. Drug Discov. 12, 644–645 (2013). 23845999 10.1038/nrd3926-c1

[b42] Douguet D., Munier-Lehmann H., Labesse G. & Pochet S. LEA3D: a computer-aided ligand design for structure-based drug design. J. Med. Chem. 48, 2457–2468 (2005). 15801836 10.1021/jm0492296

[b43] Kramer B., Rarey M. & Lengauer T. Evaluation of the FLEXX incremental construction algorithm for protein-ligand docking. Proteins 37, 228–241 (1999). 10584068 10.1002/(sici)1097-0134(19991101)37:2<228::aid-prot8>3.0.co;2-8

[b44] Schlatter D. . Crystal engineering yields crystals of cyclophilin D diffracting to 1.7A resolution. Acta Crystallogr. D Biol. Crystallogr. 61, 513–519 (2005). 15858260 10.1107/S0907444905003070

[b45] Krieger N., Lohmann V. & Bartenschlager R. Enhancement of hepatitis C virus RNA replication by cell culture-adaptive mutations. J. Virol. 75, 4614–4624 (2001). 11312331 10.1128/JVI.75.10.4614-4624.2001 PMC114214

