# Supplementary material for: Fragment-based discovery of a new family of non-peptidic small-molecule cyclophilin inhibitors with potent antiviral activities
Source: Nat Commun. 2016 Sep 22;7:12777. doi: 10.1038/ncomms12777 (PMC5036131; doi:10.1038/ncomms12777)
Supplement: Supplementary Information — Supplementary figures 1-25, Supplementary tables 1-11 and Supplementary Methods. [file ncomms12777-s1.pdf]

Supplementary Figures

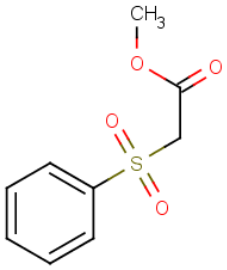

1
%I=40% at 1 mM
LE <0.29

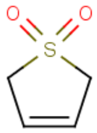

2
%I=2% at 1 mM
LE n.d.

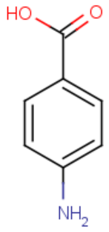

3
%I=32% at 1 mM
LE <0.42

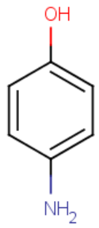

4
%I=1% at 1 mM
LE n.d.

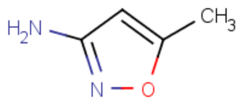

5
%I=10% at 1 mM
LE n.d.

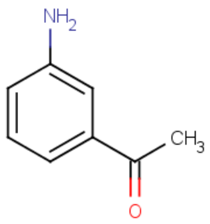

6
%I=17% at 1 mM
LE <0.46

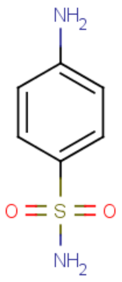

7
%I=32% at 1 mM
LE <0.37

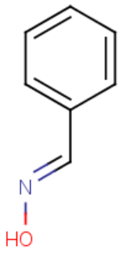

8
%I=32% at 1 mM
LE <0.46

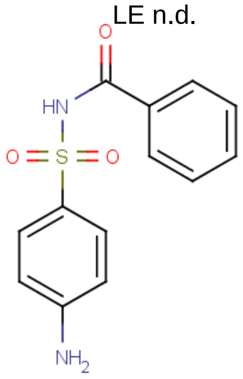

9
%I=39% at 1 mM
LE <0.24

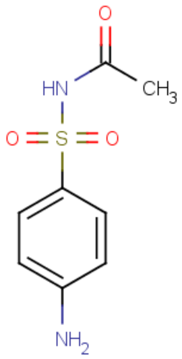

10
%I=21% at 1 mM
LE <0.29

Supplementary Figure 1. Chemical structure of NMR fragment hits. The chemical structures of the 10 initial fragment hits are shown with their % CypD inhibition (%I) at 1 mM and ligand efficiency (LE).

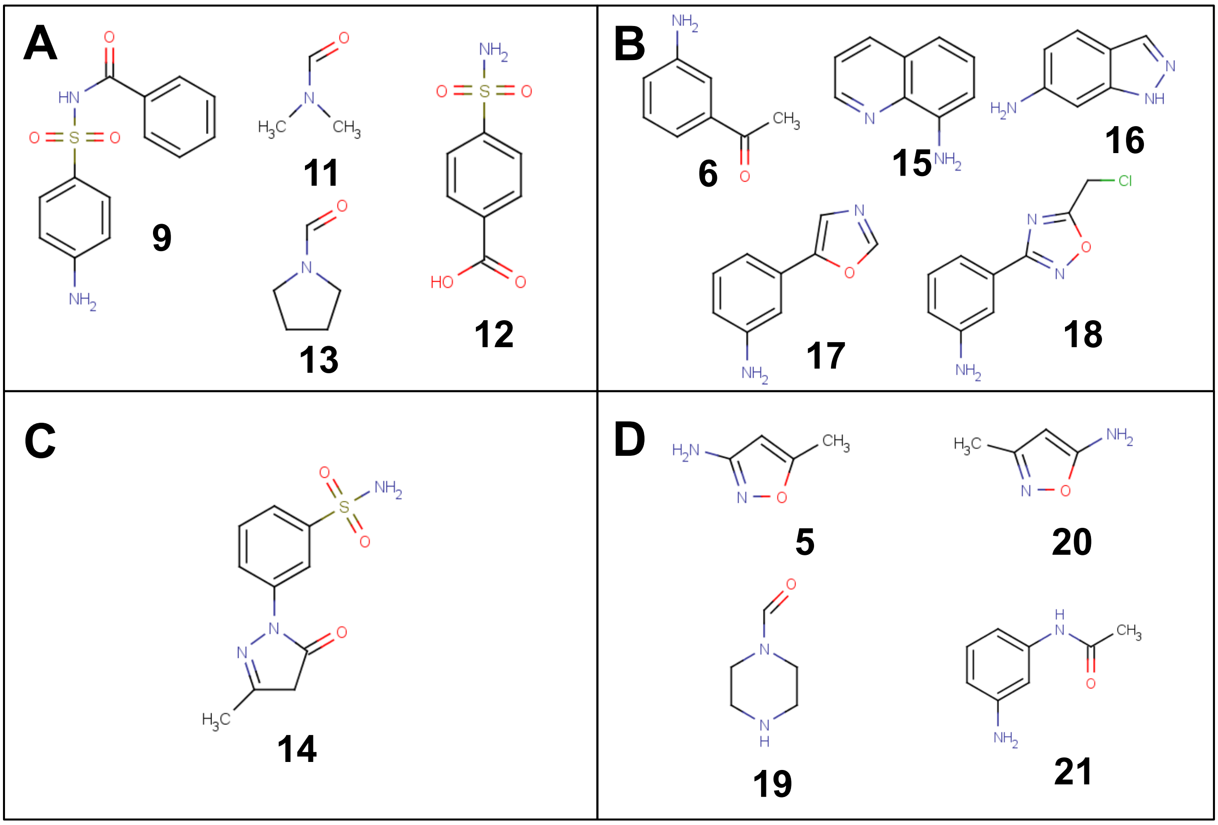

Supplementary Figure 2. Chemical structure of the 14 fragment hits. (A) Fragments binding the CypD catalytic site. (B) Fragments binding the gatekeeper pocket of CypD. (C) Fragments binding between the catalytic site and the gatekeeper pocket. (D) Multibinder fragments.

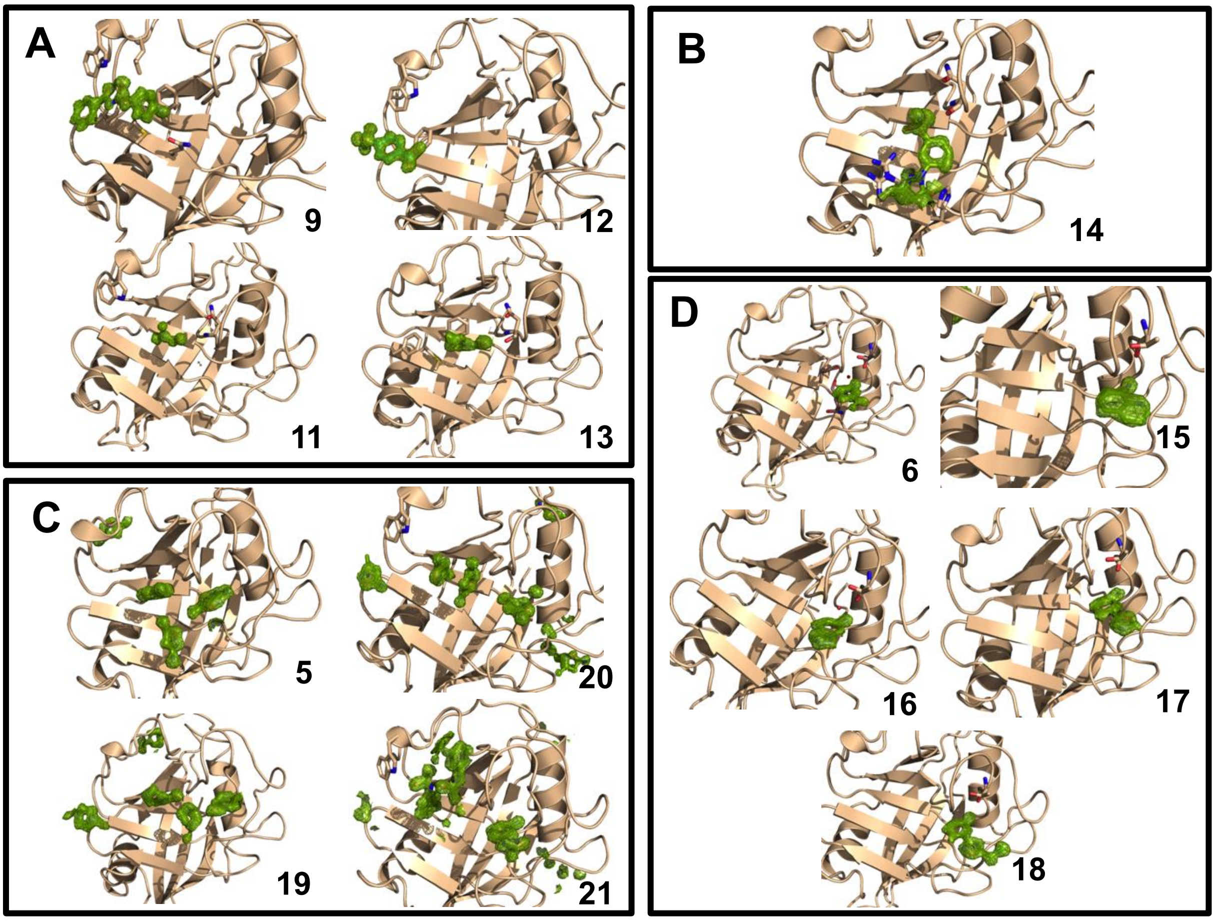

Supplementary Figure 3. Cocrystal structures of the fragment hits with CypD. CypD in represented in cartoon format and the fragments in stick format. The green mesh and surface represent the electron density maps ($2F_o - F_c$ omit map contoured at 1.0 σ). (A) Fragment hits binding the catalytic site. (B) Fragment hits binding between the two sites. (C) Multibinder fragment hits. (D) Fragment hits binding the gatekeeper pocket.

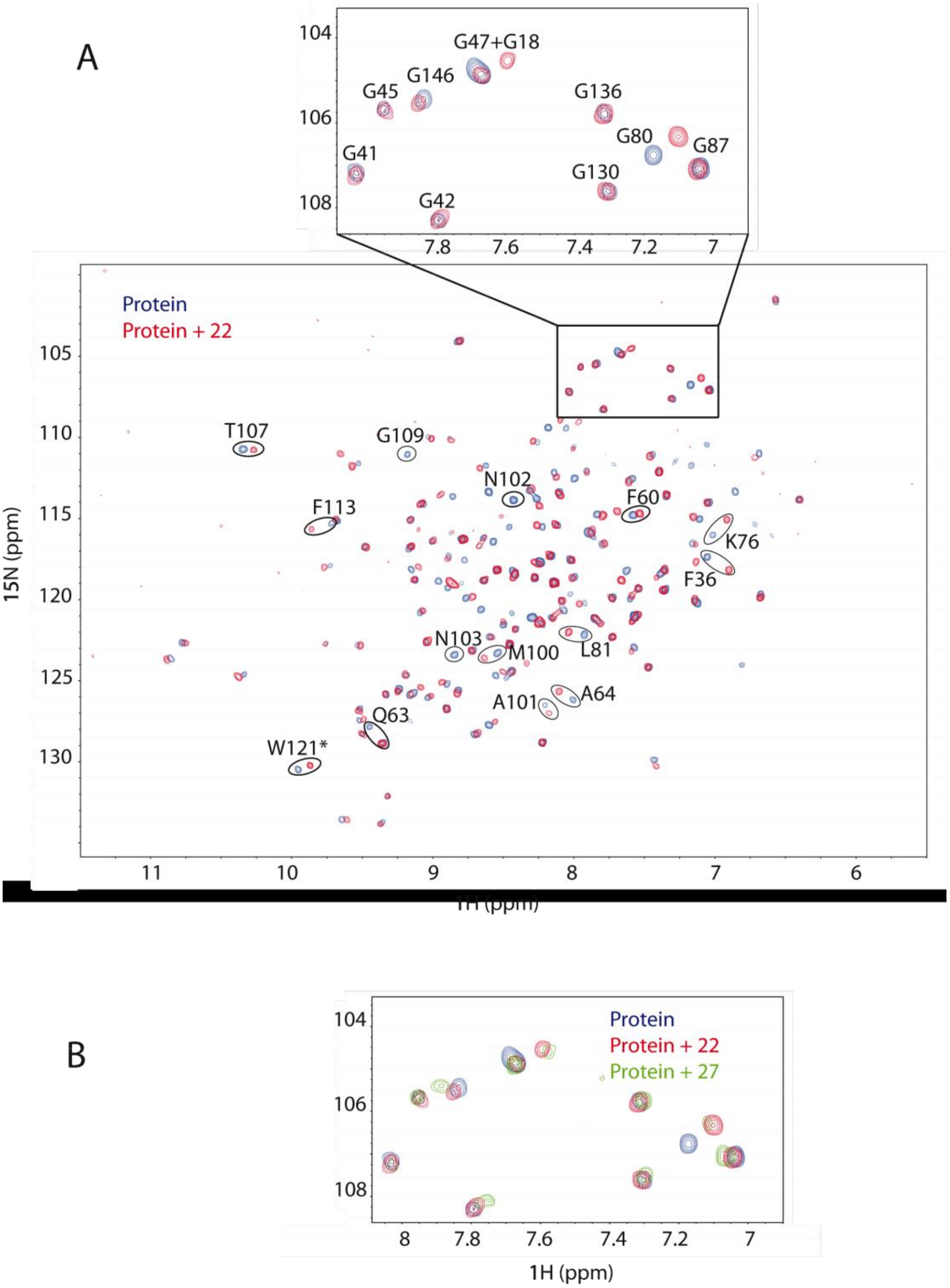

Supplementary Figure 4. Chemical shift perturbations observed in the CypD 15N-HSQC spectrum upon ligand binding. 15N-HSQC spectra were recorded with 500 µM of compound **22** or 200 µM of compound **27** and 50 µM of CypD. The zoom corresponds to the region where the glycine residues are located. W121* is labeled with a star: the peak corresponds to the amide proton of the tryptophan side chain.

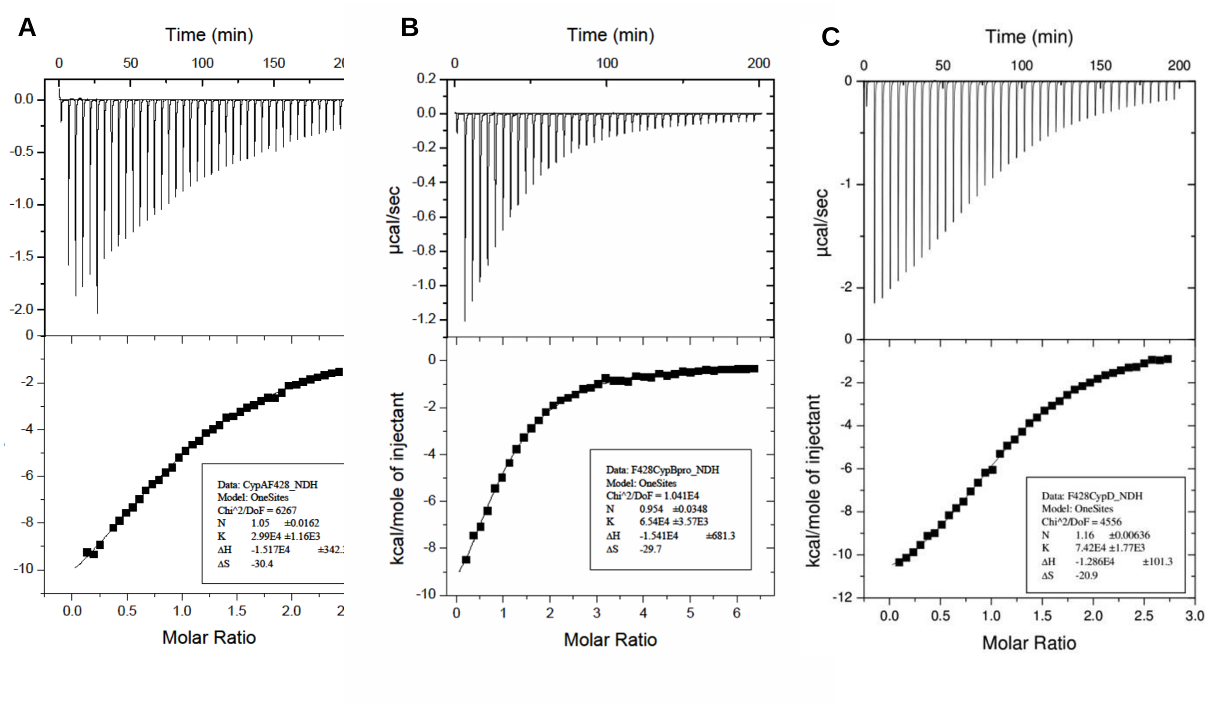

Supplementary Figure 5. Isothermal titration calorimetry (ITC) of compound 22 with cyclophilins A, B and D. (A) CypA. (B) CypB. (C) CypD.

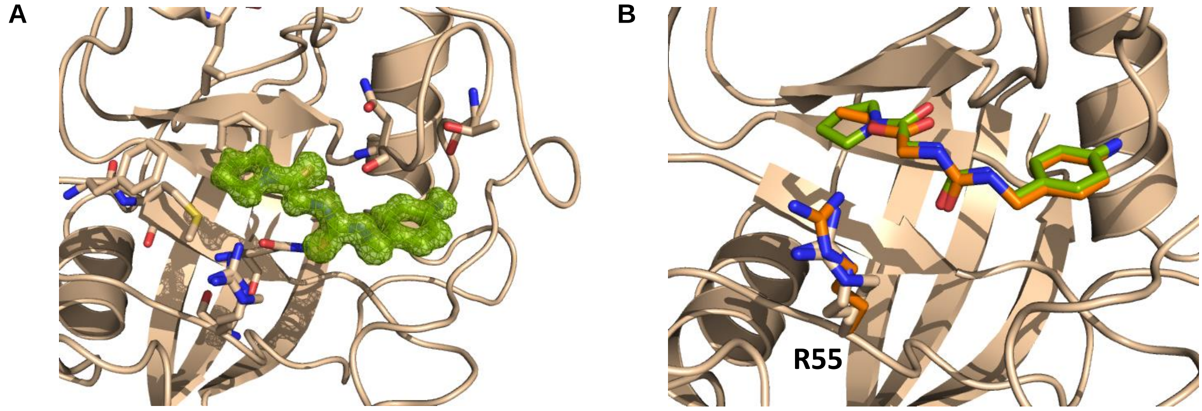

Supplementary Figure 6. Cocrystal structure of compound 23 with CypD. (A) Cartoon representation of CypD (binding residues in stick format) in complex with compound **23**, showing the occupation of both the catalytic site and gatekeeper pocket of CypD. The green mesh and surface represent the electron density map ($2F_o$–F_c omit map contoured at 1.0 σ). (B) Superimposition of CypD-compound **23** cocrystal (green sticks) and CypD-compound **22** cocrystal (orange sticks) showing identical binding modes.

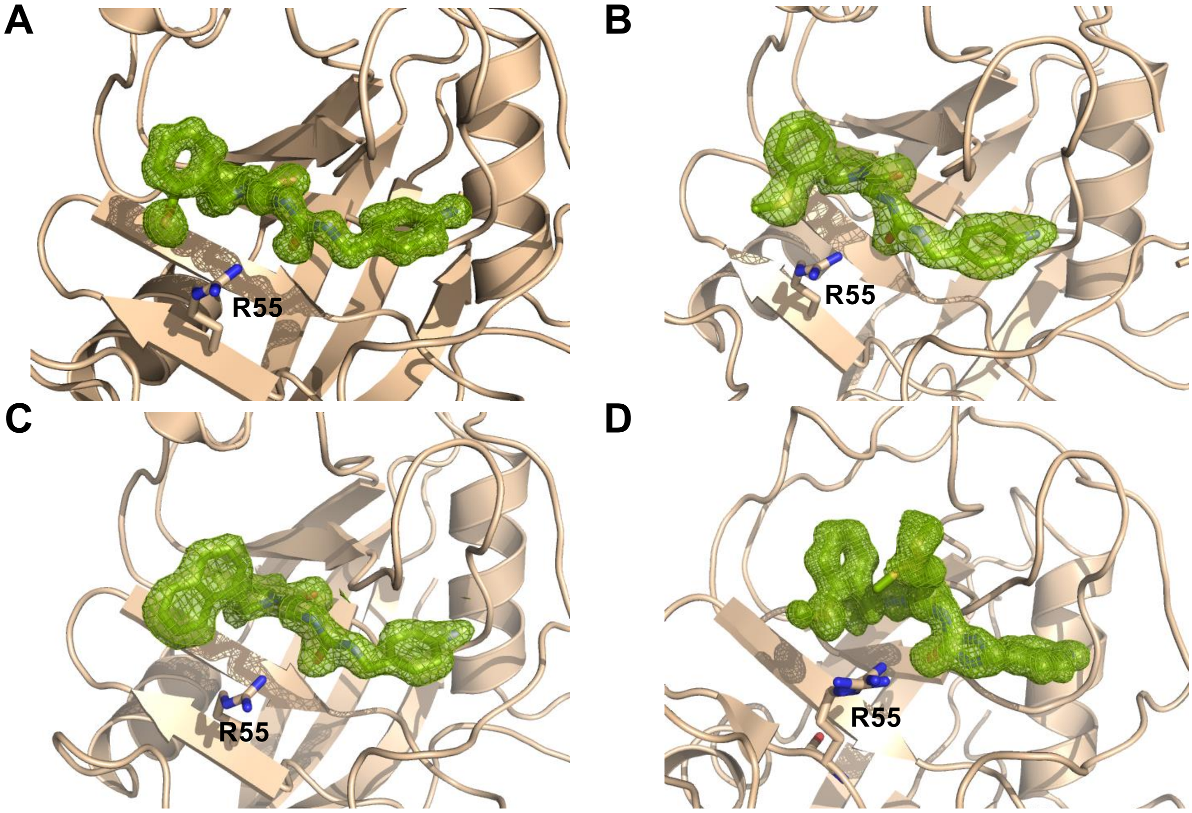

Supplementary Figure 7. Cocrystal structures of compounds 24, 26, 27, 28 and **29 with CypD.** (A) Cocrystal structure of compound **26** with CypD. (B) Cocrystal structure of compound **27** with CypD. (C) Cocrystal structure of compound **28** with CypD. (D) Cocrystal structure of compound **29** with CypD.

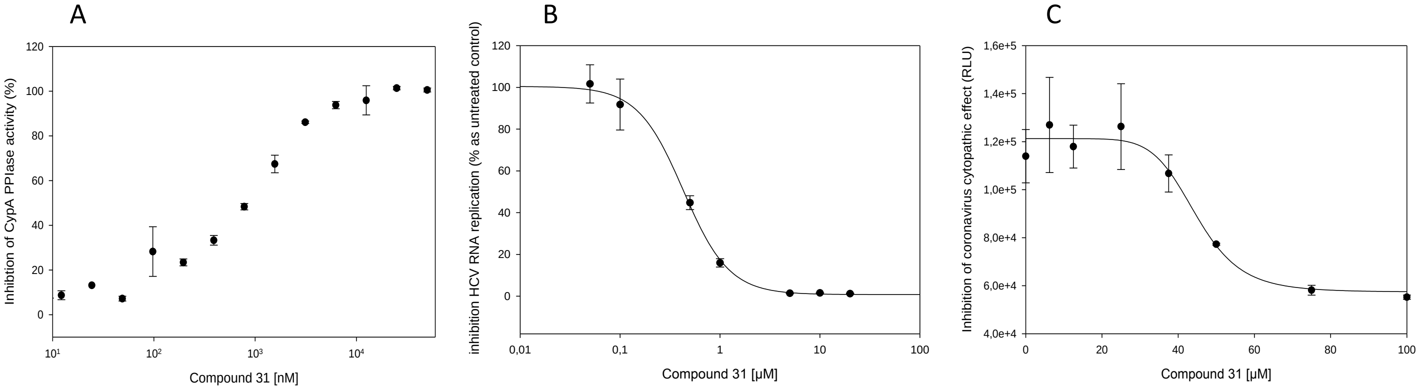

Supplementary Figure 8. Representative inhibitory inhibition curves for compound 31.

Representative curves are shown for the inhibition of: (A) CypA PPiase activity in an enzyme assay; (B) HCV replication in Huh7 cells; and (C) coronavirus HCoV-229E cytopathic effect in MRC5 cells.

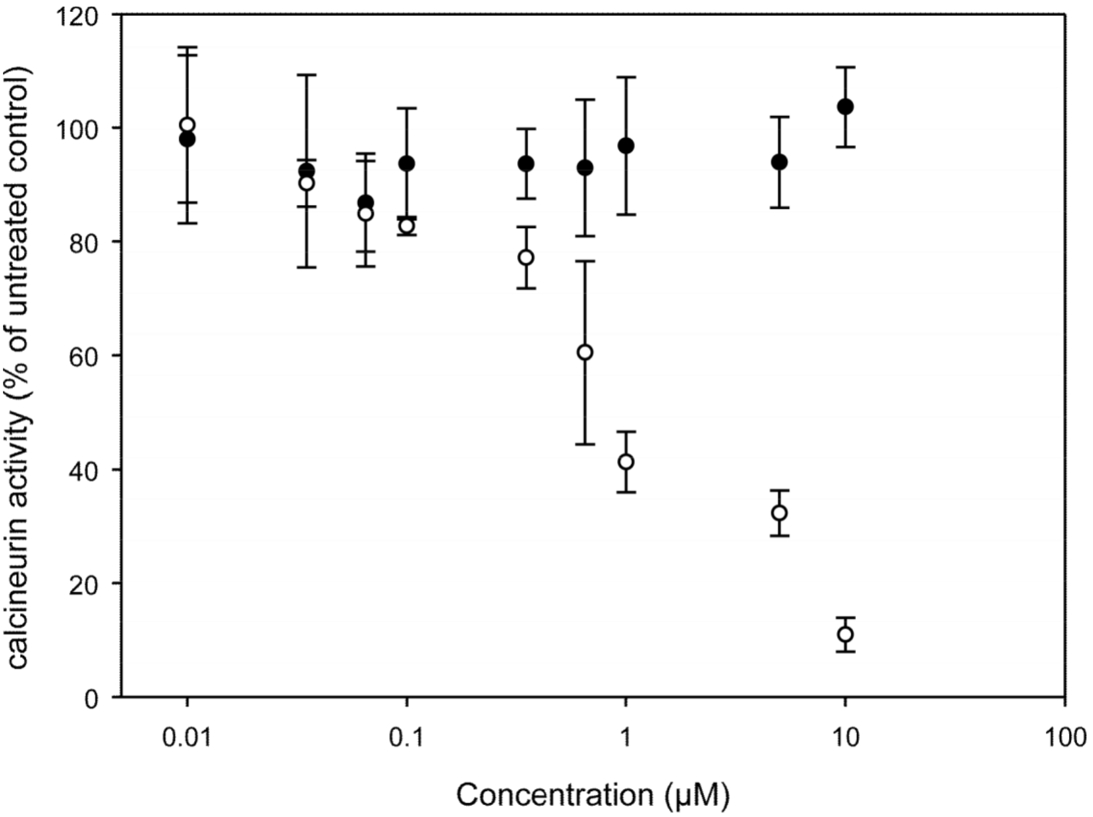

Supplementary Figure 9. Inhibition of calcineurin phosphatase activity. Calcineurin phosphatase activity was determined by measuring the dephosphorylation of p-nitrophenyl phosphate in the presence of increasing concentrations of CsA (white circles) or compound **31** (black circles) in a 1:1 molar ratio with CypA. The experiments were performed in duplicate. Mean±SD values of the percentages of inhibition of calcineurin activity relative to an inhibitor free control are shown.

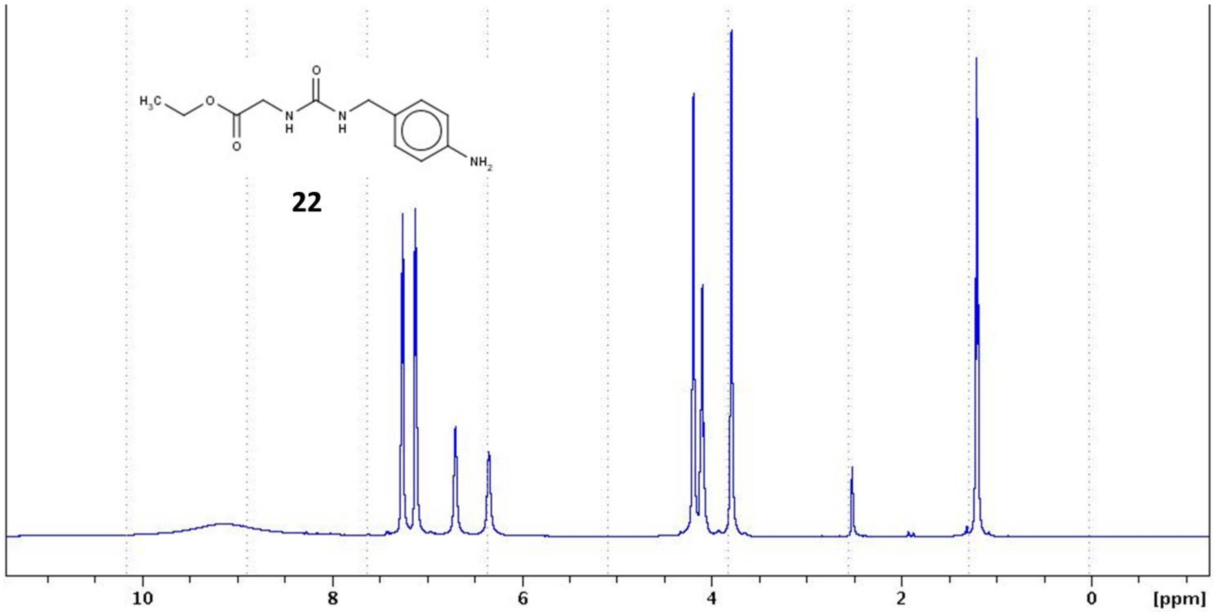

Supplementary Figure 10. ^{1}H NMR (500 MHz, DMSO-*d6*) spectrum of compound **22**.

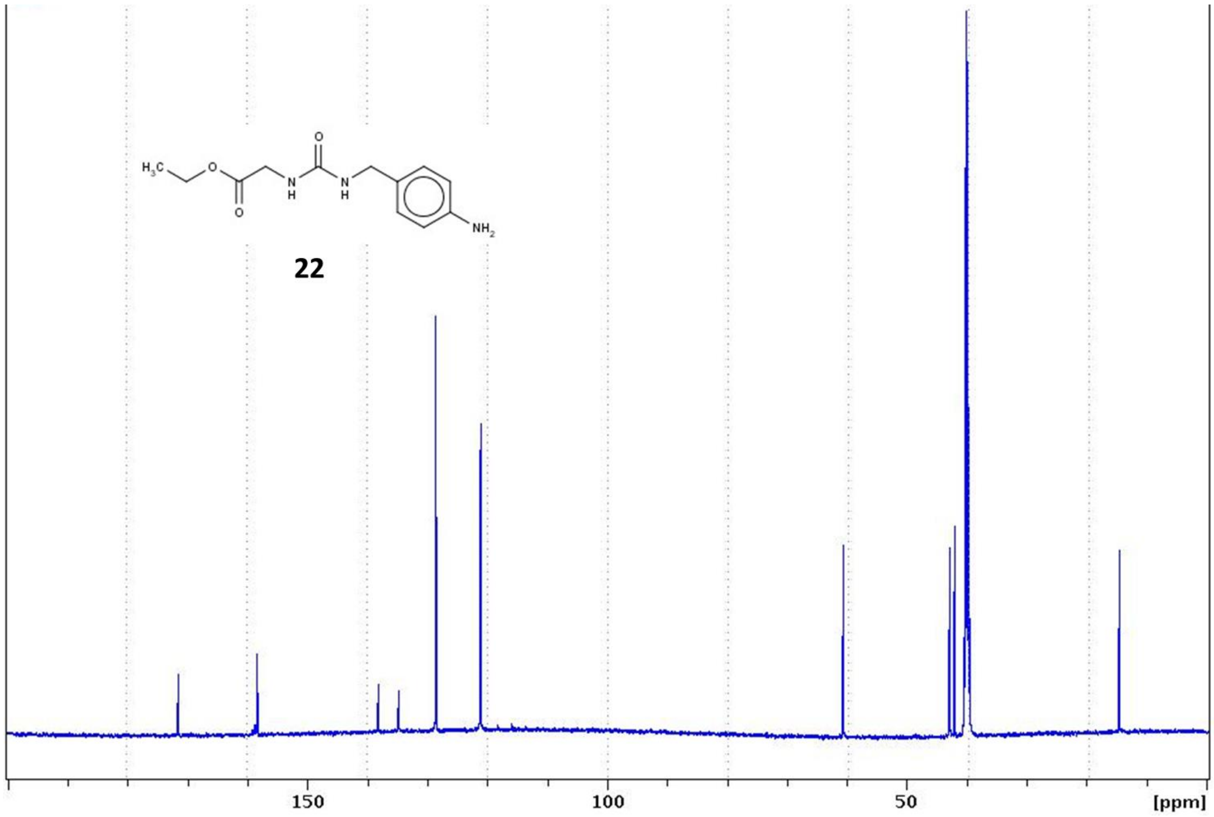

Supplementary Figure 11. ^{13}C NMR (500 MHz, DMSO-*d6*) spectrum of compound **22**.

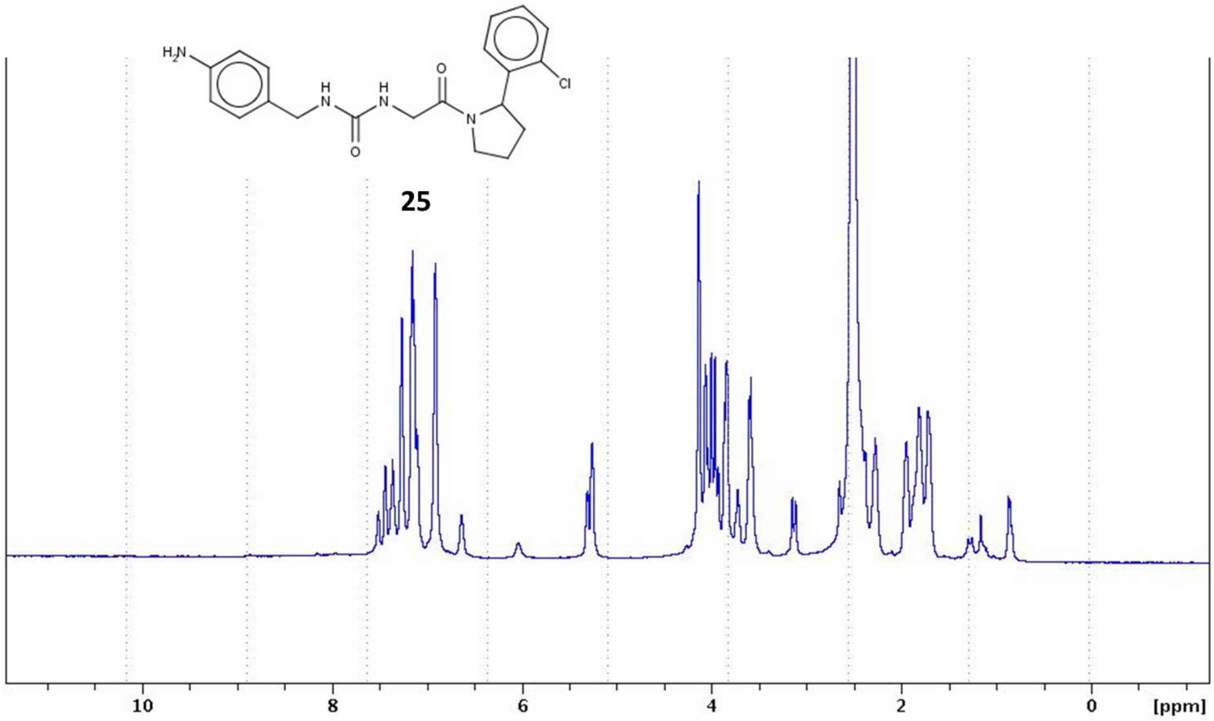

Supplementary Figure 12. ^{1}H NMR (500 MHz, DMSO-*d6*) spectrum of compound **25**.

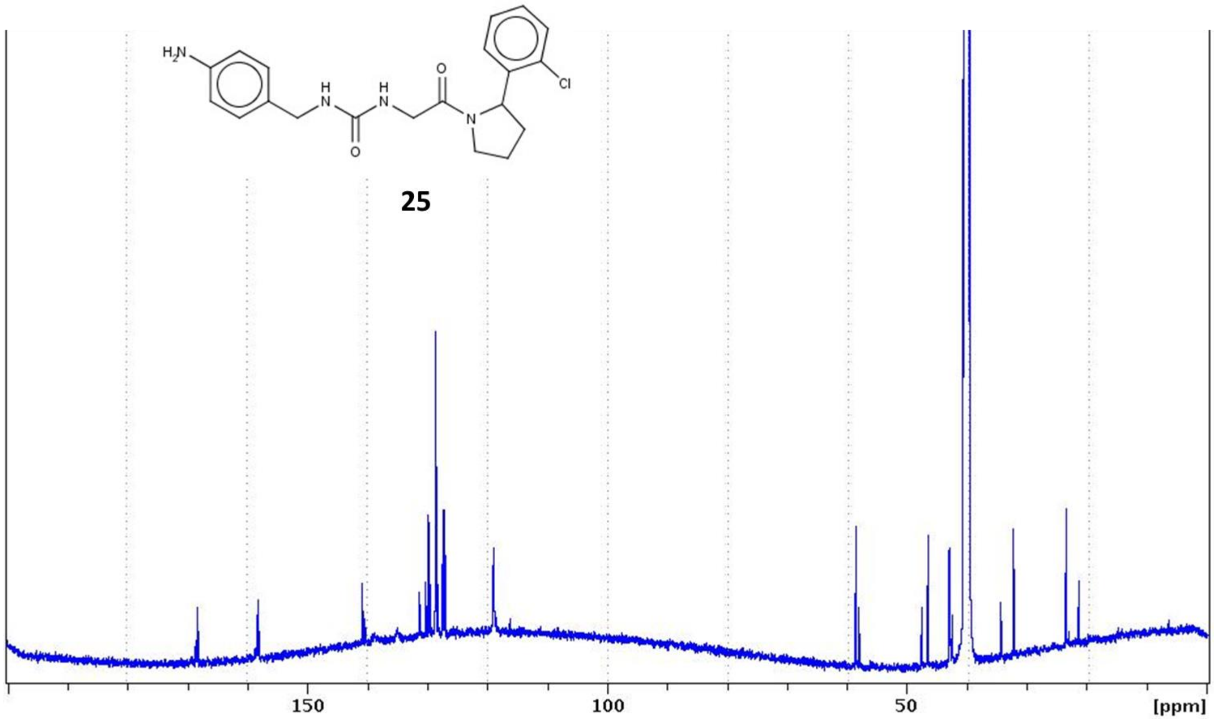

Supplementary Figure 13. ^{13}C NMR (500 MHz, DMSO-*d6*) spectrum of compound **25**.

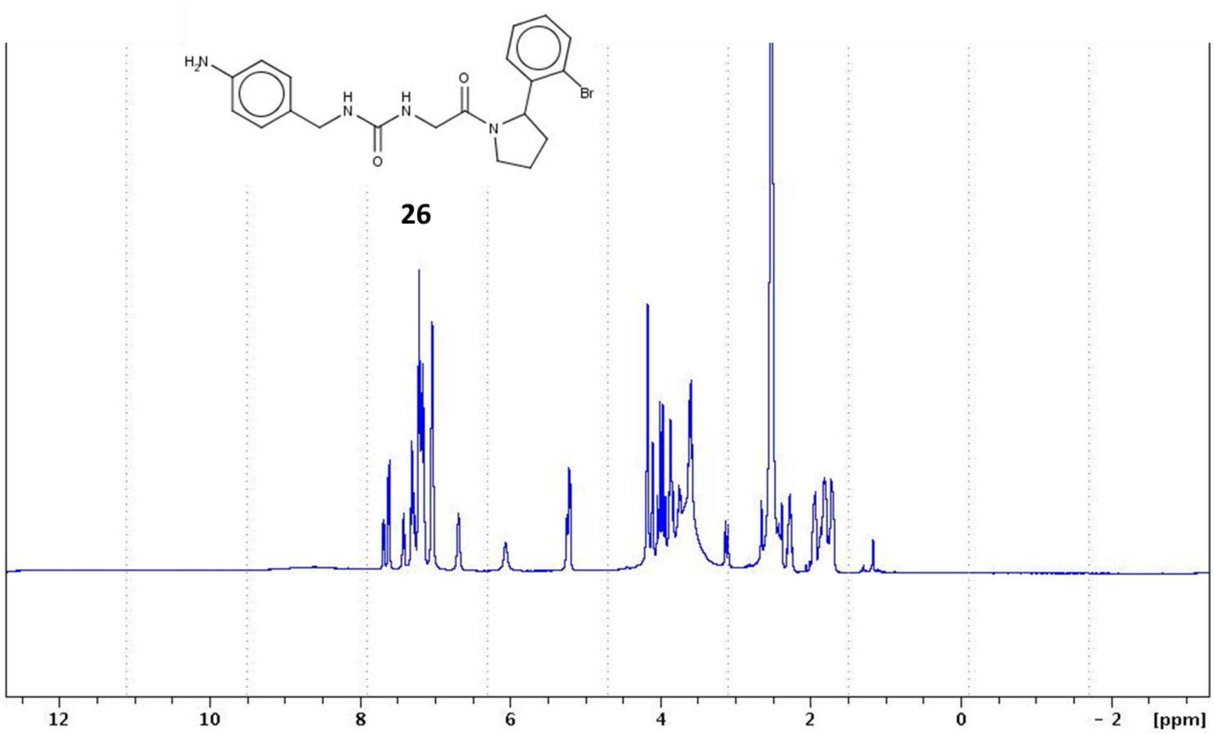

Supplementary Figure 14. ^{1}H NMR (500 MHz, DMSO-*d6*) spectrum of compound **26**.

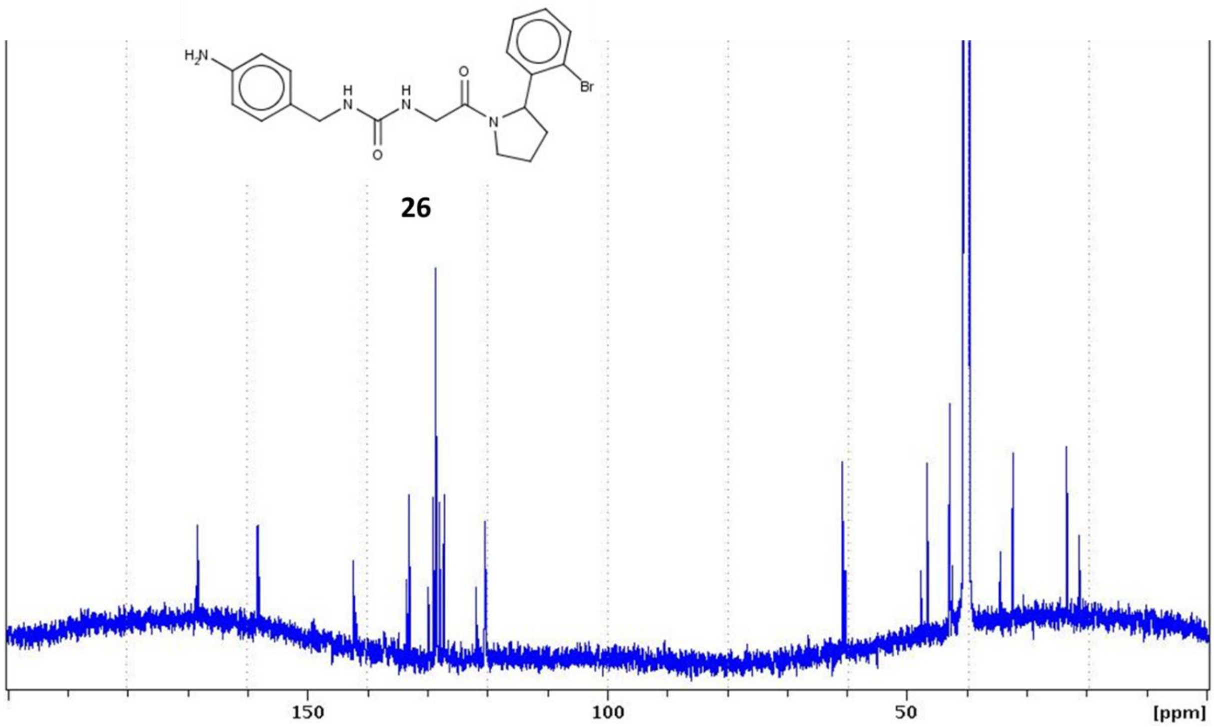

Supplementary Figure 15. ^{13}C NMR (500 MHz, DMSO-*d6*) spectrum of compound **26**.

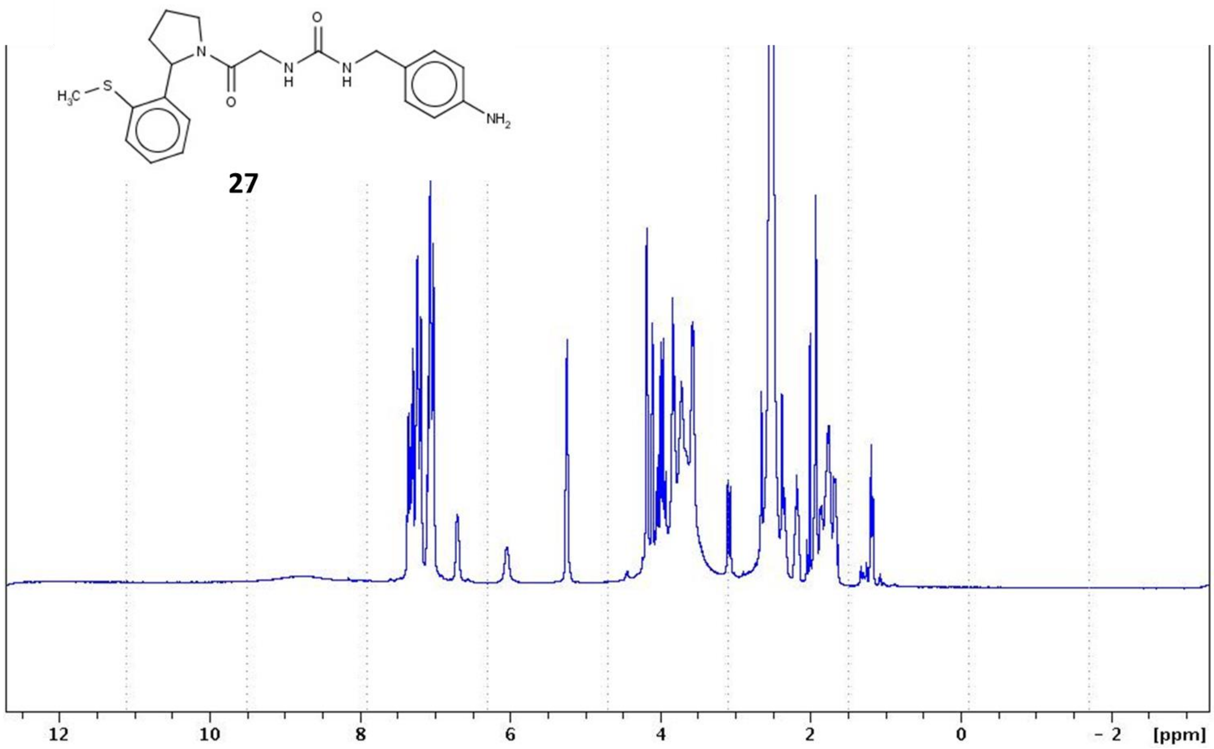

Supplementary Figure 16. ^{1}H NMR (500 MHz, DMSO-*d6*) spectrum of compound **27**.

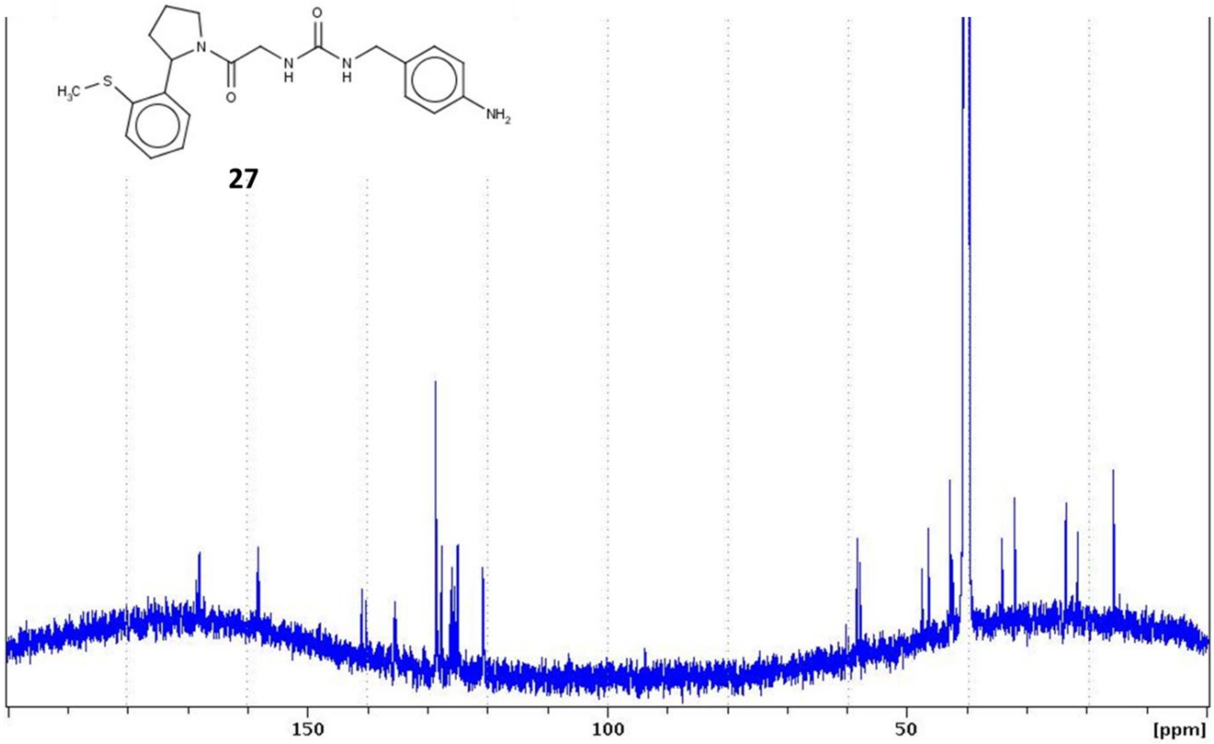

Supplementary Figure 17. ^{13}C NMR (500 MHz, DMSO-*d6*) spectrum of compound **27**.

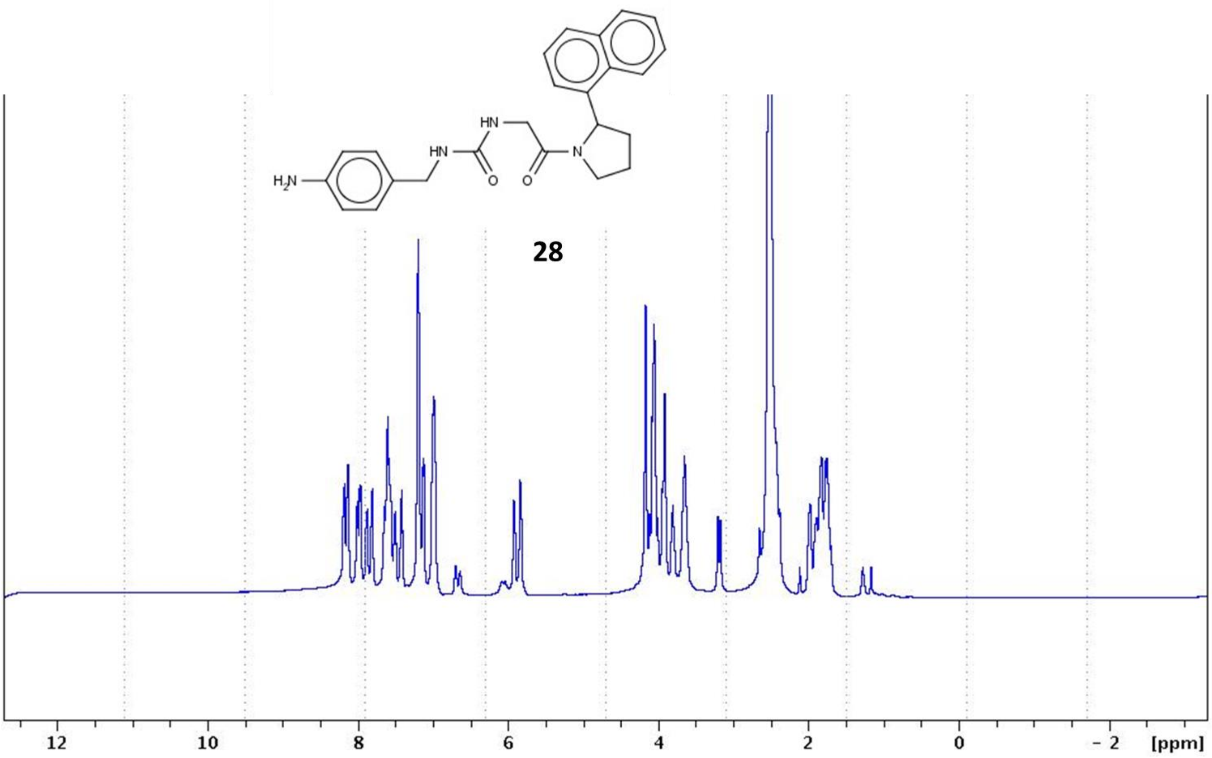

Supplementary Figure 18. ^{1}H NMR (500 MHz, DMSO-*d6*) spectrum of compound **28**.

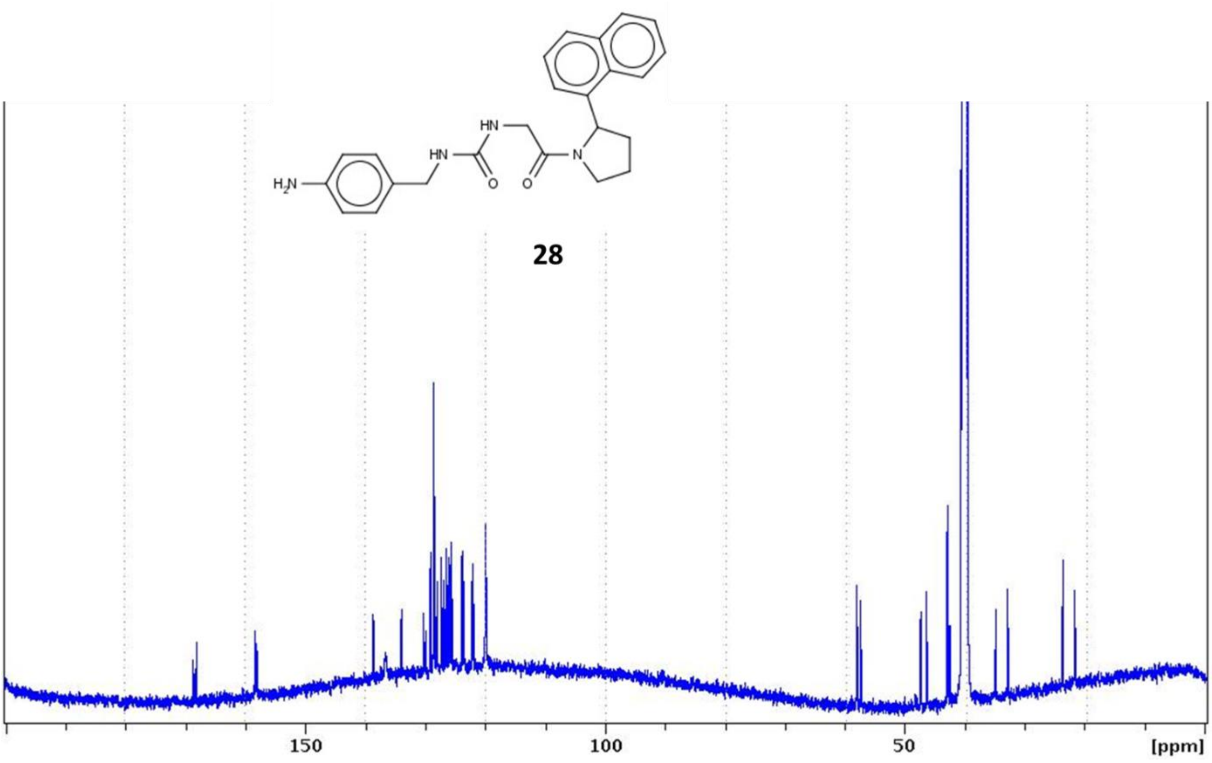

Supplementary Figure 19. ^{13}C NMR (500 MHz, DMSO-*d6*) spectrum of compound **28**.

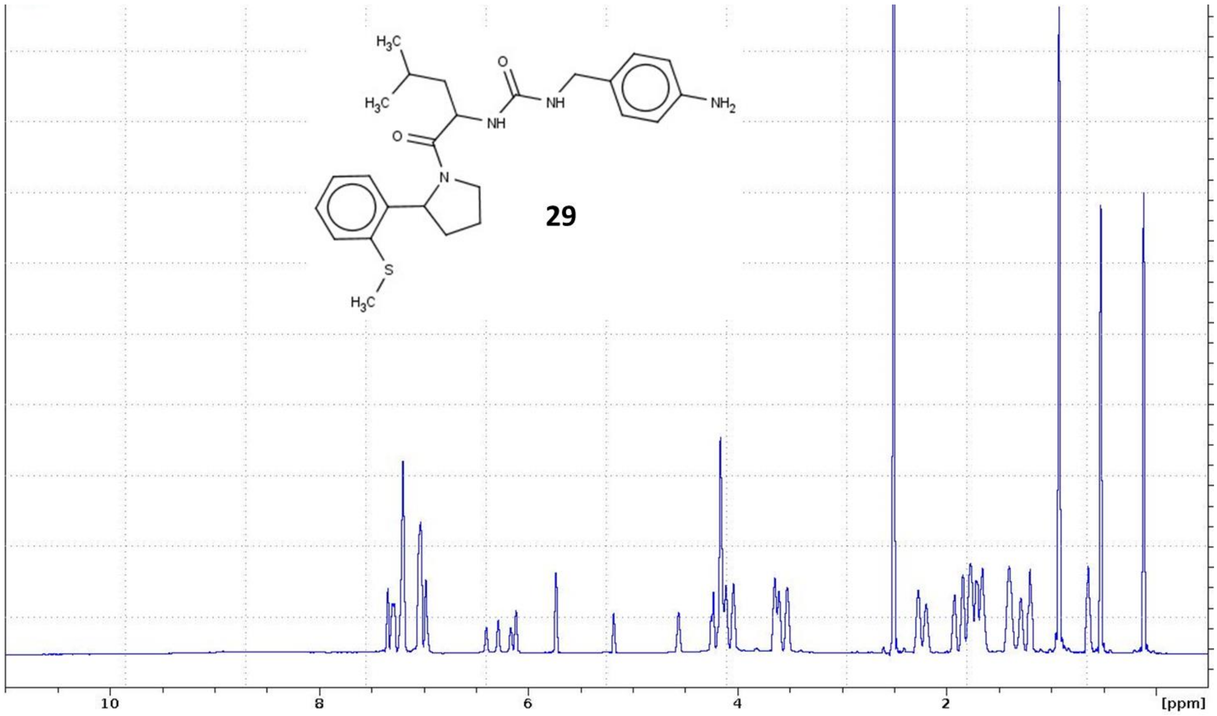

Supplementary Figure 20. ^{1}H NMR (500 MHz, DMSO-*d6*) spectrum of compound **29**.

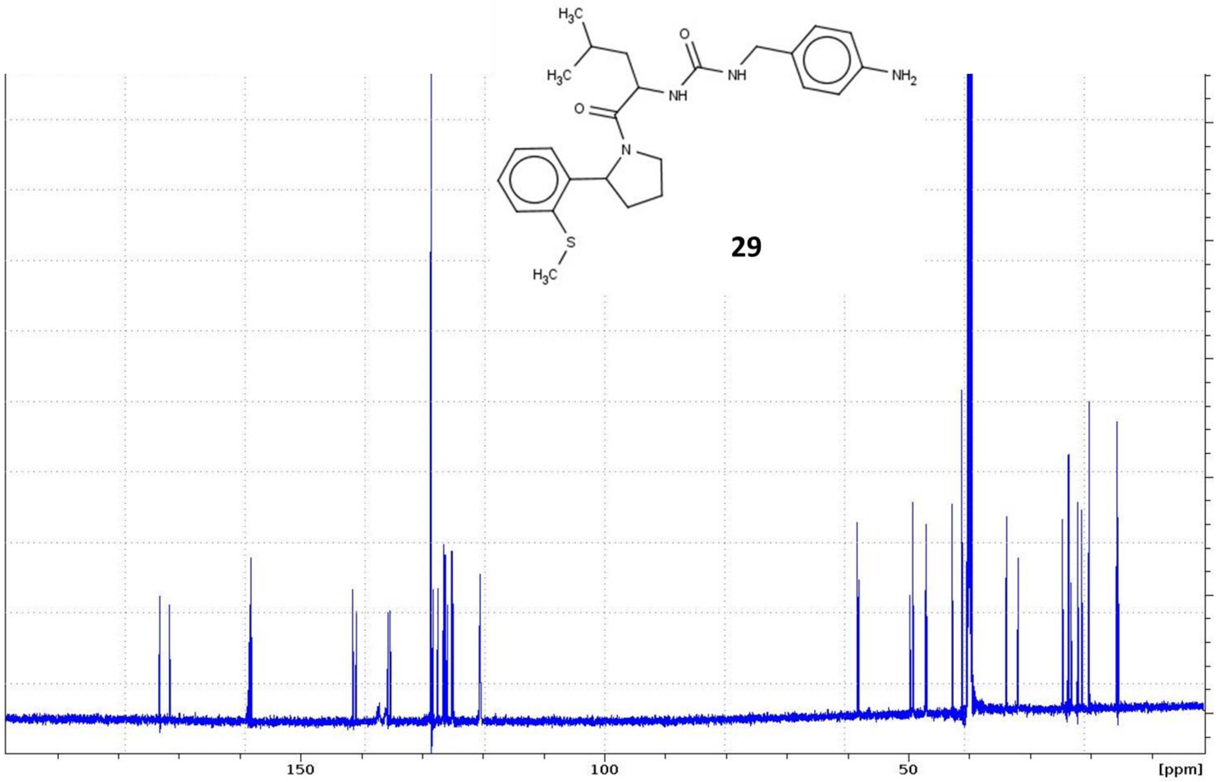

Supplementary Figure 21. ^{13}C NMR (500 MHz, DMSO-*d6*) spectrum of compound **29**.

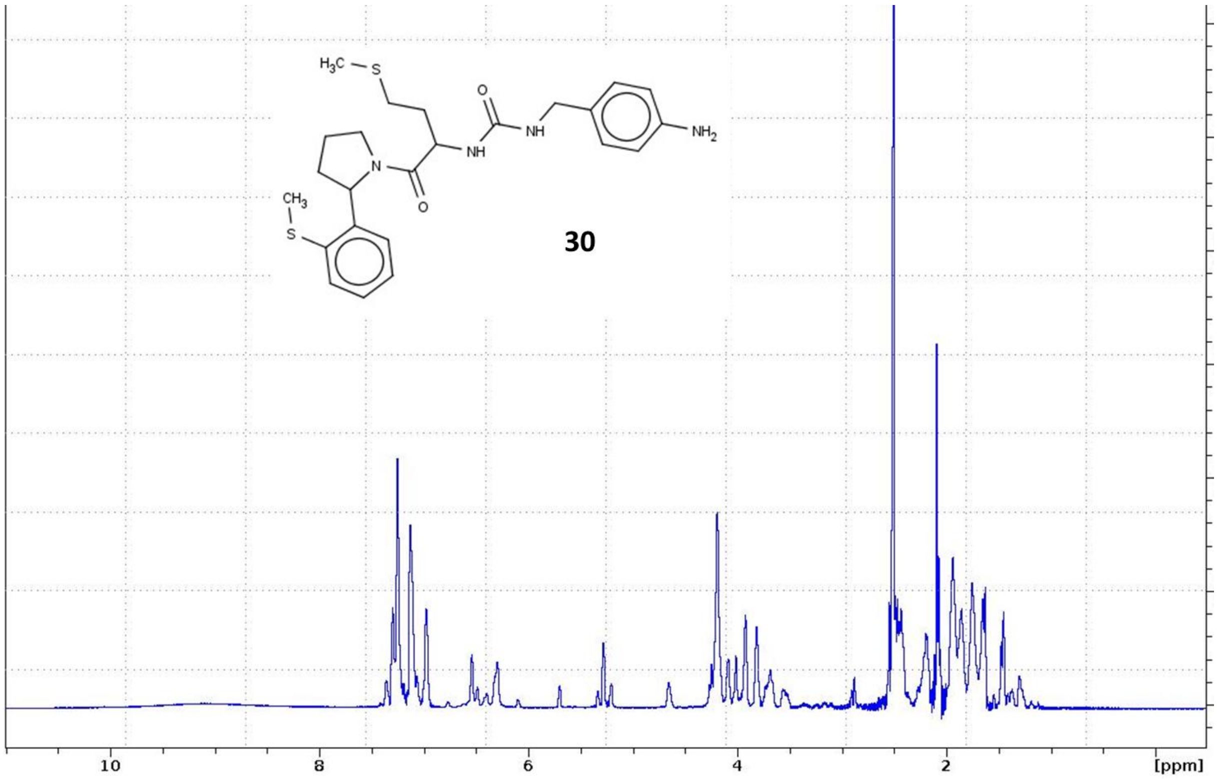

Supplementary Figure 22. ^{1}H NMR (500 MHz, DMSO-*d6*) spectrum of compound **30**.

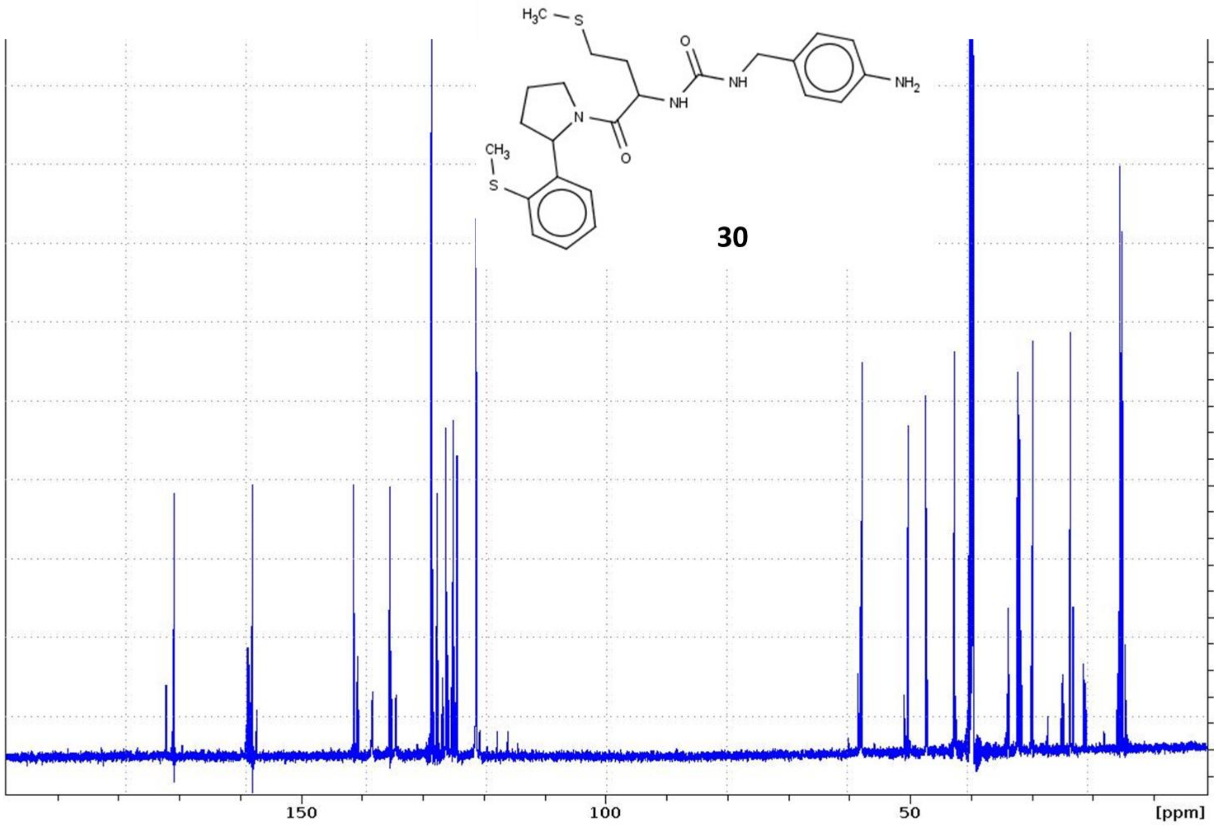

Supplementary Figure 23. ^{13}C NMR (500 MHz, DMSO-*d6*) spectrum of compound **30**.

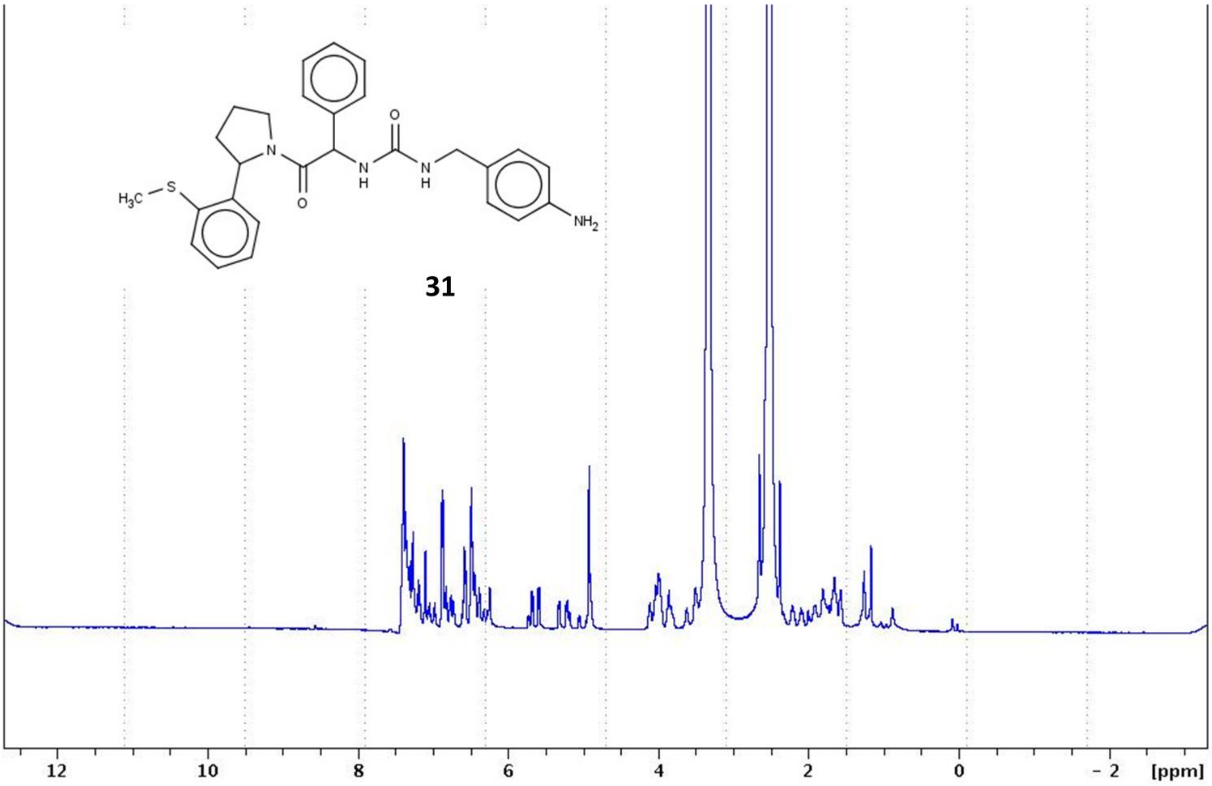

Supplementary Figure 24. ^{1}H NMR (500 MHz, DMSO-*d6*) spectrum of compound **31**.

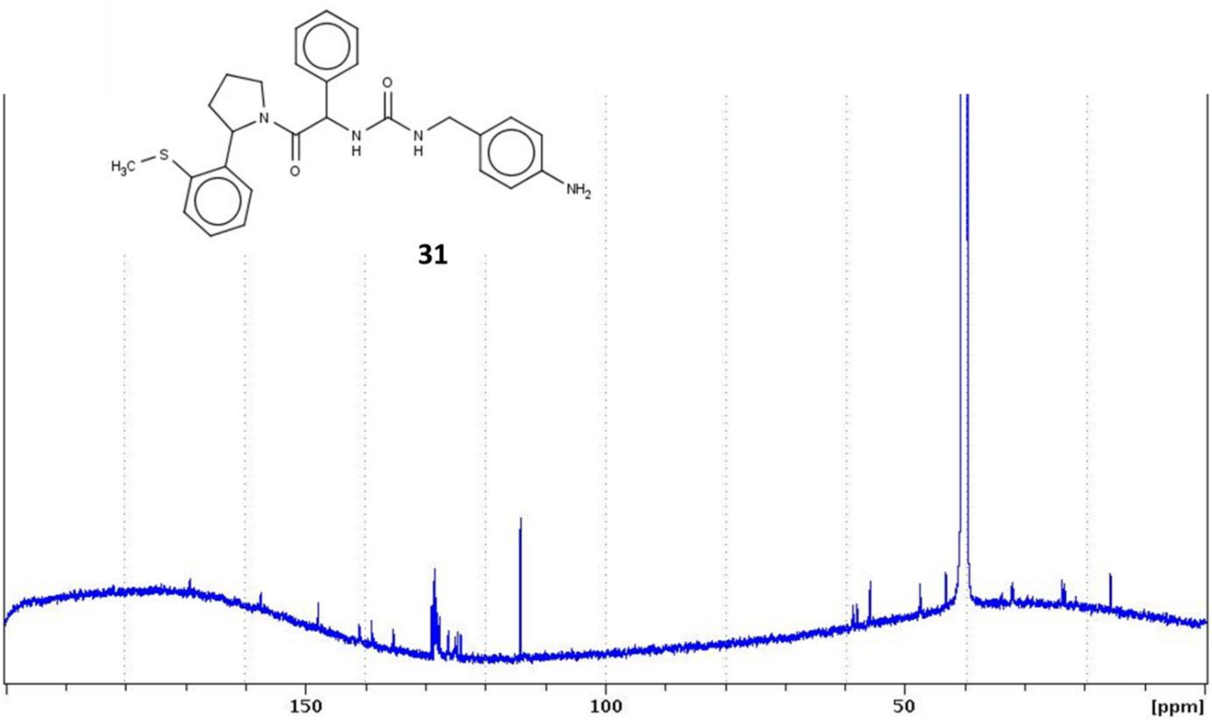

Supplementary Figure 25. ^{13}C NMR (500 MHz, DMSO-*d6*) spectrum of compound **31**.

Supplementary Tables

Supplementary Table 1. Chemical structures of aniline-free derivatives and macrocycles.

Structure	Compounds	Structure	Compounds
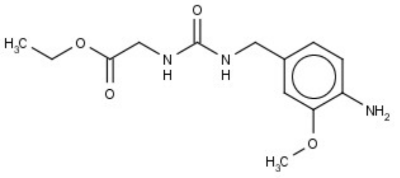	**S18**	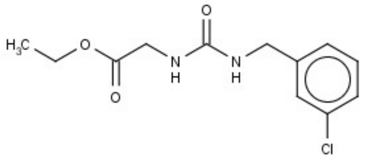	**S29**
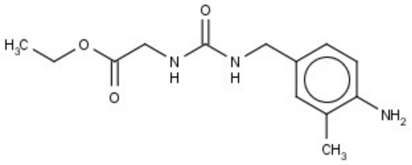	**S19**	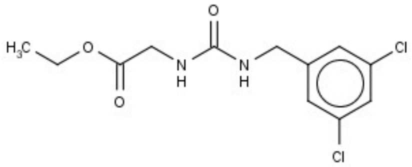	**S30**
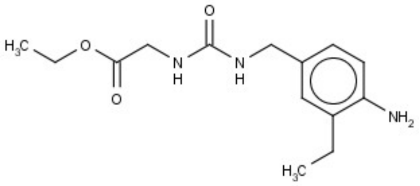	**S20**	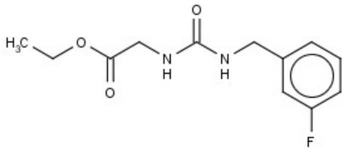	**S31**
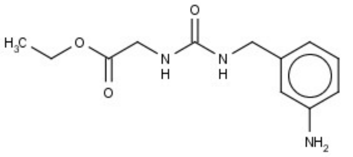	**S21**	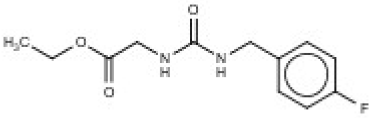	**S32**
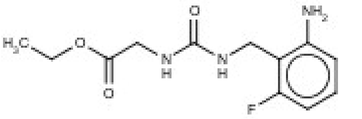	**S22**	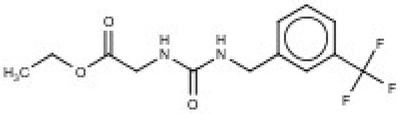	**S33**
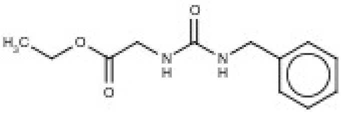	**S23**	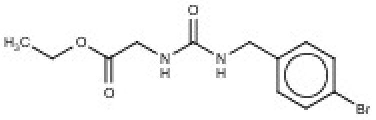	**S34**
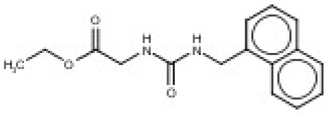	**S24**	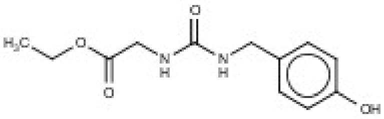	**S35**

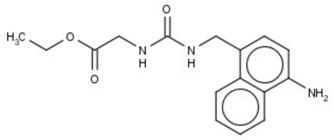	**S25**	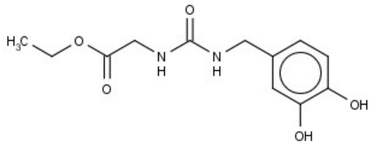	**S36**
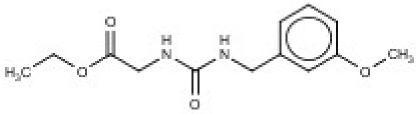	**S26**	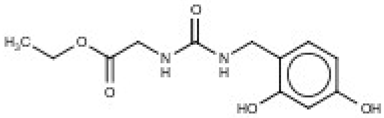	**S37**
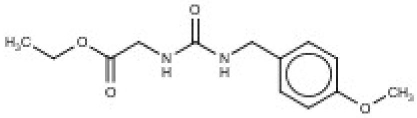	**S27**	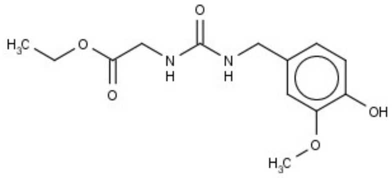	**S38**
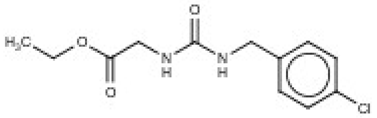	**S28**	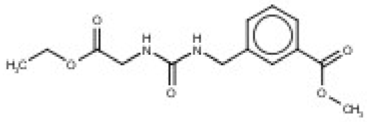	**S39**
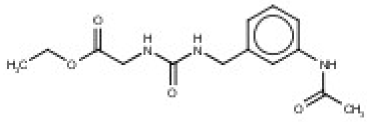	**S40**	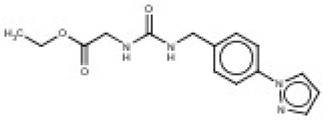	**S53**
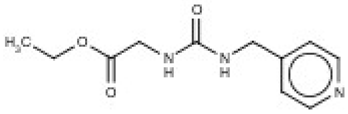	**S41**	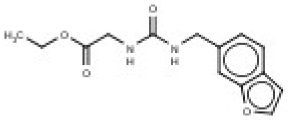	**S54**
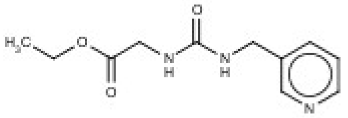	**S42**	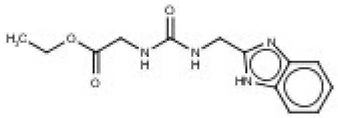	**S55**
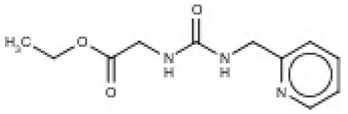	**S43**	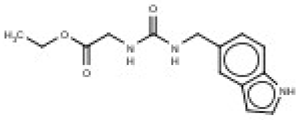	**S56**
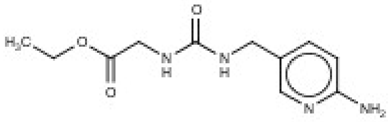	**S44**	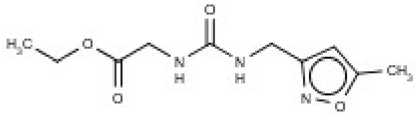	**S57**
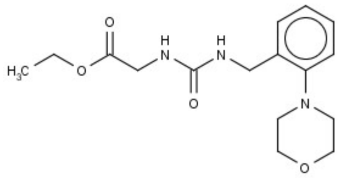	**S45**	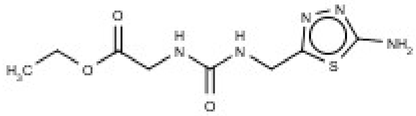	**S58**

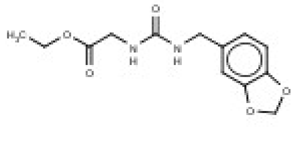	**S46**	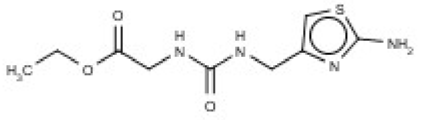	**S59**
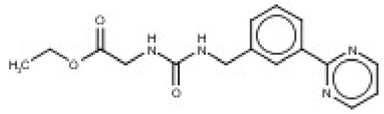	**S47**	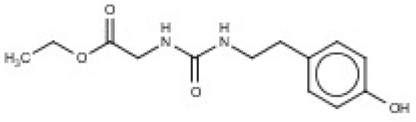	**S60**
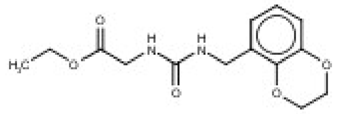	**S48**	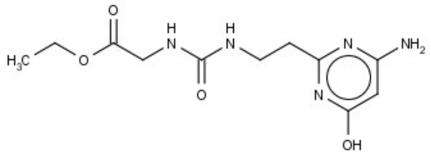	**S61**
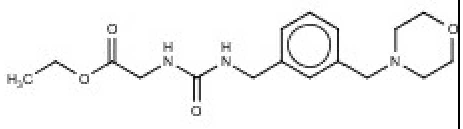	**S49**	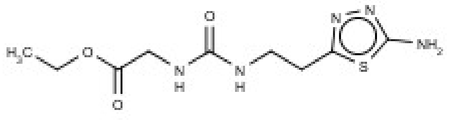	**S62**
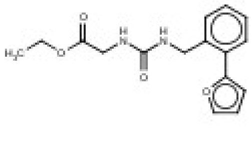	**S50**	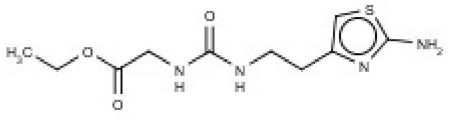	**S63**
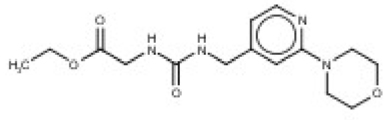	**S51**	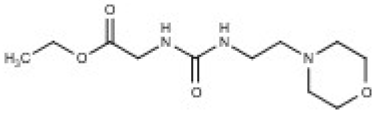	**S64**
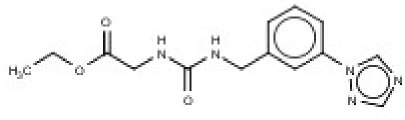	**S52**	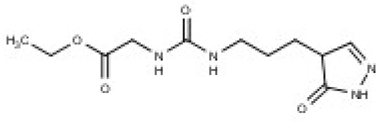	**S65**
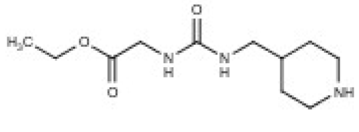	**S66**	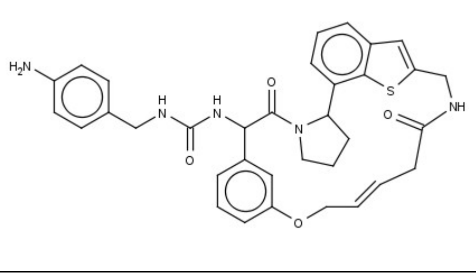	**S69**
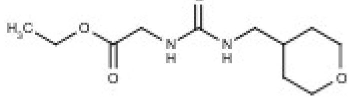	**S67**		

Supplementary Table 2. *In vitro* metabolism of compounds **29** and **30.**

	Compound **29**	Compound **30**
LogD	2.5	2.6
Solubility		
PBS (pH 7.4)	>100	>100
HCl (pH 1.0)	>100	>100
24-hour stability		
PBS (pH 7.4)	100%	100%
HCl (pH 1.0)	98%	92%
Apparent permeability (x 10^{-6} cm/s)		
Cell-free	23.6	23.8
Forward	14.0	19.5
Reverse	45.6	39.7
Ratio	3.3	2.0
Human plasma binding (%)		
Unbound	15.0	14.9
Bound	85.0	85.1
Cell culture medium (CCM) binding (%)		
Unbound	76.8	23.2
Bound	67.7	32.3
Plasma/CCM ratio	2.1	2.0
Hepatic microsomal stability, T1/2 (min)	21.6	35.5
Rate of metabolism by human cytochrome P450 (pmol/pmol P450/min)		
CYP1A2	<0.12	<0.12
CYP2B6	<0.12	<0.12
CYP2C8	0.43	0.56
CYP2C9	1.36	0.49
CYP2C19	9.96	6.21
CYD2D6	1.43	1.24
CYP3A4	<0.47	1.14
Inhibition of cytochrome P450 activity, IC50 (µM)		
CYP1A2	>25	>25
CYP2B6	>25	>25
CYP2C8	9.7	9.4
CYP2C9	>25	>25
CYP2C19	>25	>25
CYD2D6	>25	>25
CYP3A4	0.97	0.60

Supplementary Table 3. *In vitro* PPIase inhibitory activities and antiviral activities against HCV and human coronavirus 229E (HCoV-229E) of TMN 355 (purchased from Tocris Bioscience), compound A (same structure as TMN-355, synthesized) and compound B (TMN-355 derivative, synthesized). The cellular toxicities in the respective models are also shown. NT: not tested.

Compound	Structure	PPIase enzyme assay			Huh7 cells		MRC5 cells	
		CypA IC_{50} (μM)	CypB IC_{50} (μM)	CypD IC_{50} (μM)	HCV genotype 1b replicon EC_{50} (μM)	Huh7 CC_{50} (μM)	HCoV-229E EC_{50} (μM)	MRC5 cells CC_{50} (μM)
**TMN-355**	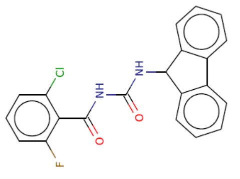	>100	>100	>100	>50	4.48±2.1	> CC50	1.04±0.46
**Compound A**	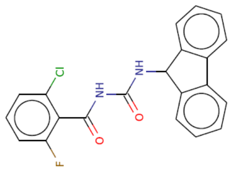	>100	>100	>100	>50	NT	NT	NT
**Compound B**	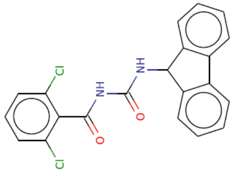	>100	>100	>100	>50	NT	NT	NT

Supplementary Table 4. Data collection and refinement statistics (molecular replacement)

(PDB accession code)	CypD-5 (3RCI)	CypD-6 (3R59)	CypD-9 (3RCF)
**Data collection**			
Space group	$P4_12_12$	$P4_12_12$	$P4_12_12$
Cell dimensions			
a, *b*, *c* (Å)	57.2,57.2,87.7	57.4,57.4,87.8	57.5, 57.5, 87.6
α, β, γ (°)	90.0, 90.0, 90.0	90.0, 90.0, 90.0	90.0, 90.0, 90.0
Resolution (Å)	29.0 (1.44) *	36.8 (1.10) *	27.3(1.15) *
R_{sym} or R_{merge}	0.067(0.491)	0.089(0.263)	0.065(0.263)
$I / \sigma I$	9.7(1.8)	15.1(3.5)	19.9(5.7)
Completeness (%)	99.9(99.6)	84.3(82.3)	98.2(90.9)
Redundancy	5.0(5.2)	8.0(7.2)	7.3(6.3)
**Refinement**			
Resolution (Å)	1.44	1.10	1.15
No. reflections	25595	50416	49247
R_{work} / R_{free}	16.8/19.7	13.4/15.3	12.5/15.0
No. atoms			
Protein	1243	1283	1331
Ligand/ion	28	12	38
Water	286	442	483
B-factors			
Protein	7.9	6.3	6.0
Ligand/ion	11.6	12.8	7.3
Water	20.9	17.1	16.5
R.m.s. deviations			
Bond lengths (Å)	0.008	0.006	0.007
Bond angles (°)	1.32	1.10	1.16

*Number of xtals for each structure should be noted in footnote. *Values in parentheses are for highest-resolution shell.

[AU: Equations defining various *R*-values are standard and hence are no longer defined in the footnotes.]
[AU: Ramachandran statistics should be in Methods section at the end of Refinement subsection.]
[AU: Wavelength of data collection, temperature and beamline should all be in Methods section.]

Supplementary Table 5. Data collection and refinement statistics (molecular replacement)

(PDB accession code)	CypD-11 (3RCG)	CypD-12 (3R4G)	CypD-13 (3R54)
**Data collection**			
Space group	$P4_12_12$	$P4_12_12$	$P4_12_12$
Cell dimensions			
a, *b*, *c* (Å)	57.4, 57.4, 87.8	57.3, 57.3, 87.2	57.4, 57.4, 87.4
α, β, γ (°)	90.0, 90.0, 90.0	90.0, 90.0, 90.0	90.0, 90.0, 90.0
Resolution (Å)	21.9 (0.97) *	29.7 (1.05) *	26.0(1.35) *
R_{sym} or R_{merge}	0.076(0.298)	0.039(0.133)	0.060(0.415)
I / σI	18.1(4.1)	14.2(4.2)	11.8(1.5)
Completeness (%)	93.3(65.9)	96.5(78.9)	95.4(85.3)
Redundancy	8.2(4.0)	5.6(2.7)	5.0(3.5)
**Refinement**			
Resolution (Å)	0.97	1.05	1.35
No. reflections	80892	65836	31104
R_{work} / R_{free}	8.98/10.84	11.8/13.9	15.0/18.4
No. atoms			
Protein	2707	1300	1274
Ligand/ion	12	26	7
Water	1677	436	448
B-factors			
Protein	4.3	5.8	7.1
Ligand/ion	5.6	9.2	13.1
Water	12.5	16.2	16.3
R.m.s. deviations			
Bond lengths (Å)	0.011	0.007	0.011
Bond angles (°)	1.57	1.22	1.38

*Number of xtals for each structure should be noted in footnote. *Values in parentheses are for highest-resolution shell.

[AU: Equations defining various *R*-values are standard and hence are no longer defined in the footnotes.]
[AU: Ramachandran statistics should be in Methods section at the end of Refinement subsection.]
[AU: Wavelength of data collection, temperature and beamline should all be in Methods section.]

Supplementary Table 6. Data collection and refinement statistics (molecular replacement)

(PDB accession code)	CypD-14 (3RD9)	CypD-15 (3R49)	CypD-16 (3R56)
**Data collection**			
Space group	$P4_12_12$	$P4_12_12$	$P4_12_12$
Cell dimensions			
a, *b*, *c* (Å)	57.2, 57.2, 87.2	56.6, 56.6, 86.8	57.0, 57.0, 87.0
α, β, γ (°)	90.0, 90.0, 90.0	90.0, 90.0, 90.0	90.0, 90.0, 90.0
Resolution (Å)	29.7 (1.40) *	29.4(1.77) *	25.5 (1.40) *
R_{sym} or R_{merge}	0.049(0.081)	0.071(0.341)	0.042(0.252)
$I / \sigma I$	29.4(8.4)	11.2(2.0)	16.5(2.7)
Completeness (%)	96.3(79.8)	99.9(99.4)	87.8(78.0)
Redundancy	7.1(2.4)	7.5(6.8)	4.8(3.8)
**Refinement**			
Resolution (Å)	1.40	1.77	1.40
No. reflections	28209	14229	23793
R_{work} / R_{free}	12.2/15.0	18.8/23.5	15.8/17.7
No. atoms			
Protein	1280	1270	1254
Ligand/ion	17	11	10
Water	468	295	321
B-factors			
Protein	8.5	14.7	7.8
Ligand/ion	9.3	31.3	13.8
Water	11.3	30.3	19.1
R.m.s. deviations			
Bond lengths (Å)	0.006	0.006	0.005
Bond angles (°)	1.19	1.11	0.98

*Number of xtals for each structure should be noted in footnote. *Values in parentheses are for highest-resolution shell.

[AU: Equations defining various *R*-values are standard and hence are no longer defined in the footnotes.]
[AU: Ramachandran statistics should be in Methods section at the end of Refinement subsection.]
[AU: Wavelength of data collection, temperature and beamline should all be in Methods section.]

Supplementary Table 7. Data collection and refinement statistics (molecular replacement)

(PDB accession code)	CypD-17 (3RCL)	CypD-18 (3RDB)	CypD-19 (3RCK)
**Data collection**			
Space group	$P4_12_12$	$P4_12_12$	$P4_12_12$
Cell dimensions			
a, *b*, *c* (Å)	57.1, 57.1, 87.5	57.0, 57.0, 87.0	57.3, 57.3, 87.4
α, β, γ (°)	90.0, 90.0, 90.0	90.0, 90.0, 90.0	90.0, 90.0, 90.0
Resolution (Å)	22.0(1.70) *	27.2 (1.55)*	36.8 (1.26)*
R_{sym} or R_{merge}	0.020(0.037)	0.059 (0.291)	0.043 (0.487)
$I / \sigma I$	32.6(15.2)	14.0 (3.8)	12.3 (1.3)
Completeness (%)	98.1(87.4)	100 (100)	96.3 (77.8)
Redundancy	5.9(3.7)	6.8 (6.2)	5.2 (2.4)
**Refinement**			
Resolution (Å)	1.70	1.55	1.26
No. reflections	16182	21711	38560
R_{work} / R_{free}	13.2/16.9	13.8/18.2	16.4/18.1
No. atoms			
Protein	1270	1280	1278
Ligand/ion	12	14	24
Water	360	394	251
B-factors			
Protein	6.3	6.4	9.2
Ligand/ion	14.0	11.6	16.5
Water	19.4	16.8	21.3
R.m.s. deviations			
Bond lengths (Å)	0.006	0.008	0.008
Bond angles (°)	1.04	1.23	1.23

*Number of xtals for each structure should be noted in footnote. *Values in parentheses are for highest-resolution shell.

[AU: Equations defining various *R*-values are standard and hence are no longer defined in the footnotes.]
[AU: Ramachandran statistics should be in Methods section at the end of Refinement subsection.]
[AU: Wavelength of data collection, temperature and beamline should all be in Methods section.]

Supplementary Table 8. Data collection and refinement statistics (molecular replacement)

(PDB accession code)	CypD-20 (3RDA)	CypD-21 (3R57)	CypD-22 (3RDC)
**Data collection**			
Space group	$P4_12_12$	$P4_12_12$	$P4_12_12$
Cell dimensions			
a, *b*, *c* (Å)	57.2, 57.2, 87.0	57.1, 57.1, 87.5	56.4, 56.4, 87.0
α, β, γ (°)	90.0, 90.0, 90.0	90.0, 90.0, 90.0	90.0, 90.0, 90.0
Resolution (Å)	29.0(1.07) *	22.0(1.70) *	28.2(1.93) *
R_{sym} or R_{merge}	0.034(0.159)	0.020(0.037)	0.031(0.188)
I / σI	12.7(3.3)	32.6(15.2)	9.1(3.5)
Completeness (%)	95.4(76.8)	98.1(87.4)	97.5(84.0)
Redundancy	4.6(3.1)	5.9(3.7)	4.1(4.8)
**Refinement**			
Resolution (Å)	1.07	1.70	1.93
No. reflections	61282	16182	10358
R_{work} / R_{free}	11.5/13.8	13.2/16.9	20.8/27.0
No. atoms			
Protein	1279	1301	1232
Ligand/ion	49	44	18
Water	415	366	223
B-factors			
Protein	5.6	6.2	15.8
Ligand/ion	8.8	12.7	17.5
Water	15.6	19.4	24.1
R.m.s. deviations			
Bond lengths (Å)	0.009	0.006	0.007
Bond angles (°)	1.55	1.04	1.47

*Number of xtals for each structure should be noted in footnote. *Values in parentheses are for highest-resolution shell.

[AU: Equations defining various *R*-values are standard and hence are no longer defined in the footnotes.]
[AU: Ramachandran statistics should be in Methods section at the end of Refinement subsection.]
[AU: Wavelength of data collection, temperature and beamline should all be in Methods section.]

Supplementary Table 9. Data collection and refinement statistics (molecular replacement)

(PDB accession code)	CypA-22 (3RDD)	CypD-23 (4J58)	CypD-24 (4J5E)
**Data collection**			
Space group	$P2_12_12_1$	$P4_12_12$	$P4_12_12$
Cell dimensions			
a, *b*, *c* (Å)	40.7, 52.3, 86.9	57.2, 57.2, 87.6	57.0, 57.0, 87.8
α, β, γ (°)	90.0, 90.0, 90.0	90.0, 90.0, 90.0	90.0, 90.0, 90.0
Resolution (Å)	35.0(2.14) *	36.76 (1.28) *	15.94(0.99) *
R_{sym} or R_{merge}	0.051(0.494)	0.037(0.323)	0.071(0.398)
$I / \sigma I$	11.3(1.6)	12.9(1.7)	10.5(2.2)
Completeness (%)	98.7(97.2)	96.1(77.2)	98.0(87.5)
Redundancy	2.5(2.6)	3.5(2.1)	5.9(2.7)
**Refinement**			
Resolution (Å)	2.14	1.28	0.99
No. reflections	10210	36729	794719
R_{work} / R_{free}	21.8/27.1	11.9/15.8	11.9/13.6
No. atoms			
Protein	1249	1280	2751
Ligand/ion	18	20	28
Water	95	377	522
B-factors			
Protein	29.7	6.7	8.2
Ligand/ion	32.8	9.4	7.0
Water	37.8	20.3	21.1
R.m.s. deviations			
Bond lengths (Å)	0.010	0.007	0.008
Bond angles (°)	1.23	1.44	1.35

*Number of xtals for each structure should be noted in footnote. *Values in parentheses are for highest-resolution shell.

[AU: Equations defining various *R*-values are standard and hence are no longer defined in the footnotes.]
[AU: Ramachandran statistics should be in Methods section at the end of Refinement subsection.]
[AU: Wavelength of data collection, temperature and beamline should all be in Methods section.]

Supplementary Table 10. Data collection and refinement statistics (molecular replacement)

(PDB accession code)	CypD-26 (4J5D)	CypD-27 (4J5B)	CypD-28 (4J59)
**Data collection**			
Space group	$P4_12_12$	$P4_12_12$	$P4_12_12$
Cell dimensions			
a, *b*, *c* (Å)	57.2, 57.2, 87.6	57.8, 57.8, 88.8	57.1, 57.1, 87.6
α, β, γ (°)	90.0, 90.0, 90.0	90.0, 90.0, 90.0	90.0, 90.0, 90.0
Resolution (Å)	36.76 (1.28) *	30.07(2.01) *	57.07 (1.92) *
R_{sym} or R_{merge}	0.037(0.323)	0.062(0.285)	0.109(0.489)
I / σI	12.9(1.7)	12.6(3.3)	13.5(2.8)
Completeness (%)	96.1(77.2)	97.1(90.7)	93.5(86.4)
Redundancy	3.5(2.1)	4.2(4.0)	6.8(8.5)
**Refinement**			
Resolution (Å)	1.28	2.01	1.92
No. reflections	36729	10410	10801
R_{work} / R_{free}	11.9/15.8	15.6/18.4	15.1/19.9
No. atoms			
Protein	1280	1243	1248
Ligand/ion	20	28	30
Water	377	84	250
B-factors			
Protein	6.7	18.3	7.9
Ligand/ion	9.4	34.8	11.7
Water	20.3	27.8	19.0
R.m.s. deviations			
Bond lengths (Å)	0.007	0.007	0.008
Bond angles (°)	1.44	1.21	1.22

*Number of xtals for each structure should be noted in footnote. *Values in parentheses are for highest-resolution shell.

[AU: Equations defining various *R*-values are standard and hence are no longer defined in the footnotes.]
[AU: Ramachandran statistics should be in Methods section at the end of Refinement subsection.]
[AU: Wavelength of data collection, temperature and beamline should all be in Methods section.]

Supplementary Table 11. Data collection and refinement statistics (molecular replacement)

	CypD-29
(PDB accession code)	
**Data collection**	
Space group	$P4_12_12$
Cell dimensions	
a, *b*, *c* (Å)	57.3, 57.3, 87.5
α, β, γ (°)	90.0, 90.0, 90.0
Resolution (Å)	47.95(1.03) *
R_{sym} or R_{merge}	0.087(0.216)
I / σI	18.8(3.9)
Completeness (%)	96.9(79.4)
Redundancy	10.5(3.3)
**Refinement**	
Resolution (Å)	1.03
No. reflections	32279
R_{work} / R_{free}	12.3/16.2
No. atoms	
Protein	1302
Ligand/ion	32
Water	463
B-factors	
Protein	12.2
Ligand/ion	14.9
Water	23.8
R.m.s. deviations	
Bond lengths (Å)	0.011
Bond angles (°)	1.66

*Number of xtals for each structure should be noted in footnote. *Values in parentheses are for highest-resolution shell.

[AU: Equations defining various *R*-values are standard and hence are no longer defined in the footnotes.]
[AU: Ramachandran statistics should be in Methods section at the end of Refinement subsection.]
[AU: Wavelength of data collection, temperature and beamline should all be in Methods section.]

Supplementary Methods

Scheme 1. Synthesis of compounds **22** to **28**.

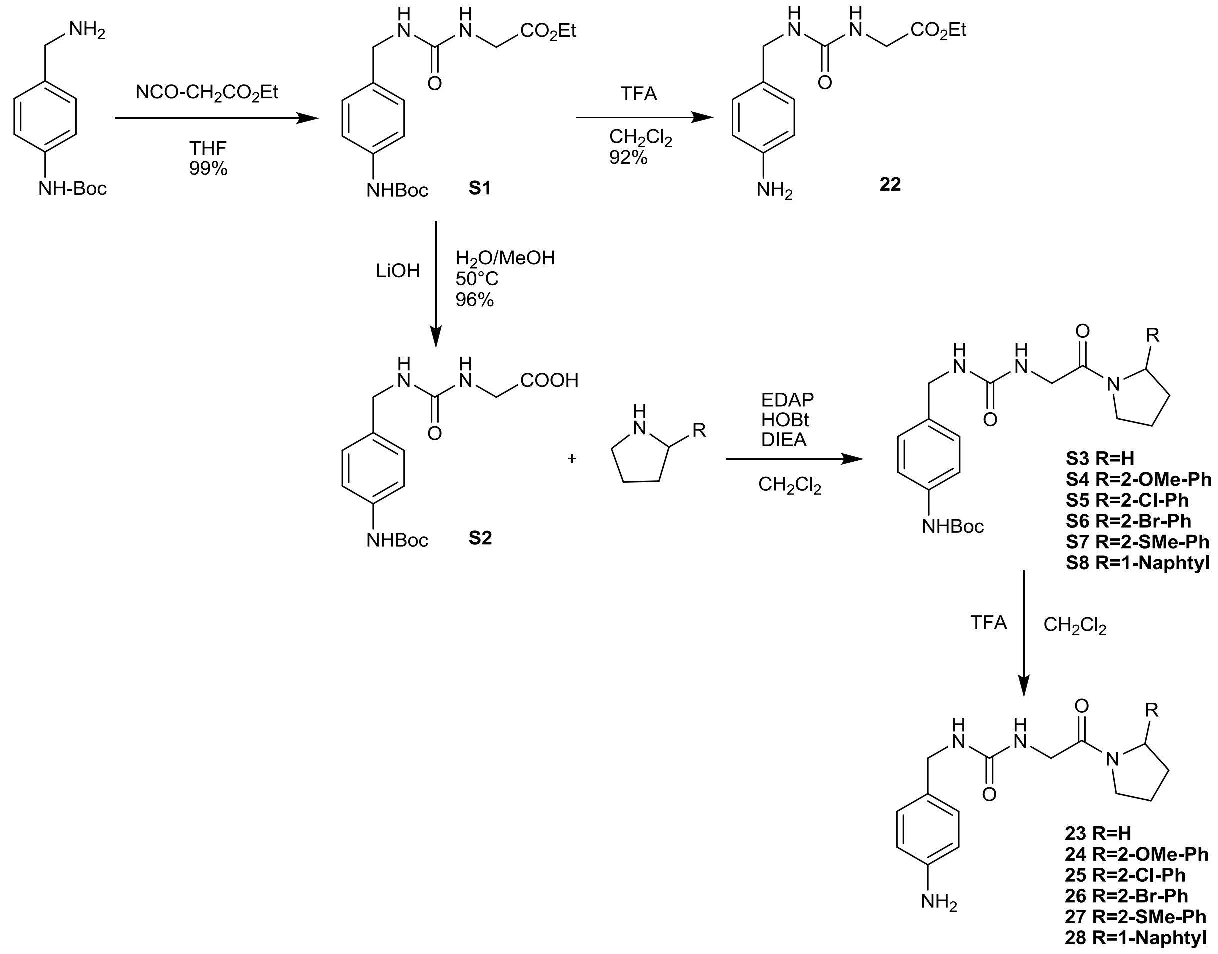

Synthesis of **ethyl 2-(3-(4-(tert-butoxycarbonylamino)benzyl)ureido)acetate S1**

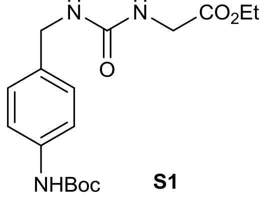

S1

Ethyl isocyanatoacetate (870 μl, 7.70 mmol) was dissolved in THF (0.4 M), then 4-(tert-butoxycarbonylamino)benzylamine (1.70 g, 7.70 mmol, 1.00 equiv) was added in one portion

and the reaction mixture was let 2 h at room temperature. The reaction mixture was concentrated and purified by precipitation in diethyl ether, yielding **S1** (2.69 g, 99%) as a white solid.

^{1}H-NMR (600 MHz): δ 9.27 (s, 1H), 7.37 (d, 2H, *J*= 8.4 Hz), 7.12 (d, 2H, *J*= 8.4 Hz), 6.58 (t, 1H, *J*= 5.8 Hz), 6.26 (t, 1H, *J* = 5.5 Hz), 4.13 (d, 2H, *J*= 5.5 Hz), 4.08 (q, 2H, *J*= 7.1 Hz), 3.78 (d, 2H, *J*= 5.8 Hz), 1.48 (s, 9H), 1.20 (t, 3H, *J*= 7.1 Hz). **^{13}C-NMR** (600 MHz): δ 171.6, 158.4, 153.2, 138.5, 134.6, 127.8, 118.4, 79.3, 60.6, 42.9, 42.1, 28.6, 14.6.

Synthesis of **ethyl 2-(3-(4-aminobenzyl)ureido)acetate 22**

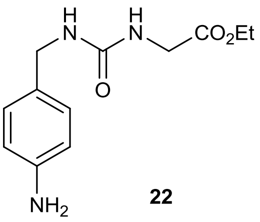

22

N-boc protected amine **S1** (200 mg, 0.57 mmol) was dissolved in 1 ml of CH_2Cl_2, then 1 ml of TFA was added and the reaction mixture was let 1 h at room temperature. The reaction mixture was concentrated and purified by precipitation using AcOEt/hexane to yield the deprotected amine **22** (TFA salt, 191 mg, 92%) as a yellow solid.

^{1}H-NMR (600 MHz): δ 6.90 (d, 2H, *J*= 8.4 Hz), 6.51 (d, 2H, *J*= 8.4 Hz), 6.40 (t, 1H, *J*= 6.0 Hz), 6.16 (t, 1H, *J* = 6.0 Hz), 4.92 (s, 2H), 4.10 (q, 2H, *J*= 7.2 Hz), 4.01 (d, 2H, *J*= 6.0 Hz), 3.78 (d, 2H, *J*= 6.0 Hz), 1.20 (t, 3H, *J*= 7.2 Hz). **^{13}C-NMR** (500 MHz): δ 171.7, 158.3, 147.8, 128.5, 127.8, 114.1, 60.6, 43.2, 42.0, 14.6. **ESI-MS** *m/z*: 252.2 $[M + H]^+$.

Synthesis of **2-(3-(4-(tert-butoxycarbonylamino)benzyl)ureido)acetic acid S2**

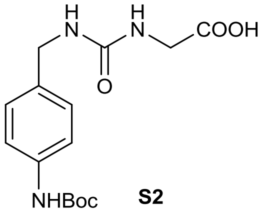

S2

Ester **S1** (2.00 g, 5.68 mmol) was dissolved in 20 ml of MeOH and 20 ml of water, LiOH (0.53 g, 22.72 mmol, 4.00 equiv) was added and the reaction mixture was heated to 50°C for 2 h. The reaction mixture was concentrated and 200 ml of water was added, then extracted twice with AcOEt. The aqueous phase was acidified to pH 3 with concentrated HCl 36%, then extracted twice with AcOEt. The combined organic phase was dried over Na_2SO_4, filtered and concentrated to yield **S2** (1.75 g, 96%) as a white solid.

^{1}H-NMR (200 MHz): δ 12.21 (s, 1H), 9.26 (s, 1H), 7.37 (d, 2H, *J*= 8.5 Hz), 7.13 (d, 2H, *J*= 8.5 Hz), 6.54 (t, 1H, *J*= 5.7 Hz), 6.14 (t, 1H, *J* = 5.6 Hz), 4.13 (d, 2H, *J*= 5.6 Hz), 3.72 (d, 2H, *J*= 5.7 Hz), 1.47 (s, 9H).

Synthesis of **tert-butyl 4-((3-(2-oxo-2-(pyrrolidin-1-yl)ethyl)ureido)methyl)phenylcarbamate S3**

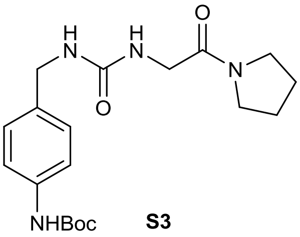

S3

Acid derivative **S2** (100 mg, 0.31 mmol) was dissolved in 2 ml of CH_2Cl_2. Pyrrolidine (28.5 µl, 1.10 equiv), hydroxybenzotriazole (46 mg, 1.1 equiv), diisopropylethylamine (119 µl, 2.2 equiv) and 1-[3-(dimethylamino)propyl]-3-ethylcarbodiimide (60 µl, 1.1 equiv) were added successively and the reaction mixture was stirred for 20 h at room temperature. The reaction mixture was concentrated and 100 ml of AcOEt was added. The organic phase was washed with saturated $NaHCO_3$, 10% citric acid and brine, then dried over Na_2SO_4, filtered and

concentrated. Purification by silica flash chromatography (100% EtOAc) yielded the amide **S3** (51 mg; 44%) as a white solid.

TLC: Rf 0.09 (EtOAc). **^{1}H-NMR** (500 MHz): δ 9.27 (s, 1H), 7.37 (d, 2H, *J* = 8.5 Hz), 7.12 (d, 2H, *J* = 8.5 Hz), 6.66 (t, 1H, *J* = 5.8 Hz), 6.06 (t, 1H, *J* = 4.9 Hz), 4.12 (d, 2H, *J* = 5.8 Hz), 3.81 (d, 2H, *J* = 4.9 Hz), 3.30 (m, 4H), 1.90 (m, 4H), 1.47 (s, 9H). **^{13}C-NMR** (500 MHz): δ 168.1, 158.4, 153.2, 138.5, 134.6, 120.5, 117.4, 79.3, 46.0, 45.2, 43.0, 42.7, 26.1, 24.2. **ESI-MS** *m/z*: 377.3 $[M + H]^+$.

Synthesis of **1-(4-aminobenzyl)-3-(2-oxo-2-(pyrrolidin-1-yl)ethyl)urea 23**

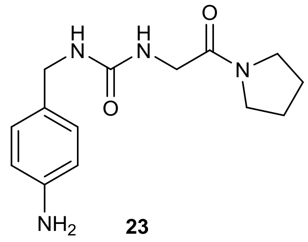

23

N-boc protected amine **S3** (50 mg, 0.13 mmol) was dissolved in 0.5 ml of CH_2Cl_2, then 0.5 ml of TFA was added and the reaction mixture was let 1 h at room temperature. The reaction mixture was concentrated and purified by precipitation using AcOEt/hexane to yield the deprotected amine **23** (TFA salt, 36 mg, 70%) as a yellow solid.

^{1}H-NMR (500 MHz): δ 6.90 (d, 2H, *J*= 8.4 Hz), 6.51 (d, 2H, *J*= 8.4 Hz), 6.40 (t, 1H, *J*= 6.0 Hz), 6.16 (t, 1H, *J* = 6.0 Hz), 4.92 (s, 2H), 3.82-3.75 (m, 4H), 1.92-1.84 (m, 4H). **^{13}C-NMR** (500 MHz): δ 168.0, 158.4, 147.9, 128.6, 120.0, 113.2, 46.0, 45.2, 43.0, 42.7, 26.1, 24.2. **ESI-MS** *m/z*: 277.2 $[M + H]^+$.

Synthesis of **tert-butyl 4-((3-(2-(2-(2-methoxyphenyl)pyrrolidin-1-yl)-2-oxoethyl)ureido)methyl)phenylcarbamate S4**

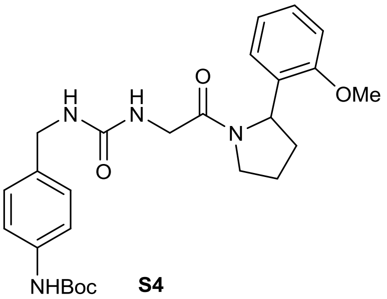

S4

Acid derivative **S2** (100 mg, 0.31 mmol) was dissolved in 2 ml of CH_2Cl_2. O-methoxy-2-phenyl-pyrrolidine (60 mg, 1.10 equiv), hydroxybenzotriazole (46 mg, 1.1 equiv), diisopropylethylamine (119 µl, 2.2 equiv) and 1-[3-(dimethylamino)propyl]-3-ethylcarbodiimide (60 µl, 1.1 equiv) were added successively and the reaction mixture was stirred for 20 h at room temperature. The reaction mixture was concentrated and 100 ml of AcOEt was added. The organic phase was washed with saturated $NaHCO_3$, 10% citric acid and brine, then dried over Na_2SO_4, filtered and concentrated. Purification by silica flash chromatography (EtOAc/MeOH 9/1) yielded the amide **S4** (131 mg; 88%) as a white solid.

^{1}H-NMR (500 MHz): δ 9.27 (s, 1H), 7.33-6.97 (m, 8H), 6.88-6.76 (m, 1H), 6.68-6.54 (m, 1H), 5.58-5.06 (m, 1H), 4.51 (s, 3H), 4.29-4.16 (m, 2H), 3.84-3.14 (m, 4H), 2.34-2.09 (m, 1H), 2-1.63 (m, 3H), 1.48 (s, 9H). **^{13}C-NMR** (500 MHz): δ 168.0, 158.3, 153.2, 138.9, 133.3, 128.7, 128.4, 127.7, 126.2, 125.9, 125.5, 125.1, 125.0, 120.8, 79.4, 65.4, 58.4, 57.8, 47.5, 42.8, 42.4, 34.1, 32.1, 28.4, 23.5, 21.5, 15.5. **ESI-MS** *m/z*: 483.3 [M + H]$^+$.

Synthesis of **1-(4-aminobenzyl)-3-(2-(2-(2-methoxyphenyl)pyrrolidin-1-yl)-2-oxoethyl)urea 24**

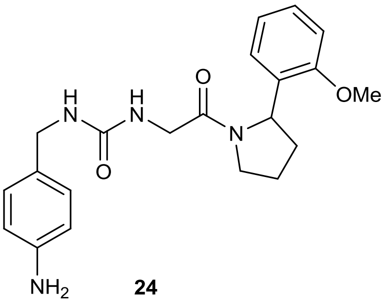

24

N-boc protected amine **S4** (124 mg, 0.26 mmol) was dissolved in 1 ml of CH_2Cl_2, then 1 ml of TFA was added and the reaction mixture was let 1 h at room temperature. The reaction mixture was concentrated and purified by precipitation using AcOEt/hexane to yield the deprotected amine **24** (TFA salt, 112 mg, 88%) as a yellow solid.

^{1}H-NMR (500 MHz): δ δ 7.32 (m, 3H), 7.20 (m, 5H), 7.04 (m, 6H), 6.69 (s, 1H), 6.03 (s, 1H), 5.23 (m, 2H), 4.55 (s, 2H), 4.17 (s, 2H), 4.10 (s, 2H), 3.98 (m, 4H), 3.81 (m, 3H), 3.71 (m, 4H), 2.35 (m, 2H), 2.18 (m, 1H), 2.00 (s, 1H), 1.92 (s, 2H), 1.75 (m, 5H), 1.47 (s, 9H). **^{13}C-NMR** (500 MHz) : δ 168.0, 158.3, 140.9, 135.3, 128.7, 128.4, 127.7, 126.2, 125.9, 125.5, 125.1, 125.0, 120.8, 58.4, 57.8, 47.5, 46.4, 42.8, 42.4, 34.1, 32.1, 23.5, 21.5, 15.5. **ESI-MS** m/z: 383.4 [M + H]$^+$.

Synthesis of **tert-butyl 4-((3-(2-(2-(2-chlorophenyl)pyrrolidin-1-yl)-2-oxoethyl)ureido)methyl)phenylcarbamate S5**

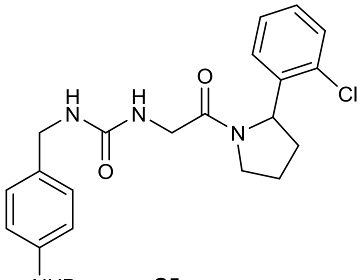

S5

Acid derivative **S2** (100 mg, 0.31 mmol) was dissolved in 2 ml of CH_2Cl_2. o-chloro-2-phenyl-pyrrolidine (62 mg, 1.10 equiv), hydroxybenzotriazole (46 mg, 1.1 equiv), diisopropylethylamine (119 µl, 2.2 equiv) and 1-[3-(dimethylamino)propyl]-3-ethylcarbodiimide (60 µl , 1.1 equiv) were added successively and the reaction mixture was stirred for 20 h at room temperature. The reaction mixture was concentrated and 100 ml of AcOEt was added. The organic phase was washed with saturated $NaHCO_3$, 10% citric acid and brine, then dried over Na_2SO_4, filtered and concentrated. Purification by silica flash chromatography (95/5 EtOAc/MeOH) yielded the amide **S5** (96 mg, 64%) as a white solid.

TLC: R*f*=0.26 (EtOAc). **^{1}H NMR** (300 MHz):δ 7.43-6.80 (m, 8H), 6.67 (s, 1H), 5.42-5.13 (m, 1H), 4.23-4.16 (m, 2H), 4.16-4.02 (m, 1H), 3.79-3.20 (m, 3H), 2.40-2.07 (m, 1H), 2.00-1.53 (m, 4H), 1.50-1.46 (m, 9H).

Synthesis of **1-(4-aminobenzyl)-3-(2-(2-(2-chlorophenyl)pyrrolidin-1-yl)-2-oxoethyl)urea 25**

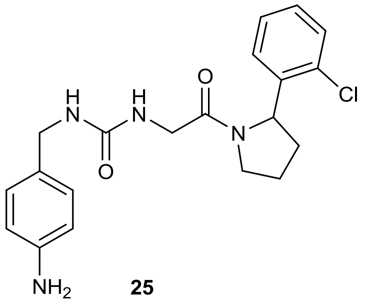

25

N-boc protected amine **S5** (95 mg, 0.19 mmol) was dissolved in 1 ml of CH_2Cl_2, then 1 ml of TFA was added and the reaction mixture was let 1 h at room temperature. The reaction mixture was concentrated and purified by precipitation using AcOEt/hexane to yield the deprotected amine **25** (TFA salt, 66 mg, 90%) as a yellow solid.

TLC: R*f* 0.37 (CH_2Cl_2/MeOH 95/5). **^{1}H-NMR** (500 MHz): δ 9.27 (s, 1H), 7.43-6.80 (m, 8H), 6.67 (s, 1H), 5.42-5.13 (m, 1H), 4.23-4.16 (m, 2H), 4.16-4.02 (m, 1H), 3.79-3.20 (m, 3H), 2.40-2.07 (m, 1H), 2.00-1.53 (m, 4H), 1.46 (S, 9H). **^{13}C-NMR** (500 MHz): δ 168.7, 168.3, 158.5, 158.3, 158.2, 158.1, 140.9, 140.4, 131.4, 131.3, 130.3, 129.8, 129.5, 128.7, 128.6, 128.2, 127.4, 127.2, 127.1, 119.0, 58.5, 58.0, 47.5, 46.5, 43.0, 42.9, 42.8, 42.4, 34.3, 32.2, 23.4, 21.3. **ESI-MS** *m/z*: 486.6/488.6 $[M + H]^+$.

Synthesis of **tert-butyl 4-((3-(2-(2-(2-bromophenyl)pyrrolidin-1-yl)-2-oxoethyl)ureido)methyl)phenylcarbamate S6**

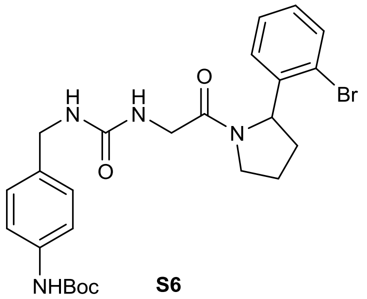

S6

Acid derivative **S2** (100 mg, 0.31 mmol) was dissolved in 2 ml of CH_2Cl_2. o-bromo-2-phenyl-pyrrolidine (77 mg, 1.10 equiv), hydroxybenzotriazole (46 mg, 1.1 equiv), diisopropylethylamine (119 µl, 2.2 equiv) and 1-[3-(dimethylamino)propyl]-3-ethylcarbodiimide (60 µl , 1.1 equiv) were added successively and the reaction mixture was stirred for 20 h at room temperature. The reaction mixture was concentrated and 100 ml of AcOEt was added. The organic phase was washed with saturated $NaHCO_3$, 10% citric acid and brine, then dried over Na_2SO_4, filtered and concentrated. Purification by silica flash chromatography (95/5 EtOAc/MeOH) yielded the amide **S5** (69 mg, 42%) as a white solid.

TLC: Rf 0.28 (EtOAc). **^{1}H-NMR** (300 MHz): δ 9.27 (s, 1H), 7.58-7.45 (m, 1H), 7.35-6.98 (m, 6H), 6.92-6.86 (m, 1H), 6.54 (s, 1H), 5.98 (t, *J* = 4.3 Hz, 1H), 5.86 (t, *J* = 4.4 Hz, 1H), 5.67-5.53 (m, 1H), 5.41-5.09 (m, 1H), 4.27-4.18 (m, 2H), 4.17-3.23 (m, 4H), 2.44-2.18 (m, 1H), 2.01-1.54 (m, 3H), 1.49 (s, 9H). **^{13}C-NMR** (500 MHz): δ 168.2, 158.2, 153.1, 138.3, 133.5, 133.1, 129.8, 129.0, 128.7, 128.0, 127.2, 121.9, 120.4, 79.4, 60.7, 60.3, 47.7, 46.6, 42.9, 42.8, 34.5, 32.4, 28.6, 23.3, 21.2. **ESI-MS** *m/z*: 531.3/533.3 $[M + H]^+$.

Synthesis of **1-(4-aminobenzyl)-3-(2-(2-(2-bromophenyl)pyrrolidin-1-yl)-2-oxoethyl)urea 26**

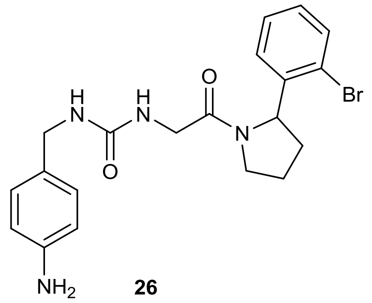

NH2 **26**

N-boc protected amine **S5** (69 mg, 0.13 mmol) was dissolved in 1 ml of CH_2Cl_2, then 1 ml of TFA was added and the reaction mixture was let 1 h at room temperature. The reaction mixture was concentrated and purified by precipitation using diethylether to yield the deprotected amine **26** (TFA salt, 29 mg, 51%) as a brown solid.

TLC: Rf 0.08 (EtOAc). **^{1}H-NMR** (500 MHz) : δ 7.68 (d, *J* = 8.0 Hz, 1H), 7.61 (d, *J* = 8.0 Hz, 1H), 7.41 (7, *J* = 7.3 Hz, 1H), 7.29 (m, 2H), 7.17 (m, 7H), 7.03 (3, 4H), 6.68 (s, 1H), 6.05 (s, 1H), 5.22 (m, 2H), 4.16 (s, 2H), 4.09 (s, 2H), 3.97 (m, 4H), 3.85 (m, 4H), 3.74 (m, 3H), 3.59 (m, 7H), 2.27 (m, 2H), 1.93 (m, 2H), 1.81 (m, 3H), 1.70 (m, 2H). **^{13}C-NMR** (500 MHz) : δ 168.3, 158.3, 142.3, 133.5, 133.1, 129.8, 129.0, 128.7, 128.0, 127.2, 121.9, 120.4, 60.7, 60.3, 47.7, 46.6, 42.9, 42.8, 34.5, 32.4, 23.3, 21.2. **ESI-MS** *m/z*: 431.2/433.2 [M + H]$^+$.

Synthesis of **tert-butyl 4-((3-(2-(2-(2-(methylthio)phenyl)pyrrolidin-1-yl)-2-oxoethyl)ureido)methyl)phenylcarbamate S7**

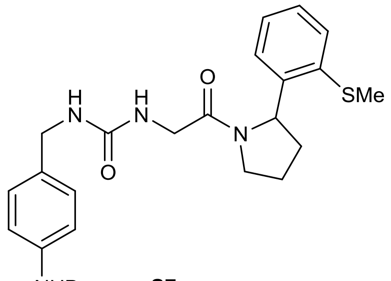

NHBoc **S7**

Acid derivative **S2** (100 mg, 0.31 mmol) was dissolved in 2 ml of CH_2Cl_2. o-thiomethyl-2-phenyl-pyrrolidine (66 mg, 1.10 equiv), hydroxybenzotriazole (46 mg, 1.1 equiv), diisopropylethylamine (119 μl, 2.2 equiv) and 1-[3-(dimethylamino)propyl]-3-

ethylcarbodiimide (60 µl, 1.1 equiv) were added successively and the reaction mixture was stirred for 20 h at room temperature. The reaction mixture was concentrated and 100 ml of AcOEt was added. The organic phase was washed with saturated $NaHCO_3$, 10% citric acid and brine, then dried over Na_2SO_4, filtered and concentrated. Purification by silica flash chromatography (100% EtOAc) yielded the amide **S5** (95 mg, 62%) as a white solid.

TLC: Rf 0.38 (EtOAc). **^{1}H-NMR** (500 MHz): δ 9.27 (s, 1H), 7.33-6.97 (m, 8H), 6.88-6.76 (m, 1H), 6.68-6.54 (m, 1H), 5.58-5.06 (m, 1H), 4.29-4.16 (m, 2H), 3.84-3.14 (m, 4H), 2.52-2.35 (m, 3H), 2.34-2.09 (m, 1H), 2-1.63 (m, 3H), 1.48 (s, 9H). **^{13}C-NMR** (500 MHz): δ 168.0, 158.3, 153.2, 138.9, 133.3, 128.7, 128.4, 127.7, 126.2, 125.9, 125.5, 125.1, 125.0, 120.8, 79.4, 58.4, 57.8, 47.5, 46.4, 42.8, 42.4, 34.1, 32.1, 28.4, 23.5, 21.5, 15.5. **ESI-MS** *m/z*: 499.3 $[M + H]^+$.

Synthesis of **1-(4-aminobenzyl)-3-(2-(2-(2-(methylthio)phenyl)pyrrolidin-1-yl)-2-oxoethyl)urea 27**

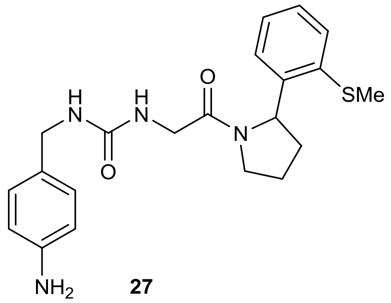

27

N-boc protected amine **S7** (95 mg, 0.19 mmol) was dissolved in 1 ml of CH_2Cl_2, then 1 ml of TFA was added and the reaction mixture was let 1 h at room temperature. The reaction mixture was concentrated and purified by precipitation using diethylether to yield the deprotected amine **27** (TFA salt, 60 mg, 80%) as a yellow solid.

TLC: Rf 0.32 (95:5 CH_2Cl_2/MeOH). **^{1}H-NMR** (500 MHz): δ 7.32 (m, 3H), 7.20 (m, 5H), 7.04 (m, 6H), 6.69 (s, 1H), 6.03 (s, 1H), 5.23 (m, 2H), 4.17 (s, 2H), 4.10 (s, 2H), 3.98 (m, 4H), 3.81 (m, 3H), 3.71 (m, 4H), 3.56 (m, 4H), 2.35 (m, 2H), 2.18 (m, 1H), 2.00 (s, 1H), 1.92 (s, 2H), 1.75 (m,

5H), 1.47 (s, 9H). **^{13}C-NMR** (500 MHz) : δ 168.0, 158.3, 140.9, 135.3, 128.7, 128.4, 127.7, 126.2, 125.9, 125.5, 125.1, 125.0, 120.8, 58.4, 57.8, 47.5, 46.4, 42.8, 42.4, 34.1, 32.1, 23.5, 21.5, 15.5. **ESI-MS** *m/z*: 399.2 $[M + H]^+$.

Synthesis of **tert-butyl 4-((3-(2-(2-(naphthalen-1-yl)pyrrolidin-1-yl)-2-oxoethyl)ureido)methyl)phenylcarbamate S8**

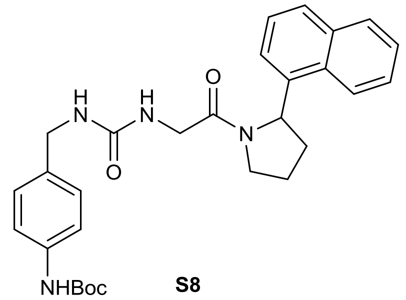

S8

Acid derivative **S2** (100 mg, 0.31 mmol) was dissolved in 2 ml of CH_2Cl_2. 1-naphtyl-2-phenyl-pyrrolidine (67 mg, 1.10 equiv), hydroxybenzotriazole (46 mg, 1.1 equiv), diisopropylethylamine (119 µl , 2.2 equiv) and 1-[3-(dimethylamino)propyl]-3-ethylcarbodiimide (60 µl , 1.1 equiv) were added successively and the reaction mixture was stirred for 20 h at room temperature. The reaction mixture was concentrated and 100 ml of AcOEt was added. The organic phase was washed with saturated $NaHCO_3$, 10% citric acid and brine, then dried over Na_2SO_4, filtered and concentrated. Purification by silica flash chromatography (100% EtOAc) yielded the amide **S8** (71 mg, 46%) as a white solid.

TLC: Rf 0.16 (EtOAc). **^{1}H-NMR** (500 MHz): δ 9.28 (s, 1H), 8.17 (d, *J* = 8.0 Hz, 2H), 8.13 (d, *J* = 8.0 Hz, 2H), 7.98 (dd, *J* = 7.6; 6.8 Hz, 1H), 7.87 (d, *J* = 8.0 Hz, 1H), 7.80 (d, *J* = 7.6 Hz, 2H), 7.59 (m, 5H), 7.49 (t, *J* = 7.2 Hz, 1H), 7.41 (t, *J* = 7.6 Hz, 1H), 7.18 (m, 5H), 7.12 (d, *J* = 7.0 Hz, 2H), 6.98 (m, 4H), 6.66 (m, 1H), 5.91 (d, *J* = 7.6 Hz, 2H), 5.82 (d, *J* = 7.0 Hz, 2H), 4.16 (s, 4H), 4.05 (m, 7H), 3.91 (m, 4H), 3.80 (m, 2H), 3.64 (m, 4H), 3.18 (m, 2H), 2.45 (m, 2H), 1.97 (m, 2H), 1.79 (m, 9H). **^{13}C-NMR** (500 MHz) : δ 168.7, 168.2, 158.4, 158.1, 153.2, 138.8, 138.7, 134.1,

134.0, 130.3, 130.0, 129.3, 129.1, 128.6, 128.1, 127.4, 126.9, 126.5, 126.4, 126.0, 125.9, 125.7, 123.9, 123.6, 122.2, 120.0, 70.3, 58.0, 57.4, 47.4, 46.4, 42.9, 42.8, 42.5, 34.8, 32.8, 23.7, 21.6, 20.4. **ESI-MS** *m/z*: 503.3 $[M + H]^+$.

Synthesis of **1-(4-aminobenzyl)-3-(2-(2-(naphthalen-1-yl)pyrrolidin-1-yl)-2-oxoethyl)urea 28**

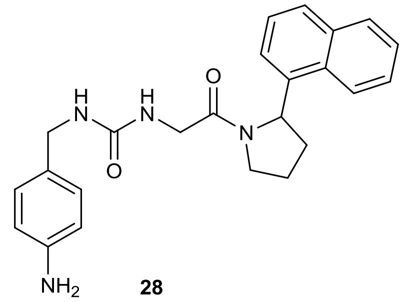

28

N-boc protected amine **S8** (71 mg, 0.14 mmol) was dissolved in 1 ml of CH_2Cl_2, then 1 ml of TFA was added and the reaction mixture was let 1 h at room temperature. The reaction mixture was concentrated and purified by precipitation using diethylether to yield the deprotected amine **28** (TFA salt, 29 mg, 52%) as a white solid.

^{1}H-NMR (500 MHz): δ 8.17 (d, *J* = 8.0 Hz, 2H), 8.13 (d, *J* = 8.0 Hz, 2H), 7.98 (dd, *J* = 7.6; 6.8 Hz, 1H), 7.87 (d, *J* = 8.0 Hz, 1H), 7.80 (d, *J* = 7.6 Hz, 2H), 7.59 (m, 5H), 7.49 (t, *J* = 7.2 Hz, 1H), 7.41 (t, *J* = 7.6 Hz, 1H), 7.18 (m, 5H), 7.12 (d, *J* = 7.0 Hz, 2H), 6.98 (m, 4H), 6.66 (m, 1H), 5.91 (d, *J* = 7.6 Hz, 2H), 5.82 (d, *J* = 7.0 Hz, 2H), 4.16 (s, 4H), 4.05 (m, 7H), 3.91 (m, 4H), 3.80 (m, 2H), 3.64 (m, 4H), 3.18 (m, 2H), 2.45 (m, 2H), 1.97 (m, 2H), 1.79 (m, 9H). **^{13}C-NMR** (500 MHz) : δ 168.7, 168.2, 158.4, 158.1, 138.8, 138.7, 134.1, 134.0, 130.3, 130.0, 129.3, 129.1, 128.6, 128.1, 127.4, 126.9, 126.5, 126.4, 126.0, 125.9, 125.7, 123.9, 123.6, 122.2, 120.0, 58.0, 57.4, 47.4, 46.4, 42.9, 42.8, 42.5, 34.8, 32.8, 23.7, 21.6. **ESI-MS** *m/z*: 403.2 $[M + H]^+$.

Scheme 2. Synthesis of compound **29: 1-(4-aminobenzyl)-3-(4-methyl-1-(2-(2-(methylthio)phenyl)pyrrolidin-1-yl)-1-oxopentan-2-yl)urea 29**

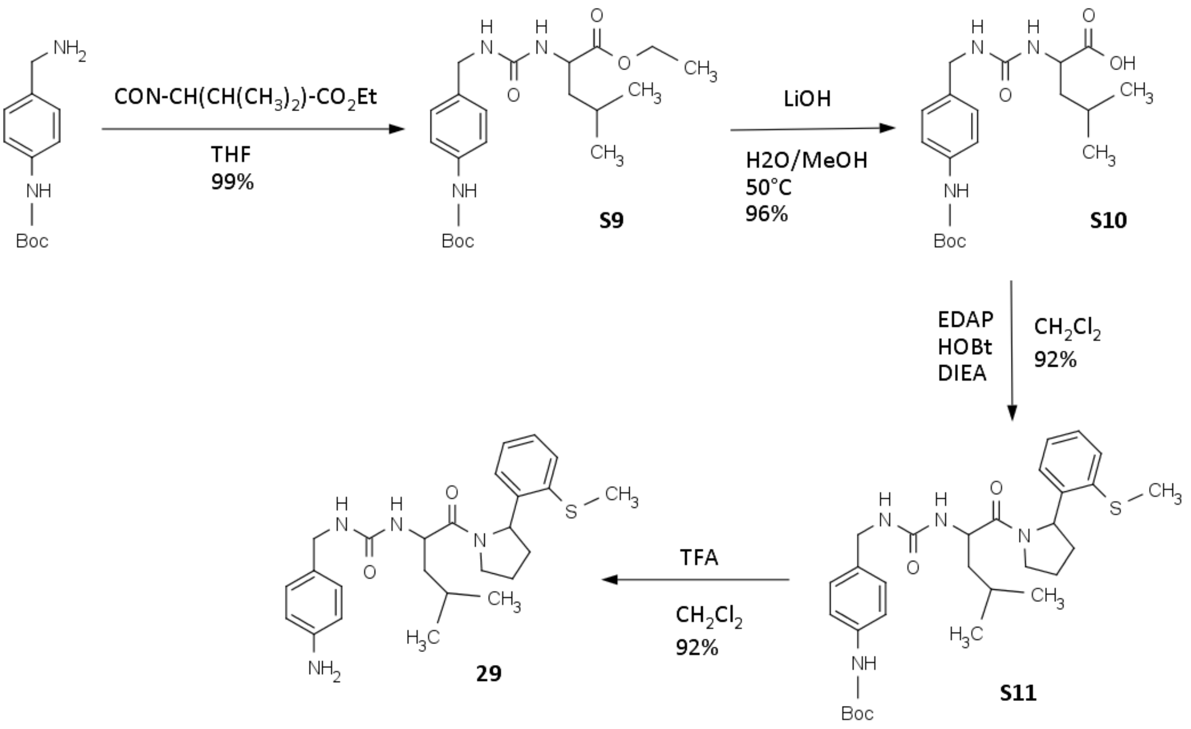

Synthesis of **ethyl 2-(3-(4-(tert-butoxycarbonylamino)benzyl)ureido)-4-methylpentanoate S9**

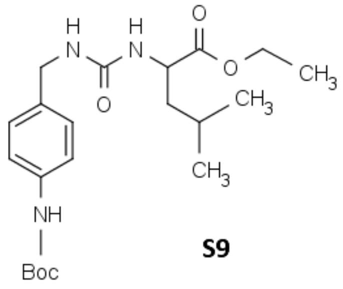

Ethyl 2-isocyanato-4-methylpentanoate (981 mg, 5.30 mmol, 1.20 equiv) was dissolved in THF (0.4 M), then 4-(tert-butoxycarbonylamino)benzylamine (982 mg, 4.41 mmol, 1.00 equiv) was added in one portion and the reaction mixture was let 2 h at room temperature.

The reaction mixture was concentrated and purified by precipitation in diethylether, yielding **S9** (1.78 g, 99%) as a yellow solid.

TLC: R*f* 0.48 (EDP/EtOAc 5/5). **^{1}H-NMR** (600 MHz): δ 9.27 (s, 1H), 7.28 (d, *J* = 8.6 Hz, 2H), 7.16 (d, *J* = 8.6 Hz, 2H), 6.60 (broad s, 1H), 5.17-5.01 (m, 2H), 4.55-4.38 (m, 1H), 4.26 (d, *J* = 5.6 Hz, 2H), 4.11 (q, *J* = 7.2 Hz, 2H), 1.79-1.33 (m, 3H), 1.50 (s, 9H), 1.23 (t, *J* = 7.2 Hz, 3H), 0.97-0.86 (m, 6H). **^{13}C-NMR** (600 MHz): δ 171.6, 158.4, 153.2, 138.5, 134.6, 127.8, 118.4, 79.3, 60.6, 42.9, 42.1, 28.6, 14.6. **ESI-MS** *m/z*: 408.3 [M + H]$^{+}$.

Synthesis of **2-(3-(4-(tert-butoxycarbonylamino)benzyl)ureido)-4-methylpentanoic acid S10**

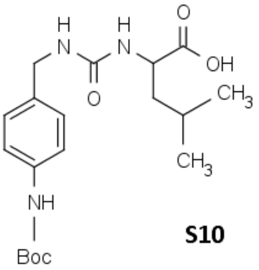

S10

Ester **S9** (1.78 g, 4.37 mmol) was dissolved in 20 ml of MeOH and 20 ml of water, LiOH (0.42 g, 17.48 mmol, 4.00 equiv) was added and the reaction mixture was heated to 50°C for 2 h. The reaction mixture was concentrated and 200 ml of water was added; it was then extracted twice with AcOEt. The aqueous phase was acidified to pH 3 with concentrated HCl 36%, then extracted twice with AcOEt. The combined organic phase was dried over Na_2SO_4, filtered and concentrated to yield **S10** (1.59 g, 96%) as a white solid.

^{1}H NMR (300 MHz): δ 9.31 (broad s, 1H), 7.41 (d, *J* = 8.4 Hz, 2H), 7.15 (d, *J* = 8.4 Hz, 2H), 6.60 (t, *J* = 4.9 Hz, 1H), 6.06 (d, *J* = 7.2 Hz, 1H), 4.18-4.11 (m, 2H), 3.99-3.85 (m, 1H), 1.80-1.27 (m, 3H), 1.51 (s, 9H), 0.95-0.87 (m, 6H). **ESI-MS** *m/z*: 379.3 [M - H]$^{-}$.

Synthesis of **tert-butyl 4-((3-(4-methyl-1-(2-(2-(methylthio)phenyl)pyrrolidin-1-yl)-1-oxopentan-2-yl)ureido)methyl)phenylcarbamate S11**

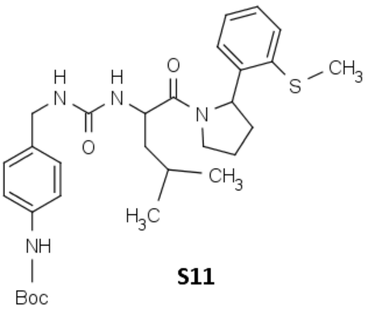

S11

Acid derivative **S10** (498 mg, 1.31 mmol) was dissolved in 10 ml of CH_2Cl_2. o-thiomethyl-2-phenyl-pyrrolidine (279 mg, 1.10 equiv), hydroxybenzotriazole (212 mg, 1.2 equiv), diisopropylethylamine (497 µl, 2.2 equiv) and 1-[3-(dimethylamino)propyl]-3-ethylcarbodiimide (300 mg, 1.2 equiv) were added successively and the reaction mixture was stirred for 20 h at room temperature. The reaction mixture was concentrated and 100 ml of AcOEt was added. The organic phase was washed with saturated $NaHCO_3$, 10% citric acid and brine, then dried over Na_2SO_4, filtered and concentrated. Purification by silica flash chromatography (EDP/EtOAc 5/5) yielded the amide **S11** (667 mg, 92%) as a white solid.

TLC: Rf=0.17 (EDP/EtOAc 3/7). **^{1}H-NMR** (500 MHz): δ 9.27 (s, 1H), 7.33-7.19 (m, 8H), 6.58 (m, 1H), 6.04 (m, 1H), 4.98 (m, 1H), 4.42 (s, 2H), 4.38 (m, 1H), 3.50 (m, 2H), 2.48 (m, 3H), 2.21 (m, 1H), 2.03 (m, 2H), 1.83-1.79 (m, 3H), 1.56 (m, 1H), 1.43 (s, 9H), 0.91 (m, 6H) . **^{13}C-NMR** (500 MHz): δ 173.3, 171.6, 158.2, 158.1, 153.2, 147.8, 141.5, 140.9, 135.7, 135.2, 128.5, 128.3, 128.0, 127.8, 127.5, 126.5, 126.2, 125.9, 125.1, 125.0, 124.9, 114.2, 79.3, 58.5, 58.2, 49.7, 49.3, 47.3, 47.2, 43.2, 41.4, 41.3, 34.0, 32.1, 28.6, 24.7, 23.9, 23.7, 23.3, 22.3, 21.6, 20.3, 15.8, 15.6. **ESI-MS** *m/z*: 555.3 $[M + H]^+$.

Synthesis of **1-(4-aminobenzyl)-3-(4-methyl-1-(2-(2-(methylthio)phenyl)pyrrolidin-1-yl)-1-oxopentan-2-yl)urea 29**

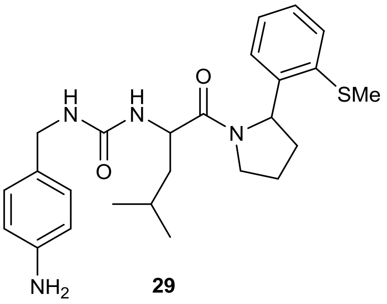

29

N-boc protected amine **S11** (660 mg, 1.19 mmol) was dissolved in 3 ml of CH_2Cl_2, then 3 ml of TFA was added and the reaction mixture was let 1 h at room temperature. The reaction mixture was concentrated and purified by precipitation using diethylether to yield the deprotected amine **29** (TFA salt, 614 mg, 92%) as a yellow solid.

TLC: Rf=0.64 (MeOH). **^{1}H-NMR** (500 MHz): δ 7.32-7.15 (m, 8H), 6.48 (m, 1H), 6.00 (m, 1H), 4.94 (m, 1H), 4.40 (s, 2H), 4.38 (m, 1H), 3.50 (m, 2H), 2.48 (m, 3H), 2.21 (m, 1H), 2.03 (m, 2H), 1.83-1.79 (m, 3H), 1.56 (m, 1H), 0.91 (m, 6H). **^{13}C-NMR** (500 MHz): δ 173.3, 171.6, 158.2, 158.1, 147.8, 141.5, 140.9, 135.7, 135.2, 128.5, 128.3, 128.0, 127.8, 127.5, 126.5, 126.2, 125.9, 125.1, 125.0, 124.9, 114.2, 58.5, 58.2, 49.7, 49.3, 47.3, 47.2, 43.2, 41.4, 41.3, 34.0, 32.1, 24.7, 23.9, 23.7, 23.3, 22.3, 21.6, 20.3, 15.8, 15.6. **ESI-MS** *m/z*: 455.2 $[M + H]^+$.

Scheme 3. Synthesis of compound **30**: **1-(4-aminobenzyl)-3-(4-(methylthio)-1-(2-(2-(methylthio)phenyl)pyrrolidin-1-yl)-1-oxobutan-2-yl)urea 30**

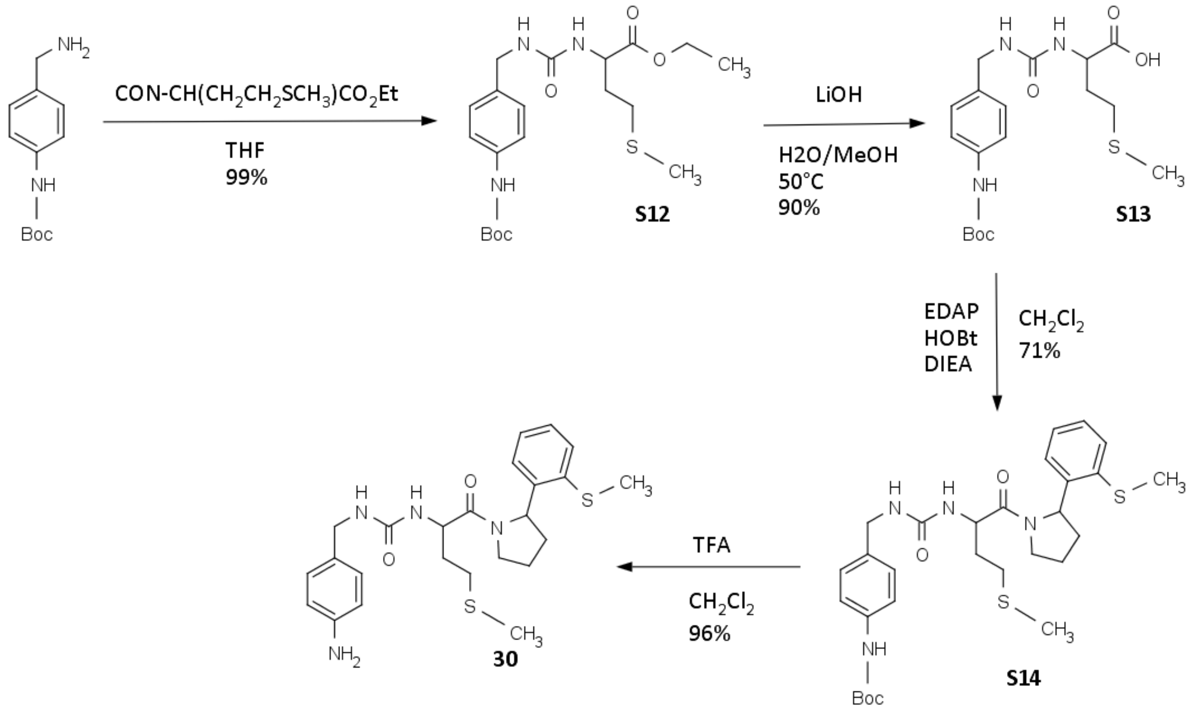

Synthesis of **ethyl 2-(3-(4-(tert-butoxycarbonylamino)benzyl)ureido)-4-(methylthio)butanoate S12**

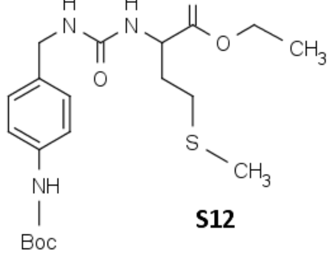

Ethyl 2-isocyanato-4-(methylsulfanyl)butanoate (836 mg, 4.12 mmol) was dissolved in THF (0.4 M), then 4-(tert-butoxycarbonylamino)benzylamine (911 mg, 1.00 equiv) was added in one portion and the reaction mixture was let 2 h at room temperature. The reaction mixture

was concentrated and purified by precipitation in diethyl ether, yielding **S12** (1.74 g, 99%) as a yellow solid.

TLC: R*f* 0.42 (EDP/EtOAc 5/5). **^{1}H-NMR** (600 MHz): δ 9.26 (s, 1H), 7.29 (d, *J* = 8.2 Hz, 2H), 7.17 (d, *J* = 8.2 Hz, 2H), 6.60 (s, 1H), 6.05 (s, 1H), 4.42 (s, 2H), 4.34 (t, *J* = 7.2 Hz, 1H), 4.14 (q, *J* = 7.1 Hz, 2H), 2.64 (m, 2H), 2.20 (s, 3H), 2.11 (m, 2H), 1.43 (s, 9H), 1.18 (t, *J* = 7.2 Hz, 3H). **^{13}C-NMR** (600 MHz): δ 171.6, 158.4, 153.2, 138.5, 134.6, 127.8, 118.4, 79.3, 60.6, 55.0, 50.2, 42.9, 26.8, 14.6, 10.6. **ESI-MS** *m/z*: 425.3 $[M + H]^+$.

Synthesis of **2-(3-(4-(tert-butoxycarbonylamino)benzyl)ureido)-4-(methylthio)butanoic acid S13**

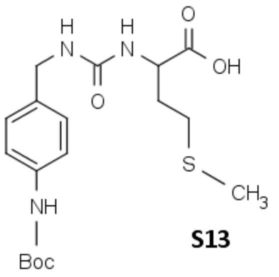

S13

Ester **S12** (1.60 g, 3.77 mmol) was dissolved in 10 ml of MeOH and 10 ml of water, LiOH (0.36 g, 15.10 mmol, 4.00 equiv) was added and the reaction mixture was heated to 50°C for 2 h. The reaction mixture was concentrated and 200 ml of water was added, then extracted twice with AcOEt. The aqueous phase was acidified to pH 3 with concentrated HCl 36%, then extracted twice with AcOEt. The combined organic phase was dried over Na_2SO_4, filtered and concentrated to yield **S13** (1.49 g, 90%) as a white solid.

^{1}H-NMR (200 MHz): δ 12.63 (broad s, 1H), 9.30 (s, 1H), 7.41 (d, *J* = 8.4 Hz, 2H), 7.15 (d, *J* = 8.4 Hz, 2H), 6.42 (t, *J* = 5.9 Hz, 1H), 6.30 (d, *J* = 8.5 Hz, 1H), 4.33-4.20 (m, 1H), 4.16 (d, *J* = 5.8 Hz, 2H), 2.54-2.44 (m, 2H), 2.08 (s, 3H), 2.05-1.70 (m, 2H), 1.50 (s, 9H). **ESI-MS** *m/z*: 395.2 $[M - H]^-$.

Synthesis of **tert-butyl 4-((3-(4-(methylthio)-1-(2-(2-(methylthio)phenyl)pyrrolidin-1-yl)-1-oxobutan-2-yl)ureido)methyl)phenylcarbamate S14**

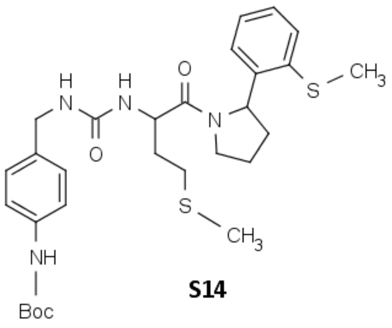

S14

Acid derivative **S13** (1.13 g, 2.84 mmol) was dissolved in 15 ml of CH_2Cl_2. o-thiomethyl-2-phenyl-pyrrolidine (605 mg, 1.10 equiv), hydroxybenzotriazole (462 mg, 1.2 equiv), diisopropylethylamine (1.08 ml, 2.2 equiv) and 1-[3-(dimethylamino)propyl]-3-ethylcarbodiimide (650 mg, 1.2 equiv) were added successively and the reaction mixture was stirred for 20 h at room temperature. The reaction mixture was concentrated and 100 ml of AcOEt was added. The organic phase was washed with saturated $NaHCO_3$, 10% citric acid and brine, then dried over Na_2SO_4, filtered and concentrated. Purification by silica flash chromatography (EDP/EtOAc 5/5) yielded the amide **S14** (1.15 g, 71%) as a white solid.

TLC: Rf=0.15 (EDP/EtOAc 3/7). **^{1}H-NMR** (500 MHz): δ 9.27 (s, 1H), 7.32-7.19 (m, 8H), 6.61 (s, 1H), 6.05 (s, 1H), 4.98 (m, 1H), 4.41 (m, 3H), 3.56 (m, 1H), 3.49 (m, 1H), 2.66 (t, *J* = 7.3 Hz, 2H), 2.48 (s, 3H), 2.20 (m, 6H), 2.03 (m, 2H), 1.89 (m, 1H), 1.43 (s, 9H). **^{13}C-NMR** (500 MHz): δ 173.3, 171.6, 158.2, 158.1, 147.8, 141.5, 140.9, 135.7, 135.2, 128.5, 128.3, 128.0, 127.8, 127.5, 126.5, 126.2, 125.9, 125.1, 125.0, 124.9, 114.2, 58.5, 58.2, 49.7, 49.3, 47.3, 47.2, 43.2, 41.4, 41.3, 34.0, 32.1, 24.7, 23.9, 23.7, 23.3, 22.3, 21.6, 20.3, 15.8, 15.6. **ESI-MS** *m/z*: 572.3 [M + H]$^+$.

Synthesis of **1-(4-aminobenzyl)-3-(4-(methylthio)-1-(2-(2-(methylthio)phenyl)pyrrolidin-1-yl)-1-oxobutan-2-yl)urea 30**

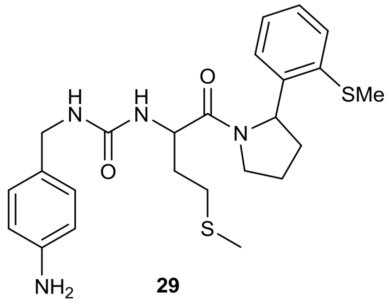

29

N-boc protected amine **S14** (1.10 g, 1.92 mmol) was dissolved in 5 ml of CH_2Cl_2, then 5 ml of TFA was added and the reaction mixture was let 1 h at room temperature. The reaction mixture was concentrated and purified by precipitation using diethylether/EtOAc to yield the deprotected amine **30** (TFA salt, 1.07 g, 96%) as a yellow solid.

TLC: Rf=0.71 (MeOH). **^{1}H-NMR** (500 MHz): δ 7.32-7.19 (m, 8H), 6.61 (s, 1H), 6.05 (s, 1H), 4.98 (m, 1H), 4.41 (m, 3H), 3.56 (m, 1H), 3.49 (m, 1H), 2.66 (t, *J* = 7.3 Hz, 2H), 2.48 (s, 3H), 2.20 (m, 6H), 2.03 (m, 2H), 1.89 (m, 1H). **^{13}C-NMR** (500 MHz): δ 172.3, 170.9, 158.2, 158.1, 147.9, 141.5, 140.8, 135.6, 135.2, 128.5, 128.4, 127.8, 127.6, 127.5, 126.7, 126.1, 125.8, 125.3, 124.3, 114.2, 58.6, 58.2, 50.5, 50.4, 47.4, 47.3, 43.2, 34.0, 32.4, 32.1, 32.0, 30.1, 30.0, 23.3, 21.6, 15.9, 15.5, 15.2, 14.8. **ESI-MS** *m/z*: 472.2 [M + H]$^+$.

Scheme 4. Synthesis of compound **31: 1-(4-aminobenzyl)-3-(2-(2-(2-(methylthio)phenyl)pyrrolidin-1-yl)-2-oxo-1-phenylethyl)urea 31**

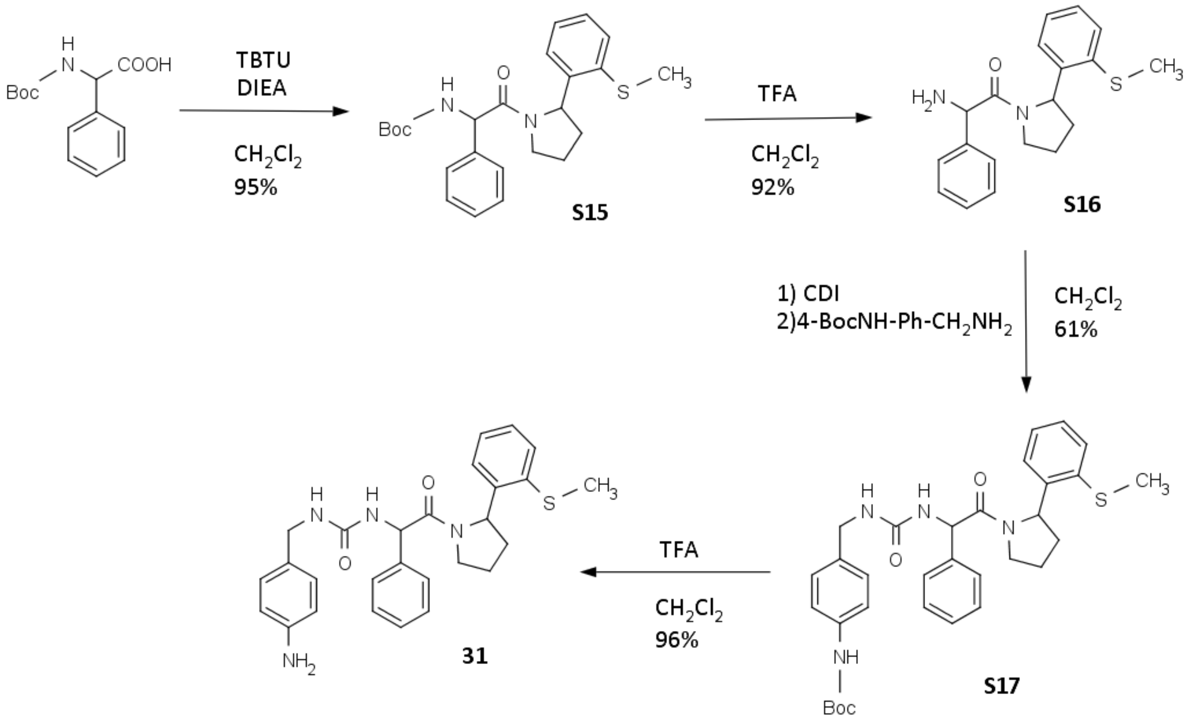

Synthesis of **tert-butyl N-(2-{2-[2-(methylsulfanyl)phenyl]pyrrolidin-1-yl}-2-oxo-1-phenylethyl)carbamate S15**

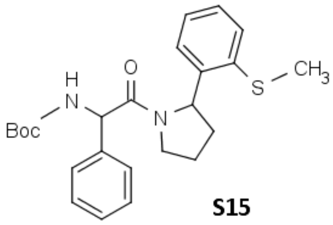

S15

Boc-phenylglycine (2.0 g, 7.96 mmol), TBTU (3.06 g, 1.20 equiv), o-thiomethyl-2-phenyl-pyrrolidine (1.85 g, 1.20 equiv), diisopropylethylamine (8.3 ml, 5.00 equiv) were dissolved in CH_2Cl_2 (50 mL) and stirred at room temperature for 60 h. The reaction mixture was concentrated and 100 ml of AcOEt was added. The organic phase was washed with saturated $NaHCO_3$, 10% citric acid and brine, then dried over Na_2SO_4, filtered and concentrated.

Purification by silica flash chromatography (hexane/EtOAc 8/2) yielded the amide **S15** (3.22 g, 95%) as a white solid.

TLC: R*f*=0.28 (hexane/EtOAc 8/2). **^{1}H NMR** (200 MHz): δ 8.10 (m, 1H), 7.70-7.10(m, 8H), 5.31-5.16 (m, 1H), 3.93-3.53 (m, 2H), 2.50 (s, 3H), 1.88-1.48 (m, 5H), 1.37 (s, 9H).

Synthesis of **2-amino-1-{2-[2-(methylsulfanyl)phenyl]pyrrolidin-1-yl}-2-phenylethan-1-one S16**

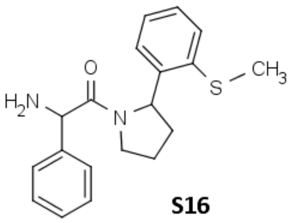

S16

N-boc protected amine **S15** (3.20 g, 7.51 mmol) was dissolved in 5 ml of CH_2Cl_2, then 5 ml of TFA was added and the reaction mixture was let 1 h at room temperature. The reaction mixture was concentrated and 20 ml of EtOAc was added. The organic phase was neutralized with saturated $NaHCO_3$ and washed with brine, then dried over Na_2SO_4, filtered and concentrated to yield the deprotected amine **S16** (2.25 g, 92%) as a white solid.

TLC: R*f*=0.20 (hexane/EtOAc 5/5). **^{1}H-NMR** (200 MHz): δ 7.55-7.05(m, 9H), 5.36-5.20 (m, 1H), 3.83-3.39 (m, 2H), 2.50 (s, 3H), 1.74-1.54 (m, 5H).

Synthesis of **tert-butyl 4-((3-(2-(2-(2-(methylthio)phenyl)pyrrolidin-1-yl)-2-oxo-1-phenylethyl)ureido)methyl)phenylcarbamate S17**

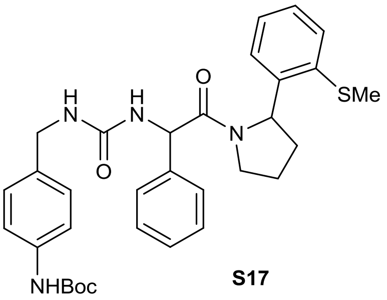

S17

Carbonyl_diimidazole (109 mg, 0.61 mmol) was added to a solution of amine **S16** (200mg, 0.61 mmol, 1.00 equiv) in CH_2Cl_2 (15 mL) and stirred for 2 h at room temperature. 4-(tert-butoxycarbonylamino)-benzylamine (145 mg, 1.10 equiv) was added and the solution was stirred overnight. The reaction mixture was concentrated and 30 ml of EtOAc was added. The organic phase was washed with saturated $NaHCO_3$, 10% citric acid and brine, then dried over Na_2SO_4, filtered and concentrated. Purification by silica flash chromatography (hexane/EtOAc 5/5) yielded the urea **S17** (216 mg, 61%) as a white solid.

TLC: R*f*=0.30 (hexane/EtOAc 5/5). **^{1}H-NMR** (200 MHz): δ 9.26 (s, 1H), 7.45 (m, 4H), 7.32 (m, 3H), 7.18 (m, 6H), 6.22 (d, *J* = 8.5 Hz, 1H), 5.65 (m, 1H), 5.29 (m, 1H), 4.19 (m, 2H), 3.38-3.16(m, 2H), 2.55 (s,3H), 1.78-1.55 (m, 5H), 1.48 (s, 9H).

Synthesis of **1-(4-aminobenzyl)-3-(2-(2-(2-(methylthio)phenyl)pyrrolidin-1-yl)-2-oxo-1-phenylethyl)urea 31**

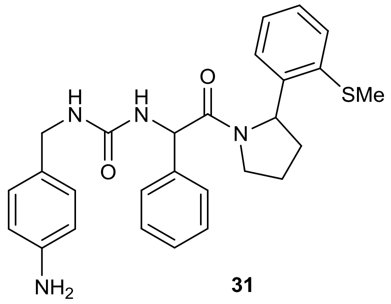

31

N-boc protected amine **S17** (216 mg, 0.38 mmol) was dissolved in 2 ml of CH_2Cl_2, then 2 ml of TFA was added and the reaction mixture was let 1 h at room temperature. The reaction mixture was concentrated and purified by precipitation using diethylether/EtOAc to yield the deprotected amine **30** (TFA salt, 223 mg, 96%) as a yellow solid.

TLC: R*f*=0.44 (100% EtOAc). **^{1}H-NMR** (500 MHz) : δ 7.33-7.18 (m, 8H), 7.09 (d, *J* = 7.2 Hz, 2H), 7.04 (m, 1H), 6.96 (m, 1H), 6.86 (d, *J* = 7.2 Hz, 2H), 6.75 (m, 2H), 6.56 (m, 1H), 6.45 (m, 1H), 6.28 (m, 2H), 5.67 (d, *J* = 7.9 Hz, 1H), 5.58 (d, *J* = 7.9 Hz, 1H), 5.31 (d, *J* = 7.2 Hz, 1H), 5.21 (d, *J* = 8.5 Hz, 1H), 4.91 (m, 4H), 4.02 (m, 6H), 3.83 (m, 2H), 3.50 (m, 3H), 2.20 (m, 2H), 2.08 (m, 2H),

1.90 (m, 5H). **^{13}C-NMR** (500 MHz): δ 169.3, 157.4, 147.9, 141.0, 139.0, 135.5, 135.2, 129.1, 129.0, 128.5, 128.4, 128.3, 128.2, 128.0, 127.9, 127.7, 126.3, 126.2, 125.1, 125.0, 124.7, 124.1, 114.2, 58.7, 58.0, 55.8, 47.4, 43.2, 32.2, 31.9, 23.8, 23.3, 15.7. **ESI-MS** *m/z*: 475.2 $[M + H]^+$.

Scheme 5. Synthesis of compound **33: 1-(5-aminopyridin-2-yl)-3-(2-(2-(2-(methylthio)phenyl)pyrrolidin-1-yl)-2-oxo-1-phenylethyl)urea 33**

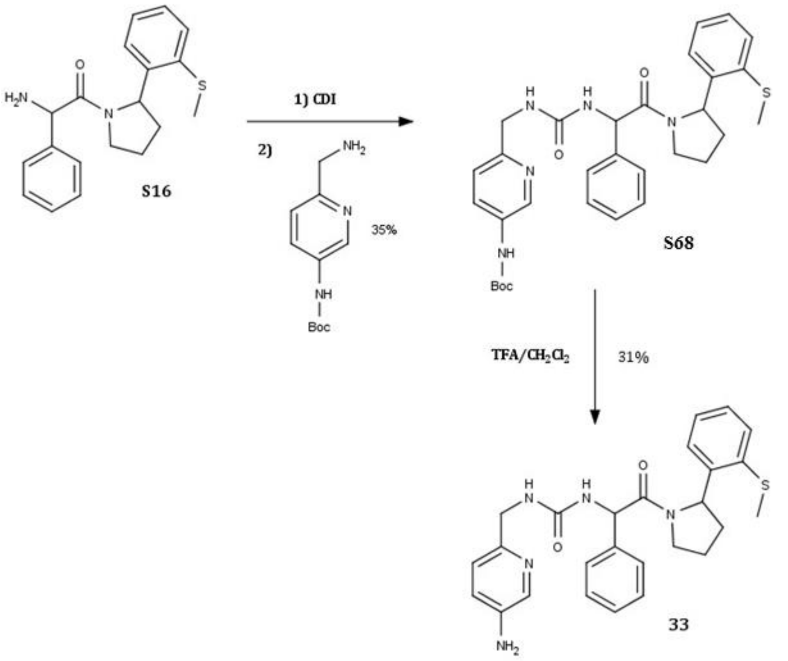

Synthesis of tert-butyl N-[6-({[(2-{2-[2-(methylsulfanyl)phenyl]pyrrolidin-1-yl}-2-oxo-1-phenylethyl)carbamoyl]amino}methyl)pyridin-3-yl]carbamate (**S68).**

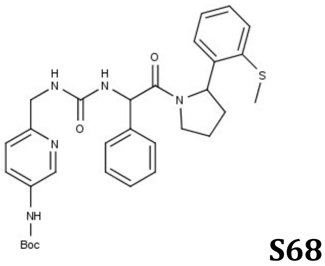

S68

CDI (109 mg, 0.61 mmol, 1 eq) was added to a solution of **S16** (200mg, 0.61 mmol, 1eq) in DCM (15 mL) and stirred for three hours at room temperature. [[5-[(tert-butoxycarbonyl)amino]pyridin-2-yl]methyl]amine (160 mg, 0.67 mmol, 1.1 eq) is added and the solution was stirred overnight. After DCM evaporation, EtOAc (15 mL) was added and the medium was washed with 5% citric acid (2x10mL) and brine (1x10mL). The organic layer was dried over Na_2SO_4 and the solvent was evaporated. The crude product was purified by column chromatography (Hexane/EtOAc 50:50 to EtOAc) to give the desired compound **S68**

with a 35% yield. TLC: Rf=0.12 (Hexane/EtOAc 50:50). ^{1}HNMR (DMSO) δ 9.48 (s,1H), 6.62-7.77 (m,12H), 6.22 (d, *J*=8.5 Hz, 1H), 5.65 (m,1H), 5.29 (m, 1H), 4.19 (m, 2H), 3.16-3.38 (m, 2H), 2.55 (s, 3H), 1.55-1.78 (m, 5H), 1.48 (s, 9H).

Synthesis of 1-(5-aminopyridin-2-yl)-3-(2-(2-(2-(methylthio)phenyl)pyrrolidin-1-yl)-2-oxo-1-phenylethyl)urea **(33)**

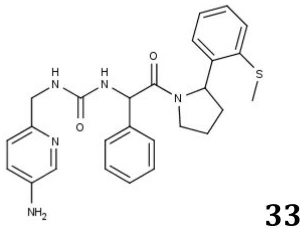

33

S68 was dissolved in a TFA/DCM 1:2 mixture (15 mL) and stirred for 3h at room temperature. DCM was evaporated and EtOAc (20mL) is added. The mixture was neutralized with a saturated solution of $NaHCO_3$. The organic layer was dried over Na_2SO_4 and the solvent was evaporated. The crude product was purified by column chromatography (Hexane/EtOAc 50:50 to EtOAc) and finally precipitated in a mixture of EtOAc and hexane with a 31% yield as a white solid. TLC: Rf=0.48 (EtOAc). ^{1}HNMR (DMSO) δ 7.84 (s, 1H), 6.44-7.47 (m, 12H), 6.23 (d, *J*=7.9 Hz, 1H), 5.66 (d, *J*=7.8 Hz, 1H), 5.26-5.35 (m, 1H), 4.09 (m, 2H), 3.52 (m, 2H), 2.49 (s, 3H), 1.58-1.78 (m, 5H). HPLC purity 95.2%. ESI-MS *m/z*: 476.3 $[M + H]^+$.

Synthesis of ureas S18-S20, S25, S35, S56: General procedure.

Cyano derivative (1 equivalent) was dissolved in 100 ml of MeOH, then a 40 bar pression of hydrogen is applied in the presence of Ni/Raney for 20 h. The reaction mixture is filtered through celite and concentrated. The crude product was purified by flash chromatography to afford the amine. The amine (1 equivalent) was dissolved in DMF (0.4M), then the ethyl isocyanatoacetate (1 equivalent) was added in one portion and the reaction mixture was let 2h at room temperature. After the reaction was complete (TLC control), the reaction mixture was concentrated and purified by flash chromatography to afford the urea.

Synthesis of ethyl 2-(3-(4-amino-3-methoxybenzyl)ureido)acetate **(S18).**

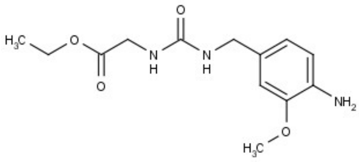

S18

4-amino-3-methylbenzonitrile (0.2 g, 1.12 mmol) was reduced to obtain the 4-(aminomethyl)-2-methoxyaniline (m_{theo}=202 mg). The crude (202 mg, 1.1 mmol) was used to obtain urea **S18** (34 mg, two step global yield=10%, white solid) after purification of the crude product by flash chromatography (EDP/EtOAc). TLC: Rf=0.14 (EDP/EtOAc 30/70). ^{1}H NMR (300 MHz, DMSO): δ 6.74 (s, 1H), 6.62-6.58 (m, 2H), 6.47 (t, *J* = 5.7 Hz, 1H), 6.21 (t, *J* = 6.1 Hz, 1H), 4.62 (broad s, 2H), 4.12 (q, *J* = 7.2 Hz , 2H), 4.09 (d, *J* = 5.7 Hz, 2H), 3.81 (d, *J* = 6.1 Hz, 2H), 3.77 (s, 3H), 1.22 (t, *J* = 7.1 Hz, 3H). HPLC purity 99.1%. ESI-MS *m/z*: 282.2 [M + H]$^+$.

Synthesis of ethyl 2-(3-(4-amino-3-methylbenzyl)ureido)acetate **(S19).**

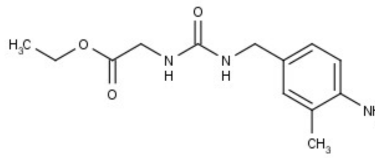

S19

4-amino-3-methylbenzonitrile (0.2 g, 1.5 mmol) was reduced to obtain the 4-(aminomethyl)-2-methylaniline (m_{theo}=206 mg) as a yellow oil. The crude (206 mg, 1.5 mmol) was used to obtain urea **S19** (108 mg, two step global yield=27%, white solid) after purification of the crude product by flash chromatography (EDP/EtOAc). TLC: Rf=0.18 (EDP/EtOAc 30/70). ^{1}H NMR (200 MHz, DMSO): δ 6.88-6.96 (m, 2H), 6.56 (d, *J* = 7.9 Hz, 1H), 6.47-6.37 (m, 1H), 6.18 (t, *J* = 5.9 Hz, 1H), 4.75 (broad s, 2H), 4.12 (q, *J* = 7.0 Hz, 2H), 4.03 (d, *J* = 6.2 Hz, 2H), 3.80 (d, *J* = 6.0 Hz, 2H), 2.06 (s, 3H), 1.23 (t, *J* = 7.1 Hz, 3H). HPLC purity 99.2%. ESI-MS *m/z*: 266.2 $[M + H]^+$.

Synthesis of ethyl 2-(3-(4-amino-3-ethylbenzyl)ureido)acetate **(S20).**

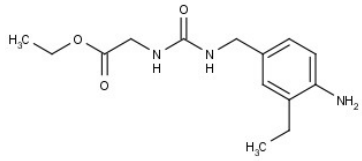

S20

4-amino-3-ethylbenzonitrile (0.2 g, 1.37 mmol) was reduced to obtain the 4-(aminomethyl)-2-ethylaniline (m_{theo}=205 mg) as a yellow oil. The crude (205 mg, 1.37 mmol) was used to obtain urea **S20** (92 mg, two step global yield=24%, white solid) after purification of the crude product by flash chromatography (EDP/EtOAc). TLC: Rf=0.3 (EDP/EtOAc 30/70). ^{1}H NMR (200 MHz, CDCl3): δ 7.02-6.88 (m, 2H), 6.61 (d, *J* = 7.8 Hz, 1H), 5.02 (t, *J* = 5.2 Hz, 1H), 4.89 (t, *J* = 5.4 Hz, 1H), 4.23 (d, *J* = 5.5 Hz, 1H), 4.15 (q, *J* = 7.2 Hz, 2H), 3.97 (d, *J* = 5.3 Hz, 2H), 3.62 (broad s, 2H), 2.48 (q, *J* = 7.5 Hz, 2H), 1.25 (t, *J* = 7,1 Hz, 3H), 1.22 (t, *J* = 7.4 Hz, 3H). HPLC purity 97.2%. ESI-MS *m/z*: 280.2 $[M + H]^+$.

Synthesis of ethyl 2-(3-((4-aminonapht-1-yl)methyl)ureido)acetate **(S25).**

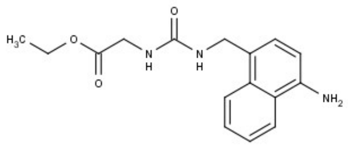

S25

4-Amino-1-naphthalenecarbonitrile (0.5 g, 2.98 mmol) was reduced to obtain the 4-aminomethyl-1-amino-naphthalene (0.22g, 43%) after purification by flash chromatography (AcOEt/MeOH 7/3). TLC: Rf=0.09 (AcOEt/MeOH 7/3). ^{1}H NMR (DMSO): δ 1.99 (s, 2H), 4.05 (s, 2H), 5.62 (s, 2H), 6.60 (d, 1H, *J* = 9.6 Hz), 7.20 (d, 1H, *J* = 7.6 Hz), 7.35 (m, 2H), 8.08 (m, 2H). The 4-aminomethyl-1-aminonaphthalene (154 mg, 0.89 mmol) was used to obtain urea **S25** (12 mg, 5%, yellow solid) after purification of the crude product by flash chromatography (AcOEt). TLC: Rf=0.52 (AcOEt). ^{1}H NMR (DMSO): δ 1.19 (t, 3H, *J*= 7.1 Hz), 3.79 (d, 2H, *J* = 6.0 Hz), 4.08 (q, 2H, *J*= 7.1 Hz), 4.49 (d, 2H, *J* = 5.2 Hz), 5.66 (s, 2H), 6.12 (t, 1H, *J* = 6.0 Hz), 6.42 (t, 1H, *J* = 5.2 Hz), 6.61 (d, 1H, *J*= 7.6 Hz), 7.15 (d, 1H, *J*= 7.6 Hz), 7.45 (m, 2H), 7.91 (d, 1H, *J* = 8.1 Hz), 8.09 (d, 1H, *J* = 8.1 Hz). HPLC purity 99,7%. ESI-MS *m/z*: 302.3 $[M + H]^+$.

Synthesis of ethyl 2-(3-(4-hydroxybenzyl)ureido)acetate **(S35).**

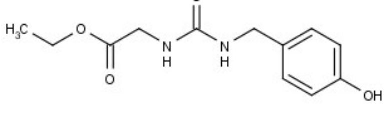

S35

4-cyano-phenol (0.3 g, 2.52 mmol) was reduced to obtain the 4-aminomethylphenol (0.13g, 43%) after purification by flash chromatography (AcOEt/MeOH 7/3). TLC: Rf=0.09 (AcOEt/MeOH 7/3). ^{1}H NMR (DMSO): δ 2.40 (s, 2H), 3.53 (s, 2H), 6.69 (d, 2H, *J* = 8.4 Hz), 7.11

(d, 2H, *J* = 8.4 Hz), 9.20 (s, 1H). The 4-aminomethylphenol (48 mg, 0.39 mmol) was used to obtain urea **S35** (23 mg, 26%, yellow solid) after purification of the crude product by flash chromatography (DCM/MeOH 95/5). TLC: Rf=0.34 (DCM/MeOH 95/5). ^{1}H NMR (DMSO): δ 1.19 (t, 3H, *J*= 7.1 Hz), 3.77 (d, 2H, *J* = 6.0 Hz), 4.08 (m, 4H), 6.20 (t, 1H, *J* = 6.0 Hz), 6.51 (t, 1H, *J* = 5.6 Hz), 6.69 (d, 2H, *J*= 8.4 Hz), 7.05 (d, 2H, *J*= 8.4 Hz), 9.25 (s, 1H). HPLC purity 96.6%. ESI-MS *m/z*: 253.2 [M + H]$^+$.

Synthesis of ethyl 2-(3-((indol-5-yl)methyl)ureido)acetate **(S56).**

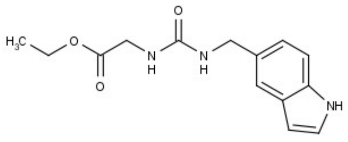

S56

5-cyano-indole (0.3 g, 2.11 mmol) was reduced to obtain the 5-aminomethylindole (0.18g, 59%) after purification by flash chromatography (AcOEt/MeOH 7/3 then MeOH). TLC: Rf=0.09 (AcOEt/MeOH 7/3). ^{1}H NMR (DMSO): δ 2.40 (s, 2H), 3.78 (s, 2H), 6.38 (m, 1H), 7.10 (d, 1H, *J* = 8.3 Hz), 7.29 (m, 1H), 7.33 (d, 1H, *J* = 8.3 Hz), 7.49 (s, 1H), 11.00 (s, 1H). The 5-aminomethylindole (57 mg, 0.39 mmol) was used to obtain urea **S56** (63 mg, 66%) after treatment of the crude product by EDP. TLC: Rf=0.57 (AcOEt). ^{1}H NMR (DMSO): δ 1.21 (t, 3H, *J*= 7.1 Hz), 3.81 (d, 2H, *J* = 6.0 Hz), 4.11 (q, 2H, *J* = 7.1 Hz), 4.28 (d, 2H, *J* = 5.7 Hz), 6.24 (t, 1H, *J* = 6.0 Hz), 6.39 (s, 1H), 6.58 (t, 1H, *J* = 5.7 Hz), 7.01 (d, 1H, *J*= 8.3 Hz), 7.38 (m, 3H), 11.03 (s, 1H). HPLC purity 97.3%. ESI-MS *m/z*: 276.2 [M + H]$^+$.

Synthesis of ureas S21-S24, S26-S34, S36-39, S41-S55, S57-S68, General procedure, and synthesis of compound 32:

Ethyl isocyanatoacetate (1 equivalent, 100 mg, 87 μl, 0.77 mmol) was dissolved in THF (0.4 M) or in DMF (0.4M). One equivalent of triethylamine was added if the amine is a salt form of HCl, then the amine (1 equivalent) was added in one portion and the reaction mixture was let 2h at room temperature. After the reaction was complete (TLC control), the reaction mixture was concentrated and purified with different procedures.

Synthesis of ethyl 2-(3-(3-aminobenzyl)ureido)acetate **(S21).**

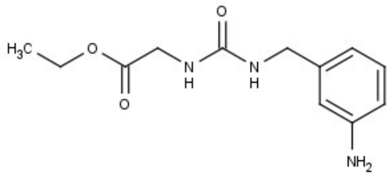

S21

The crude product was purified by HPLC preparative to afford the urea **S21** (61mg; 31%) as a white solid. ^{1}H NMR ($CDCl_3$): δ 1.21 (t, 3H, *J*= 7.1 Hz), 3.80 (d, 2H, *J*= 6.0 Hz), 4.10 (m, 4H), 5.03 (s, 2H), 6.25 (t, 1H, *J* = 6.0 Hz), 6.45 (m, 3H), 6.53 (t, 1H, *J*= 6.0 Hz), 6.95 (t, 1H, *J*= 7.5 Hz). HPLC purity 99.9%. ESI-MS *m/z*: 252.2 [M + H]$^+$.

Synthesis of ethyl 2-(3-(2-fluoro-6-aminobenzyl)ureido)acetate **(S22).**

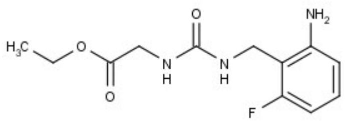

S22

The crude product was purified by HPLC preparative to afford the urea **S22** (182 mg; 43%) as a white solid Rf=0.76 (AcOEt). ^{1}H NMR (DMSO): δ 1.19 (t, 3H, *J*= 7.1 Hz), 3.78 (d, 2H, *J* = 6.0 Hz), 4.11 (m, 4H), 5.58 (s, 2H), 6.19 (t, 1H, *J* = 6.0 Hz), 6.29 (t, 1H, *J* = 8.1 Hz), 6.43 (d, 1H, *J* = 8.1 Hz), 6.69 (t, 1H, *J* = 6.0 Hz), 6.95 (m, 1H). HPLC purity 99.5%. ESI-MS *m/z*: 270.2 [M + H]$^{+}$.

Synthesis of ethyl 2-(3-benzyl)ureido)acetate **(S23).**

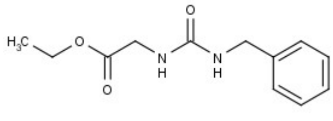

S23

S23 was purified by precipitation in EDP to afford 222 mg of a white solid (99%). ^{1}H NMR (DMSO): δ 1.20 (t, 3H, *J*= 7.5 Hz), 3.79 (d, 2H, *J* = 5.9 Hz), 4.09 (q, 2H, *J*= 7.5 Hz), 4.22 (d, 2H, *J* = 6.2 Hz), 6.29 (t, 1H, *J* = 6.2 Hz), 6.67 (t, 1H, *J* = 5.9 Hz), 7.25 (m, 3H), 7.31 (m, 2H). HPLC purity 99.4%. ESI-MS *m/z*: 237.2 [M + H]$^{+}$.

Synthesis of ethyl 2-(3-((napht-1-yl)methyl)ureido)acetate **(S24).**

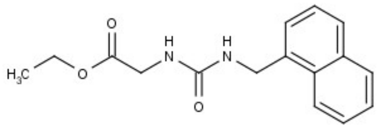

S24

S24 was purified by precipitation in EDP to afford 197 mg of a white solid (92%). ^{1}H NMR (DMSO): δ 1.20 (t, 3H, *J*= 7.0 Hz), 3.81 (d, 2H, *J* = 5.6 Hz), 4.10 (q, 2H, *J*= 7.0 Hz), 4.69 (d, 2H, *J* = 5.6 Hz), 6.29 (t, 1H, *J* = 5.6 Hz), 6.72 (t, 1H, *J* = 5.6 Hz), 7.46 (m, 2H), 7.55 (m, 2H), 7.84 (d, 1H, *J* = 7.2 Hz), 7.95 (d, 1H, *J*= 7.7 Hz), 8.09 (d, 1H, *J*= 7.7 Hz). HPLC purity 98.2%. ESI-MS *m/z*: 287.2 [M + H]$^{+}$.

Synthesis of ethyl 2-(3-(3-methoxybenzyl)ureido)acetate **(S26).**

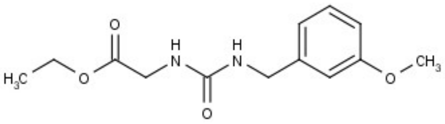

S26

S26 was purified by precipitation in EDP to afford 199 mg of a white solid (97%). TLC: Rf=0.71 (AcOEt). ^{1}H NMR (DMSO): δ 1.19 (t, 3H, *J*= 7.1 Hz), 3.73 (s, 3H), 3.78 (d, 2H, *J* = 6.0 Hz), 4.08 (q, 2H, *J* = 7.1 Hz), 4.18 (d, 2H, *J* = 6.0 Hz), 6.29 (t, 1H, *J* = 6.0 Hz), 6.66 (t, 1H, *J* = 6.0 Hz), 6.81 (m, 3H), 7.22 (t, 1H, *J*= 8.0 Hz). HPLC purity 100%. ESI-MS *m/z*: 267.2 $[M + H]^+$.

Synthesis of ethyl 2-(3-(4-methoxybenzyl)ureido)acetate **(S27).**

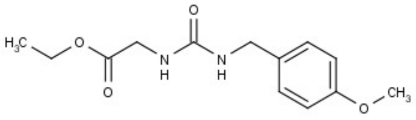

S27

S27 was purified by precipitation in EDP to afford 182 mg of a white solid (83%). ^{1}H NMR (DMSO): δ 1.20 (t, 3H, *J*= 7.8 Hz), 3.73 (s, 3H), 3.78 (d, 2H, *J* = 6.1 Hz), 4.09 (q, 2H, *J*= 7.8 Hz), 4.13 (d, 2H, *J* = 6.0 Hz), 6.24 (t, 1H, *J* = 6.0 Hz), 6.58 (t, 1H, *J* = 6.1 Hz), 6.87 (d, 2H, *J* = 8.3 Hz), 7.17 (d, H, *J*= 8.3 Hz). HPLC purity 98.6%. ESI-MS *m/z*: 267.2 $[M + H]^+$.

Synthesis of ethyl 2-(3-(4-chlorobenzyl)ureido)acetate **(S28).**

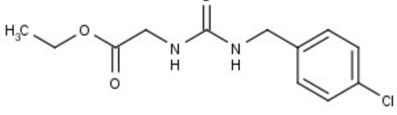

S28

S28 was purified by precipitation in EDP to afford 205 mg of a white solid (98%). TLC: Rf=0.48 (AcOEt). ^{1}H NMR (DMSO): δ 1.19 (t, 3H, *J*= 7.4 Hz), 3.78 (d, 2H, *J* = 6.1 Hz), 4.09 (q, 2H,

J= 7.4 Hz), 4.20 (d, 2H, J = 5.8 Hz), 6.34 (t, 1H, J = 6.1 Hz), 6.72 (t, 1H, J = 5.8 Hz), 7.27 (d, 2H, J = 7.8 Hz), 7.38 (d, 2H, J= 7.8 Hz). HPLC purity 99.2%. ESI-MS *m/z*: 271.3/273.1 $[M + H]^+$.

Synthesis of ethyl 2-(3-(3-chlorobenzyl)ureido)acetate **(S29).**

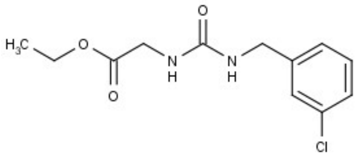

S29

S29 was purified by precipitation in EDP to afford 209 mg of a white solid (99%). TLC: Rf=0.48 (AcOEt). ^{1}H NMR (DMSO): δ 1.19 (t, 3H, J= 7.1 Hz), 3.78 (d, 2H, J = 6.0 Hz), 4.09 (q, 2H, J = 7.1 Hz), 4.22 (d, 2H, J = 6.0 Hz), 6.36 (t, 1H, J = 6.0 Hz), 6.75 (t, 1H, J = 6.0 Hz), 7.05 (d, 1H, J= 7.3 Hz), 7.27 (m, 3H). HPLC purity 99.0%. ESI-MS *m/z*: 271.3/273.1 $[M + H]^+$.

Synthesis of ethyl 2-(3-(3,5-dichlorobenzyl)ureido)acetate **(S30).**

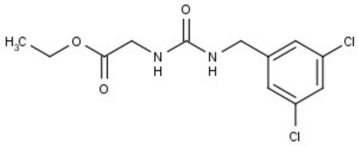

S30

S30 was purified by precipitation in EDP to afford 217 mg of a white solid (92%). TLC: Rf=0.75 (AcOEt). ^{1}H NMR (DMSO): δ 1.19 (t, 3H, J= 7.1 Hz), 3.32 (d, 2H, J = 6.0 Hz), 4.09 (q, 2H, J = 7.1 Hz), 4.22 (d, 2H, J = 6.1 Hz), 6.44 (t, 1H, J = 6.1 Hz), 6.81 (t, 1H, J = 6.0 Hz), 7.29 (d, 2H, J= 1.9 Hz), 7.46 (t, 1H, J= 1.9 Hz). HPLC purity 96.1%. ESI-MS *m/z*: 305.1/307.1 $[M + H]^+$.

Synthesis of ethyl 2-(3-(3-fluorobenzyl)ureido)acetate **(S31).**

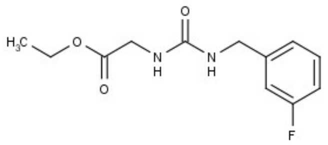

S31

S31 was purified by precipitation in EDP to afford 194 mg of a white solid (98%). TLC Rf=0.55 (AcOEt). ^{1}H NMR (DMSO): δ 1.19 (t, 3H, *J*= 7.1 Hz), 3.78 (d, 2H, *J* = 6.0 Hz), 4.08 (q, 2H, *J* = 7.1 Hz), 4.23 (d, 2H, *J* = 6.0 Hz), 6.36 (t, 1H, *J* = 6.0 Hz), 6.75 (t, 1H, *J* = 6.0 Hz), 7.05 (m, 3H), 7.35 (m, 1H). HPLC purity 97.5%. ESI-MS *m/z*: 255.2 $[M + H]^+$.

Synthesis of ethyl 2-(3-(4-fluorobenzyl)ureido)acetate **(S32).**

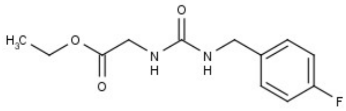

S32

S32 was purified by precipitation in AcOEt/EDP to afford 177 mg of a white solid (90%). TLC: Rf=0.55 (AcOEt). ^{1}H NMR (DMSO): δ 1.19 (t, 3H, *J*= 7.1 Hz), 3.78 (d, 2H, *J* = 6.0 Hz), 4.08 (q, 2H, *J* = 7.1 Hz), 4.19 (d, 2H, *J* = 6.0 Hz), 6.30 (t, 1H, *J* = 6.0 Hz), 6.69 (t, 1H, *J* = 6.0 Hz), 7.14 (m, 2H), 7.29 (m, 2H). HPLC purity 98.1%. ESI-MS *m/z*: 255.2 $[M + H]^+$.

Synthesis of ethyl 2-(3-(3-trifluoromethylbenzyl)ureido)acetate **(S33).**

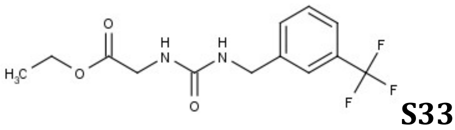

S33

S33 was purified by precipitation in EDP to afford 213 mg of a white solid (90%). TLC: Rf=0.53 (AcOEt). ^{1}H NMR (DMSO): δ 0.95 (t, 3H, *J*= 7.1 Hz), 3.54 (d, 2H, *J* = 6.1 Hz), 3.85 (q, 2H,

J = 7.1 Hz), 4.06 (d, 2H, J = 6.1 Hz), 6.14 (t, 1H, J = 6.1 Hz), 6.58 (t, 1H, J = 6.1 Hz), 7.64 (m, 4H). HPLC purity 95.2%. ESI-MS m/z: 305.2 $[M + H]^+$.

Synthesis of ethyl 2-(3-(4-bromobenzyl)ureido)acetate **(S34).**

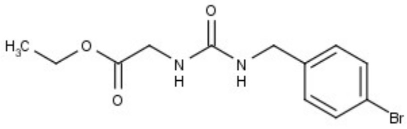

S34

S34 was purified by precipitation in EDP to afford 100 mg of a white solid (41%). TLC: Rf=0.70 (AcOEt). ^{1}H NMR (DMSO): δ 1.19 (t, 3H, J= 7.1 Hz), 3.78 (d, 2H, J = 6.0 Hz), 4.08 (q, 2H, J = 7.1 Hz), 4.18 (d, 2H, J = 6.0 Hz), 6.30 (t, 1H, J = 6.0 Hz), 6.72 (t, 1H, J = 6.0 Hz), 7.20 (d, 2H, J= 8.1 Hz), 7.50 (d, 2H, J= 8.1 Hz). HPLC purity 98.4%. ESI-MS m/z: 315.1/317.1 $[M + H]^+$.

Synthesis of ethyl 2-(3-(3,4-dihydroxybenzyl)ureido)acetate **(S36).**

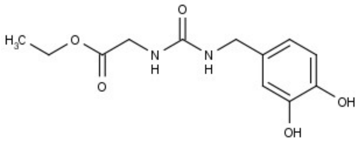

S36

The crude product was purified by HPLC preparative to afford the urea **S36** (81 mg; 36%) as a white solid. ^{1}H NMR (DMSO): δ 1.19 (t, 3H, J= 7.1 Hz), 3.78 (d, 2H, J = 6.0 Hz), 4.02 (q, 2H, J= 7.1 Hz), 4.09 (d, 2H, J= 7.1 Hz), 6.18 (t, 1H, J = 6.0 Hz), 6.49 (m, 2H), 6.64 (m, 2H), 8.71 (s, 1H), 8.81 (s, 1H). HPLC purity 100%. ESI-MS m/z: 269.2 $[M + H]^+$.

Synthesis of ethyl 2-(3-(2,4-dihydroxybenzyl)ureido)acetate **(S37).**

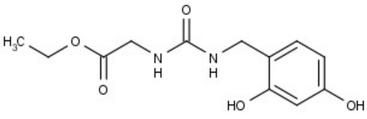

S37

The residue was taking up with AcOEt, the organic phase was washed with a solution of 10% citric acid and brine, dried over Na_2SO_4, filtrated and concentrated. The crude product was purified by flash chromatography (EtOAc) to afford the urea 30 (28 mg; 19%) as a white solid. TLC: Rf=0.44 (AcOEt). ^{1}H NMR (DMSO): δ 1.20 (t, 3H, *J*= 6.9 Hz), 3.78 (d, 2H, *J*= 5.6 Hz), 4.02 (d, 2H, *J*= 5.6 Hz), 4.09 (q, 2H, *J*= 6.9 Hz), 6.18 (t, 1H, *J* = 5.6 Hz), 6.46 (t, 1H, *J*= 5.6 Hz), 6.50 (d, 1H, *J*= 8.2 Hz), 6.65 (m, 2H), 8.69 (s, 1H), 8.78 (s, 1H). HPLC purity 98.7%. ESI-MS *m/z*: 269.2 $[M + H]^+$.

Synthesis of ethyl 2-(3-(3-methoxy-4-hydroxybenzyl)ureido)acetate **(S38).**

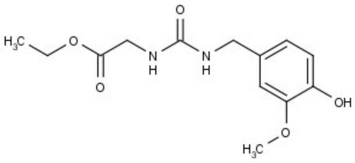

S38

The crude product was purified by flash chromatography (EtOAc/EDP 8/2) to afford the urea **S38** (30 mg; 17%) as a white solid. TLC: Rf=0.14 (AcOEt/EDP 8/2). ^{1}H NMR (DMSO): δ 1.21 (t, 3H, *J*= 7.1 Hz), 3.76 (s, 3H), 3.80 (d, 2H, *J* = 6.0 Hz), 4.10 (m, 4H), 6.24 (t, 1H, *J* = 6.0 Hz), 6.56 (t, 1H, *J* = 6.0 Hz), 6.70 (m, 2H), 6.84 (s, 1H), 8.83 (sl, 1H). HPLC purity 98.0%. ESI-MS *m/z*: 283.2 $[M + H]^+$.

Synthesis of ethyl 3-((3-(2-ethoxy-2-oxoethyl)ureido)methyl)benzoate **(S39).**

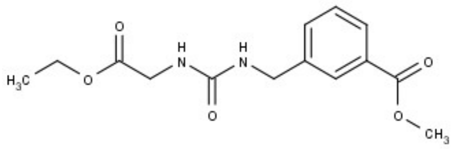

S39

The crude product was purified by flash chromatography (EtOAc) to afford the urea **S39**(41 mg; 29%) as a white solid. TLC: Rf=0.55 (AcOEt). ^{1}H NMR (DMSO): δ 1.20 (t, 3H, *J*= 7.1 Hz), 3.80 (d, 2H, *J* = 6.1 Hz), 3.87 (s, 3H), 4.10 (q, 2H, *J* = 7.1 Hz), 4.29 (d, 2H, *J* = 6.1 Hz), 6.38 (t, 1H, *J* = 6.1 Hz), 6.81 (t, 1H, *J* = 6.1 Hz), 7.52 (m, 2H), 7.86 (m, 2H). HPLC purity 100%. ESI-MS *m/z*: 295.2 $[M + H]^{+}$.

Synthesis of ethyl 2-(3-(pyridin-4-ylmethyl)ureido)acetate **(S41).**

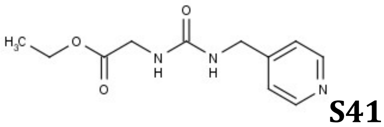

S41

S41 was purified by precipitation in EDP to afford 202 mg of a white solid (93%). ^{1}H NMR (DMSO): δ 1.20 (t, 3H, *J*= 7.0 Hz), 3.79 (d, 2H, *J* = 5.3 Hz), 4.10 (q, 2H, *J*= 7.0 Hz), 4.25 (d, 2H, *J* = 4.9 Hz), 6.45 (t, 1H, *J* = 5.3 Hz), 6.81 (t, 1H, *J* = 4.9 Hz), 7.24 (d, 2H, *J* = 4.3 Hz), 8.49 (d, 2H, *J*= 4.3 Hz). HPLC purity 97.6%. ESI-MS *m/z*: 238.2 $[M + H]^{+}$.

Synthesis of ethyl 2-(3-(pyridin-3-ylmethyl)ureido)acetate **(S42).**

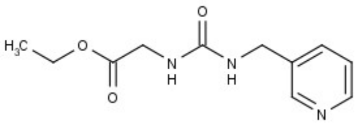

S42

S42 was purified by precipitation in EDP to afford 208 mg of a white solid (94%). ^{1}H NMR (DMSO): δ 1.20 (t, 3H, *J*= 7.1 Hz), 3.78 (d, 2H, *J* = 6.4 Hz), 4.09 (q, 2H, *J*= 7.1 Hz), 4.24 (d, 2H, *J* = 6.4 Hz), 6.37 (t, 1H, *J* = 6.4 Hz), 6.77 (t, 1H, *J* = 6.4 Hz), 7.34 (m, 1H), 7.65 (d, 1H, *J* = 7.7 Hz), 8.44 (d, 1H, *J*= 4.6 Hz), 8.48 (s, 1H). HPLC purity 99.2%. ESI-MS *m/z*: 238.2 $[M + H]^+$.

Synthesis of ethyl 2-(3-(pyridin-2-ylmethyl)ureido)acetate **(S43).**

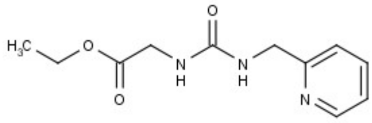

S43

S43 was purified by precipitation in EDP to afford 201 mg of a white solid (92%). ^{1}H NMR (DMSO): δ 1.20 (t, 3H, *J*= 7.1 Hz), 3.79 (d, 2H, *J* = 6.3 Hz), 4.09 (q, 2H, *J*= 7.1 Hz), 4.31 (d, 2H, *J* = 5.9 Hz), 6.48 (t, 1H, *J* = 6.3 Hz), 6.80 (t, 1H, *J* = 5.9 Hz), 7.25 (m, 1H), 7.29 (d, 1H, *J* = 7.9 Hz), 7.76 (t, 1H, *J*= 7.9 Hz), 8.50 (d, 1H, *J*= 4.3 Hz). HPLC purity 98.6%. ESI-MS *m/z*: 238.2 $[M + H]^+$.

Synthesis of ethyl 2-(3-((6-aminopyridin-3-yl)methyl)ureido)acetate **(S44).**

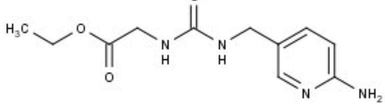

S44

The crude product was purified by flash chromatography (EtOAc/MeOH 7/3) to afford the urea **S44** (90 mg; 71%) as a white solid. TLC: Rf=0.28 (AcOEt/MeOH 7/3). ^{1}H NMR (DMSO): δ 0.97 (t, 3H, *J*= 7.1 Hz), 3.54 (d, 2H, *J* = 6.0 Hz), 3.77 (d, 2H, *J*= 5.7 Hz), 3.84 (q, 2H, *J* = 7.1 Hz), 5.55 (s, 2H), 5.97 (t, 1H, *J* = 6.0 Hz), 6.16 (d, 1H, *J* = 8.4 Hz), 6.25 (t, 1H, *J* = 5.7 Hz), 7.03 (d, 1H, *J*= 8.4 Hz), 7.56 (s, 1H). HPLC purity 100%. ESI-MS *m/z*: 253.2 $[M + H]^+$.

Synthesis of ethyl 2-(3-(2-morpholinobenzyl)ureido)acetate **(S45).**

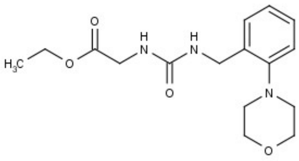

S45

S45 was purified by precipitation in AcOEt/EDP to afford 200 mg of a white solid (87%). TLC: Rf=0.52 (AcOEt). ^{1}H NMR (DMSO): δ 1.19 (t, 3H, *J*= 7.1 Hz), 2.82 (t, 4H, *J* = 4.3 Hz), 3.74 (t, 4H, *J* = 4.3 Hz), 3.79 (d, 2H, *J* = 6.0 Hz), 4.08 (q, 2H, *J* = 7.1 Hz), 4.28 (d, 2H, *J* = 5.8 Hz), 6.32 (t, 1H, *J* = 6.0 Hz), 6.56 (t, 1H, *J* = 5.8 Hz), 7.05 (d, 1H, *J* = 7.4 Hz), 7.11 (d, 1H, *J* = 7.3 Hz), 7.22 (d, 1H, *J* = 7.4 Hz), 7.27 (d, 1H, *J* = 7.3 Hz). HPLC purity 100%. ESI-MS *m/z*: 322.2 [M + H]$^{+}$.

Synthesis of ethyl 2-(3-(benzo[*d*][1,3]dioxol-5-ylmethyl)ureido)acetate **(S46).**

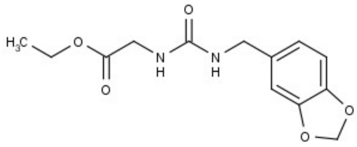

S46

S46 was purified by precipitation in EDP to afford 142 mg of a white solid (65%). TLC: Rf=0.32 (AcOEt). ^{1}H NMR (DMSO): δ 1.19 (t, 3H, *J*= 7.1 Hz), 3.77 (d, 2H, *J*= 6.0 Hz), 4.09 (m, 4H), 5.97 (s, 1H), 6.25 (t, 1H, *J* = 6.0 Hz), 6.62 (t, 1H, *J*= 6.0 Hz), 6.75 (d, 1H, *J*= 8.3; 1.9 Hz), 7.80 (m, 2H). HPLC purity 99.6%. ESI-MS *m/z*: 281.2 [M + H]$^{+}$.

Synthesis of ethyl 2-(3-(3-pyrimidin-2-yl)benzyl)ureido)acetate **(S47).**

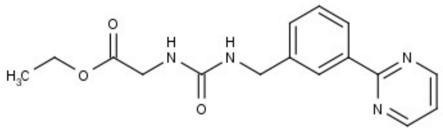

S47

S47 was purified by precipitation in EDP to afford 158 mg of a white solid (65%). TLC: Rf=0.22 (AcOEt). ^{1}H NMR (DMSO): δ 1.25 (t, 3H, *J*= 7.1 Hz), 3.85 (d, 2H, *J* = 6.0 Hz), 4.15 (q, 2H, *J* = 7.1 Hz), 4.37 (d, 2H, *J* = 6.0 Hz), 6.40 (t, 1H, *J* = 5.8 Hz), 6.85 (t, 1H, *J* = 5.8 Hz), 7.50 (m, 3H), 8.32 (dd, 1H, J= 7.4, 1.2 Hz), 8.38 (s, 1H), 8.95 (dd, 2H, *J* = 4.8, 1.2 Hz). HPLC purity 95.1%. ESI-MS *m/z*: 315.2 [M + H]+.

Synthesis of ethyl 2-(3-((2,3-dihydrobenzo[*b*][1,4]dioxin-5-yl)methyl)ureido)acetate **(S48).**

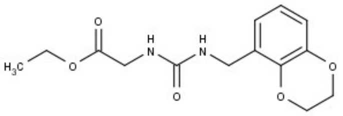

S48

The residue was taking up with AcOEt, the organic phase was washed with a solution of 10% citric acid and brine, dried over Na_2SO_4, filtrated and concentrated to afford **S48** as a white solid (148 mg; 66%). TLC: Rf=0.47 (AcOEt). ^{1}H NMR (DMSO): δ 1.24 (t, 3H, *J*= 7.1 Hz), 3.84 (d, 2H, *J*= 6.0 Hz), 4.16 (q, 2H, *J*= 7.1 Hz), 4.21 (d, 2H, *J*= 6.0 Hz), 4.33 (m, 4H), 6.37 (t, 1H, *J* = 6.0 Hz), 6.55 (t, 1H, *J*= 6.0 Hz), 6.81 (m, 3H). HPLC purity 99.6%. ESI-MS *m/z*: 295.2 [M + H]+.

Synthesis of ethyl 2-(3-(3-(morpholinomethyl)benzyl)ureido)acetate **(S49).**

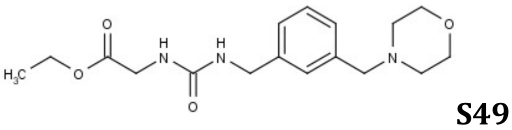

S49

The crude product was purified by flash chromatography (EtOAc/MeOH 98/2) to afford the urea **S49** (210 mg; 81%) as a white solid. TLC: Rf=0.31 (AcOEt). ^{1}H NMR ($CDCl_3$): δ 1.17 (t, 3H, *J*= 7.1 Hz), 2.34 (d, 4H, *J* = 4.2 Hz), 3.37 (s, 2H), 3.61 (m, 4Hz), 3.87 (m, 2H), 4.06 (q, 2H, *J* = 7.1 Hz), 4.24 (d, 2H, *J* = 5.7 Hz), 5.40 (sl, 2H), 7.13 (m, 4H). HPLC purity 96.5%. ESI-MS *m/z*: 336.2 $[M + H]^+$.

Synthesis of ethyl 2-(3-((2-furfur2-yl)benzyl)ureido)acetate **(S50).**

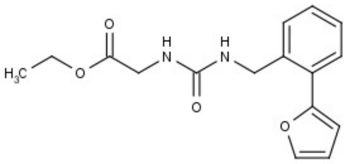

S50

S50 was purified by precipitation in EDP to afford 230 mg of a white solid (98%). TLC: Rf=0.71 (AcOEt). ^{1}H NMR (DMSO): δ 1.19 (t, 3H, *J*= 7.1 Hz), 3.79 (d, 2H, *J* = 6.0 Hz), 4.08 (q, 2H, *J* = 7.1 Hz), 4.38 (d, 2H, *J* = 5.8 Hz), 6.39 (t, 1H, *J* = 5.8 Hz), 6.63 (m, 1H), 6.68 (t, 1H, *J* = 5.8 Hz), 6.75 (d, 1H, *J*= 3.3 Hz), 7.33 (m, 3H), 7.65 (m, 1H), 7.81 (s, 1H). HPLC purity 95.7%. ESI-MS *m/z*: 303.2 $[M + H]^+$.

Synthesis of ethyl 2-(3-((2-morpholino-pyridin-4-yl)methyl)ureido)acetate **(S51).**

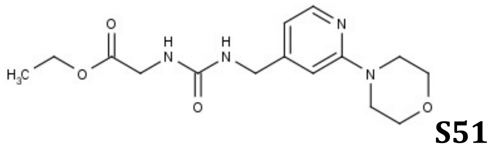

S51

S51 was purified by precipitation in EDP to afford 133 mg of a white solid (53%). TLC: Rf=0.19 (AcOEt). ^{1}H NMR (DMSO): δ 1.19 (t, 3H, *J*= 7.1 Hz), 3.42 (t, 4H, *J*= 4.9 Hz), 3.69 (t, 4H,

J= 4.9 Hz,), 3.78 (d, 2H, *J*= 6.1 Hz), 4.08 (q, 2H, *J*= 7.1 Hz), 4.15 (d, 2H, *J*= 6.0 Hz), 6.33 (t, 1H, *J* = 6.1 Hz), 6.57 (d, 1H, *J*= 5.0 Hz), 6.68 (s, 1H), 6.72 (t, 1H, *J*= 6.1 Hz), 8.03 (d, 1H, *J*= 5.0 Hz). HPLC purity 100%. ESI-MS *m/z*: 323.2 $[M + H]^+$.

Synthesis of ethyl 2-(3-(3-(*1H*-1,2,4-triazol-1-yl)benzyl)ureido)acetate **(S52).**

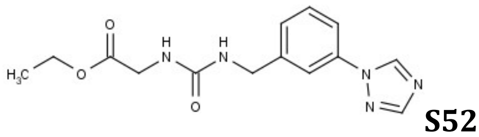

S52

S52 was purified by precipitation in EDP to afford 208 mg of a white solid (89%). TLC: Rf=0.11 (AcOEt). ^{1}H NMR (DMSO): δ 1.18 (t, 3H, *J*= 7.1 Hz), 3.79 (d, 2H, *J*= 6.0 Hz), 4.08 (q, 2H, *J*= 7.1 Hz), 4.31 (d, 2H, *J*= 6.0 Hz), 6.38 (t, 1H, *J* = 6.0 Hz), 6.81 (t, 1H, *J*= 6.0 Hz), 7.30 (d, 1H, *J*= 7.6 Hz), 7.50 (t, 1H, *J*= 7.7 Hz), 7.73 (m, 2H), 8.24 (s, 1H), 9.27 (s, 1H). HPLC purity 98.7%. ESI-MS *m/z*: 304.2 $[M + H]^+$.

Synthesis of ethyl 2-(3-(3-(*1H*-pyrazol-1-yl)benzyl)ureido)acetate **(S53).**

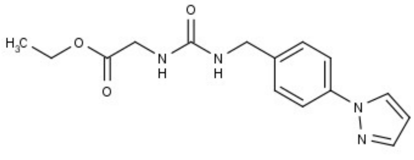

S53

S53 was purified by precipitation in EDP to afford 224 mg of a white solid (95%). TLC: Rf=0.38 (AcOEt). ^{1}H NMR (DMSO): δ 1.21 (t, 3H, *J*= 7.1 Hz), 3.77 (d, 2H, *J*= 6.0 Hz), 4.08 (q, 2H, *J*= 7.1 Hz), 4.14 (d, 2H, *J*= 6.0 Hz), 6.47 (t, 1H, *J* = 6.0 Hz), 6.52 (s, 1H), 6.61 (t, 1H, *J*= 6.0 Hz), 7.39 (m, 4H), 7.75 (s, 1H), 8.09 (s, 1H). HPLC purity 96.6%. ESI-MS *m/z*: 303.2 $[M + H]^+$.

Synthesis of ethyl 2-(3-(benzofuran-5-ylmethyl)ureido)acetate **(S54).**

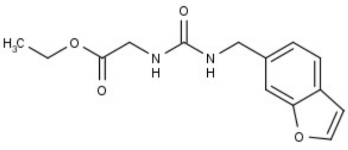

S54

The crude product was purified by flash chromatography (EtOAc) and finally precipitated in EDP to afford the urea **S54** (29 mg; 14%) as a white solid. TLC: Rf=0.55 (AcOEt). ^{1}H NMR (DMSO): δ 1.19 (t, 3H, *J*= 7.1 Hz), 3.79 (d, 2H, *J*= 5.7 Hz), 4.08 (q, 2H, *J* = 7.1 Hz), 4.29 (d, 2H, *J* = 5.9 Hz), 6.28 (t, 1H, *J*= 5.7 Hz), 6.70 (t, 2H, *J*= 5.9 Hz), 6.93 (s, 1H), 7.21 (d, 1H, *J*= 8.7 Hz), 7.53 (m, 2H), 8.00 (s, 1H). HPLC purity 98.5%. ESI-MS *m/z*: 277.2 $[M + H]^+$.

Synthesis of ethyl 2-(3-((1H-benzo[*d*]imidazol-2-yl)methyl)ureido)acetate **(S55).**

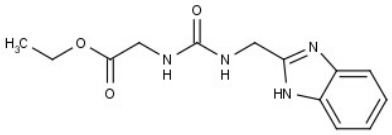

S55

The residue was taking up with AcOEt, the organic phase was washed with a solution of 10% citric acid and brine, dried over Na_2SO_4, filtrated and concentrated. **S55** was purified by precipitation in EDP to afford 95 mg of a yellow solid (44%). TLC: Rf=0.89 (AcOEt). ^{1}H NMR (DMSO): δ 1.19 (t, 3H, *J*= 7.1 Hz), 3.82 (d, 2H, *J*= 5.9 Hz), 4.08 (q, 2H, *J*= 7.1 Hz), 4.43 (d, 2H, *J*= 5.7 Hz), 6.52 (t, 1H, *J* = 5.9 Hz), 6.83 (t, 1H, *J*= 5.7 Hz), 7.13 (m, 2H), 7.45 (d, 1H, *J*= 5.7 Hz), 7.54 (d, 1H, *J*= 5.7 Hz), 12.16 (s, 1H). HPLC purity 98.9%. ESI-MS *m/z*: 277.2 $[M + H]^+$.

Synthesis of ethyl 2-(3-((5-methylisoxazol-3-yl)methyl)ureido)acetate **(S57).**

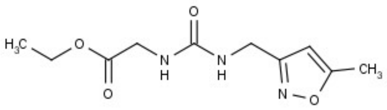

S57

S57 was purified by precipitation in EDP to afford 196 mg of a white solid (95%). TLC: Rf=0.47 (AcOEt). ^{1}H NMR (DMSO): δ 1.19 (t, 3H, *J*= 7.1 Hz), 2.36 (s, 3H), 3.77 (d, 2H, *J*= 6.0 Hz), 4.10 (q, 2H, *J*= 7.1 Hz), 4.19 (d, 2H, *J*= 6.0 Hz), 6.07 (s, 1H), 6.38 (t, 1H, *J* = 6.0 Hz), 6.70 (t, 1H, *J*= 6.0 Hz). HPLC purity 99.3%. ESI-MS *m/z*: 242.2 $[M + H]^+$.

Synthesis of **2-(3-(2-(5-amino-1,3,4-thiadiazol)methyl)ureido)acetate (S58).**

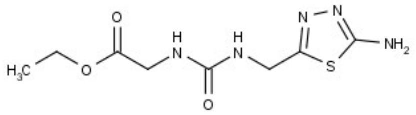

S58

S58 was purified to afford 86 mg of a yellow solid (68%). ^{1}H NMR (DMSO): δ 1.20 (t, 3H, *J*= 7.1 Hz), 3.78 (d, 2H, *J* = 6.0 Hz), 4.10 (q, 2H, *J* = 7.1 Hz), 4.31 (d, 2H, *J* = 6.0 Hz), 6.43 (t, 1H, *J* = 6.0 Hz), 6.92 (t, 1H, *J* = 6.0 Hz), 7.06 (s, 2H). HPLC purity 95.70%. ESI-MS *m/z*: 260.1 $[M + H]^+$.

Synthesis of ethyl 2-(3-(2-(2-(5-amino-1,3,4-thiadiazol)ethyl))ureido)acetate **(S59).**

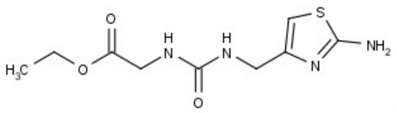

S59

S59 was purified to afford 87 mg of a yellow solid (64%). ^{1}H NMR (DMSO): δ 1.19 (t, 3H, *J*= 7.1 Hz), 2.88 (t, 2H, *J* = 6.7 Hz), 3.29 (m, 2H), 3.74 (d, 2H, *J* = 6.0 Hz), 4.09 (q, 2H, *J* = 7.1 Hz), 6.32 (m, 2H), 7.03 (s, 2H). HPLC purity 98.1%. ESI-MS *m/z*: 274.1 $[M + H]^+$.

Synthesis of ethyl 2-(3-(4-hydroxyphenylethyl)ureido)acetate **(S60).**

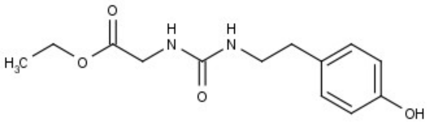

S60

S60 was purified by precipitation in EDP to afford 201 mg of a white solid (98%). TLC: Rf=0.32 (AcOEt). ^{1}H NMR (DMSO): δ 1.19 (t, 3H, *J*= 7.1 Hz), 2.55 (t, 2H, *J*= 7.3 Hz), 3.15 (q, 2H, *J*= 6.8 Hz), 3.75 (d, 2H, *J* = 6.0 Hz), 4.08 (q, 2H, *J* = 7.1 Hz), 6.11 (t, 1H, *J* = 6.0 Hz), 6.21 (t, 1H, *J* = 6.0 Hz), 6.68 (d, 2H, *J*= 8.3 Hz), 6.99 (d, 1H, *J*= 8.3 Hz), 9.16 (s, 1H). HPLC purity 99.4%. ESI-MS *m/z*: 267.2 [M + H]$^+$.

Synthesis of ethyl 2-(3-(2-(4-amino-6-hydroxypyrimidin-2-yl)ethyl)ureido)acetate (S61).

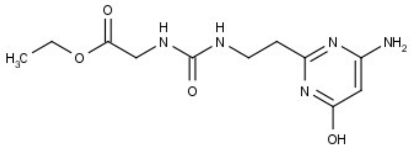

S61

The reaction mixture was heat at 70°C for 2 hours in DMF. The crude product was purified by flash chromatography (EtOAc/MeOH 7/3) to afford the urea **S61** (52 mg; 33%) as a white solid. TLC: Rf=0.24 (AcOEt/MeOH 7/3). ^{1}H NMR (DMSO): δ 1.19 (t, 3H, *J*= 7.1 Hz), 2.54 (m, 2H), 3.08 (m, 2H), 3.75 (d, 2H, *J* = 6.0 Hz), 4.07 (q, 2H, *J* = 7.1 Hz), 4.86 (s, 1H), 6.33 (m, 2H), 6.36 (s, 2H), 11.35 (s, 1H). HPLC purity 99.4%. ESI-MS *m/z*: 284.3 [M + H]$^+$.

Synthesis of ethyl 2-(3-(2-(2-(5-amino-1,3,4-thiadiazol)ethyl))ureido)acetate **(S62).**

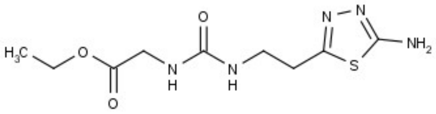

S62

S63 was purified to afford 87 mg of a yellow solid (64%). ^{1}H NMR (DMSO): δ 1.19 (t, 3H, *J*= 7.1 Hz), 2.88 (t, 2H, *J* = 6.7 Hz), 3.29 (m, 2H), 3.74 (d, 2H, *J* = 6.0 Hz), 4.09 (q, 2H, *J* = 7.1 Hz), 6.32 (m, 2H), 7.03 (s, 2H). HPLC purity 98.1%. ESI-MS *m/z*: 274.1 $[M + H]^+$.

Synthesis of ethyl 2-(3-(2-(2-(4-amino-1,3-thiazol)ethyl)ureido)acetate **(S63).**

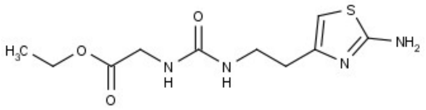

S63

S63 was purified to afford 64 mg of a yellow solid (48%). ^{1}H NMR (DMSO): δ 1.19 (t, 3H, *J*= 7.1 Hz), 3.22 (m, 2H), 3.34 (m, 2H), 3.74 (d, 2H, *J* = 6.0 Hz), 4.09 (q, 2H, *J* = 7.1 Hz), 6.13 (m, 2H), 6.23 (t, 1H, *J* = 6.0 Hz), 6.82 (s, 2H). HPLC purity 96.3%. ESI-MS *m/z*: 273.1 $[M + H]^+$.

Synthesis of ethyl 2-(3-(2-morpholinoethyl)ureido)acetate **(S64).**

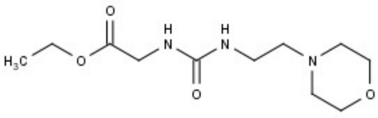

S64

S64 was purified by precipitation in EDP to afford 201 mg of a white solid (93%). ^{1}H NMR (DMSO): δ 1.19 (t, 3H, *J*= 7.1 Hz), 2.32 (t, 2H, *J* = 6.7 Hz), 2.36 (s, 4H), 3.11 (dd, 2H, *J* = 12.4, 6.2 Hz), 3.58 (s, 4H), 3.75 (d, 2H, *J* = 5.7 Hz), 4.08 (q, 2H, *J*= 7.1 Hz), 6.07 (t, 1H, *J* = 5.7 Hz), 6.35 (s, 1H). HPLC purity 96.8%. ESI-MS *m/z*: 260.2 $[M + H]^+$.

Synthesis of ethyl 2-(3-(3-(5-oxo-4,5-dihydro-1*H*-pyrazol-4-yl)propyl)ureido)acetate **(S65).**

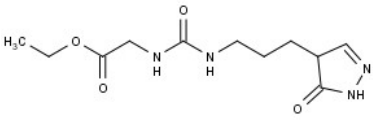

S65

S65 was purified by precipitation in EDP to afford 208 mg of a white solid (96%). ^{1}H NMR (DMSO): δ 1.21 (m, 3H), 1.57 (m, 2H), 2.17 (t, 2H, *J*= 7.4 Hz), 3.00 (m, 2H), 3.75 (d, 2H, *J* = 6.0 Hz), 3.96 (m, 1H), 4.09 (m, 2H), 4.14 (q, 2H, *J* = 7.1 Hz), 6.16 (s, 1H), 6.21 (s, 1H), 9.35 (s, 1H). HPLC purity 99.6%. ESI-MS *m/z*: 271.2 $[M + H]^+$.

Synthesis of ethyl 2-(3-(piperidin-4-ylmethyl)ureido)acetate **(S66).**

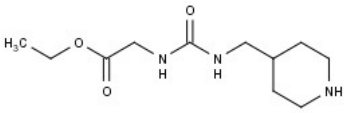

S66

The crude product was purified by HPLC preparative to afford the urea **S66** (92 mg; 39%) as a white solid. ^{1}H NMR (DMSO): δ 1.19 (m, 5H), 1.59 (s, 1H), 1.72 (d, 2H, *J*= 13.3 Hz), 2.78 (t, 2H, *J*= 12.1 Hz), 2.92 (t, 2H, *J* = 6.1 Hz), 3.22 (d, 2H, *J*= 12.1 Hz), 3.75 (d, 2H, *J*= 6.0 Hz), 4.08 (q, 2H, *J* = 7.2 Hz), 6.17 (t, 1H, *J*= 6.1 Hz), 6.29 (t, 1H, *J*= 6.0 Hz). HPLC purity 99.5%. ESI-MS *m/z*: 244.2 $[M + H]^+$.

Synthesis of ethyl 2-(3-((tetrahydro-*2H*-pyran-4-yl)methyl)ureido)acetate **(S67).**

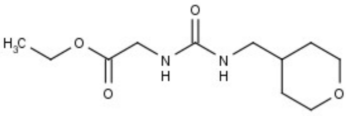

S67

S67 was purified by precipitation in EDP to afford 66 mg of a white solid (35%). TLC: Rf=0.83 (AcOEt). ^{1}H NMR (DMSO): δ 1.05 (m, 2H), 1.19 (t, 3H, *J*= 7.1 Hz), 1.51 (m, 3H), 2.89 (t, 2H, *J* = 6.0 Hz), 3.24 (t, 2H, *J* = 11.3 Hz), 3.75 (d, 2H, *J* = 6.0 Hz), 3.83 (dd, 2H, *J* = 10.3; 3.4 Hz), 4.08 (q, 2H, *J* = 7.1 Hz), 6.11 (t, 1H, *J* = 6.0 Hz), 6.22 (t, 1H, *J* = 6.0 Hz). HPLC purity 95.2%. ESI-MS *m/z*: 245.2 $[M + H]^+$.

Synthesis of 2-(3-(3-acetamidobenzyl)ureido)acetate (S40).

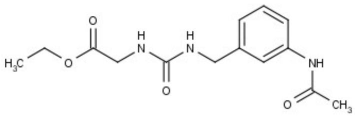

S40

Amine derivative **S21** (1 equivalent) was dissolved in 2 ml of DCM. Acetyl chloride (1 equivalent) was added and the reaction mixture is stirred for 20 h at room temperature. The reaction mixture is concentrated and 50 ml of AcOEt are added. The organic phase are washed with $NaHCO_3$ saturated, 10% citric acid and brine then dried over Na_2SO_4, filtered and concentrated. The crude product was purified by flash chromatography (AcOEt/MeOH 95/5) to afford the amide **S40** (17 mg; 17%) as a white solid. TLC: Rf=0.16 (AcOEt/MeOH 95/5). ^{1}H NMR (DMSO): δ 1.18 (t, 3H, *J* = 7.1 Hz), 2.03 (s, 3H), 3.82 (d, 2H, *J* = 6.0 Hz), 4.08 (q, 2H, *J* = 7.1 Hz), 4.22 (d, 2H, *J* = 6.4 Hz), 6.42 (t, 1H, *J* = 6.0 Hz), 6.95 (t, 1H, *J* = 9.0 Hz), 7.13 (t, 2H, *J* = 6.4 Hz), 7.30 (q, 1H, *J* = 8.2 Hz), 7.78 (d, 1H, *J* = 8.2 Hz), 10.52 (s, 1H). HPLC purity 97.7%. ESI-MS *m/z*: 294.3 $[M + H]^+$.

Synthesis of ethyl 2-(3-(6-amino-2,3-dihydro-1H-inden-1-yl)ureido)acetate **(32).**

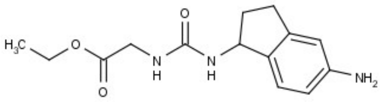

32

2,3-dihydro-1H-indene-1,6-diamine (1eq, 100 mg, 0.46 mmol) and triethylamine (2.5 eq, 158 µL, 1.15 mmol) were dissolved in 2 mL of DMF. The reaction mixture was cooled at 0°C and ethyl isocyanatoacetate (1 eq, 59 mg, 51 µL, 0.46 mmol) was added dropwise and stirred for 2 hours at 0°C. The reaction mixture was concentrated and purified on reverse phase (H_2O/MeCN) to afford the compound (26 mg, 21%) as a white solid Rf=0.26. ^{1}H NMR (300 MHz, DMSO): δ 6.91 (d, *J* = 7.8, 1H), 6.47-6.39 (m, 2H), 6.36 (d, *J* = 8.3 Hz, 1H), 6.11 (t, *J* = 5.9 Hz, 1H), 5.02-4.88 (m, 3H), 4.13 (q, *J* = 7.1 Hz, 2H), 3.83 (d, *J* = 6.0, Hz 2H), 2.87-2.57 (m, 2H), 2.40-2.25 (m, 1H), 1.72.1.56 (m, 1H), 1.31-1.17 (t, *J* = 7.2 Hz, 3H). ESI-MS *m/z*: 278.3 [M + H]$^+$.
